# Pyridinium-Fused 1,3-Selenazoles via Cyclizations of 2-Pyridylselenyl Chloride with Alkynes: Synthesis, Structures, and Antifungal Properties

**DOI:** 10.3390/ijms27062908

**Published:** 2026-03-23

**Authors:** Evgeny A. Dukhnovsky, Alexey S. Kubasov, Olga G. Chusova, Victor N. Khrustalev, Alexander V. Borisov, Francis Verpoort, Rosa M. Gomila, Antonio Frontera, Zhishen Ge, Alexander G. Tskhovrebov

**Affiliations:** 1 Peoples’ Friendship University of Russia, 6 Miklukho-Maklaya Street, 117198 Moscow, Russia; 2 Kurnakov Institute of General and Inorganic Chemistry, Russian Academy of Sciences, Leninsky Prosp. 31, 119071 Moscow, Russia; 3 Nesmeyanov Institute of Organoelement Compounds, Russian Academy of Sciences, 119991 Moscow, Russia; 4 N.D. Zelinsky Institute of Organic Chemistry, Russian Academy of Sciences, Leninsky Prosp. 47, 119334 Moscow, Russia; 5 R.E. Alekseev Nizhny Novgorod State Technical University, Minin St. 24, 603950 Nizhny Novgorod, Russia; avb1955@rambler.ru; 6 State Key Laboratory of Advanced Technology for Materials Synthesis and Processing, Wuhan University of Technology, Wuhan 430070, China; 7 Department of Chemistry, Universitat de les Illes Balears, Crta. de Valldemossa km 7.5, 07122 Palma de Mallorca, Spaintoni.frontera@uib.es (A.F.); 8 School of Chemistry, Xi’an Jiaotong University, Xi’an 710049, China

**Keywords:** organoselenium, alkynes, regioselectivity, chalcogen bonding, antifungal activity, phytopathogenic fungi, DFT calculations

## Abstract

We report a straightforward and versatile synthetic route to pyridinium-fused 1,3-selenazoles via the electrophilic cyclization of 2-pyridylselenyl chloride with alkynes. The reaction proceeds efficiently under mild conditions with representative terminal and internal alkynes. While the cyclization exhibits high regioselectivity favoring the 3-substituted isomer for most substrates, reactions with 2-pyridyl- and 2-quinolylacetylenes yield regioisomeric mixtures. DFT calculations rationalize this divergence, revealing a competition between kinetic and thermodynamic control; the 3-isomer is kinetically favored, while the 2-isomer is thermodynamically stabilized by an ancillary chalcogen bond between the selenium atom and the pyridine nitrogen of the alkyne substituent. Molecular structures were confirmed by single-crystal X-ray diffraction, and the non-covalent interactions governing supramolecular assembly in the solid state were rigorously analyzed using MEP surfaces, the QTAIM, and NBO analysis. Antifungal evaluation identified several compounds with notable activity against phytopathogenic fungi, highlighting the potential of this novel heterocyclic scaffold in agrochemical applications.

## 1. Introduction

Organoselenium chemistry has recently gained significant attention due to its remarkable versatility and wide-ranging applications across various fields, including medicine [1,2,3], field-effect transistors [4,5,6,7], and photovoltaics [8,9,10]. Among organoselenium compounds, 1,3-selenazoles represent an important class of aromatic heterocycles that, despite their importance, remain far less explored compared to their oxygen- and sulfur-containing analogs. The 1,3-selenazole framework is a prevalent structural motif in various compounds displaying biological activity and advanced functional materials [11,12,13,14,15]. This heterocyclic scaffold exhibits a broad spectrum of biologically relevant activities such as antiviral [16,17,18], antibacterial [19,20,21], anticancer [22,23,24], antiparasitic [25], and neuroprotective [26,27] effects and inhibitory activity against xanthine oxidase [28,29]. These significant properties have stimulated the development of novel synthetic methodologies targeting 1,3-selenazoles to unlock their full potential in medicinal and material science applications.

**Scheme 1:**
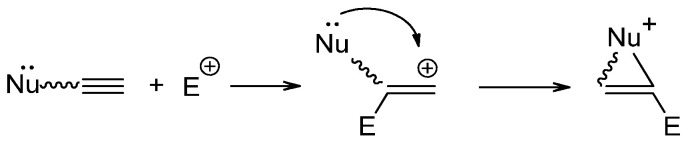
Electrophilic cyclization of alkynes.

Electrophilic cyclization of alkynes offers a powerful strategy for constructing unsaturated heterocycles, wherein ring closure is initiated by an electrophile and facilitated by nucleophilic attack from a heteroatom within the substrate (Scheme 1). In this context, selenium-based electrophiles have proven to be very convenient and useful reagents for this type of cyclization [30,31,32,33].

Another pathway for ring formation involving addition reactions at multiple bonds, classified as cycloaddition, has been explored in significantly less detail, likely due to the limited availability of suitable selenium-containing reagents. Selenenyl halides among Se-centered electrophiles offer a compelling approach to synthesizing heterocycles. The structural motif of the cationoid part in these reagents presents opportunities for transformation, facilitating the introduction of potentially nucleophilic centers. This allows for ring closure upon reaction with unsaturated compounds. Our research group has demonstrated that incorporating a nitrogen-centered basic fragment into the organylselenyl moiety enables a novel reaction of selenyl halides with unsaturated compounds, specifically facilitating cycloaddition at multiple bonds by a selenium-centered electrophile [34,35].

Recently, the effectiveness of this approach for synthesizing selenium- and nitrogen-containing heterocycles has been demonstrated using various unsaturated substrates, including alkenes (C=C bonds) [36,37,38,39], isoselenocyanates [40], isocyanates (C=N bonds) [41], nitriles [42,43] or selenocyanates (C≡N bonds) [40] and nitriles featuring acidic α-methylene moiety [44]. These reactions, employing bifunctional 2-pyridylselenyl reagents, show the versatility of this approach in constructing pyridinium-fused heterocyclic frameworks—structures that remain inaccessible via other synthetic methods.

The electrophilic addition reactions of ArSeCl (including 2-PySeCl) to alkenes have been extensively studied, and this area remains a subject of significant interest. In contrast, the addition of 2-PySeCl to alkynes has been far less explored, with only a limited number of studies addressing this reaction [45]. This research gap presents a valuable opportunity for further investigation into the reactivity and potential applications of selenyl reagents in alkyne chemistry and synthesis of Se-containing heterocycles.

Here we describe a straightforward and convenient route to pyridinium-fused 1,3-selenazoles. Beyond the synthetic scope, we provide a detailed theoretical investigation to rationalize the divergent regiochemistry observed with pyridine-substituted alkynes. Moreover, antifungal evaluation screening was performed, which demonstrated high activity for some derivatives against phytopathogenic fungi. The details are presented hereinafter.

## 2. Results and Discussion

**Scheme 2:**
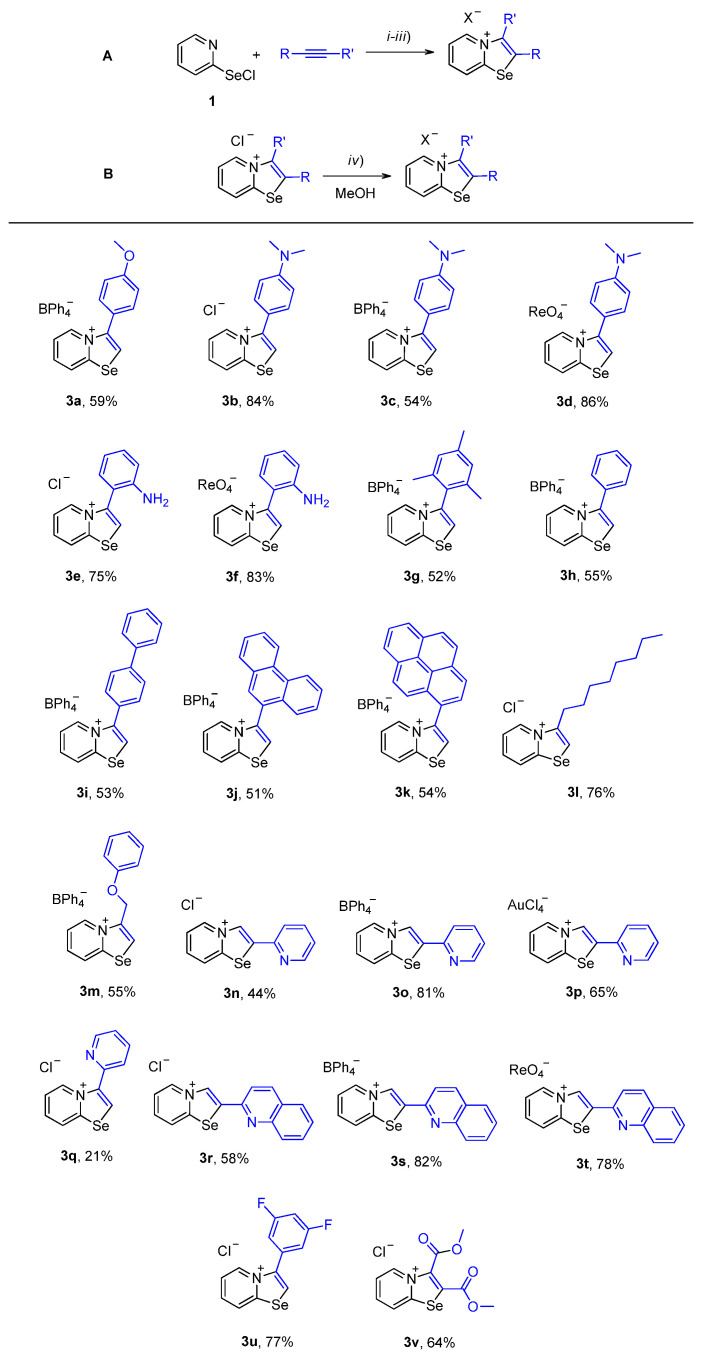
Synthesis of **3a**–**3v**. (*i*) MeOH, 65 °C, 3 h, NaBPh_4_ (excess) (**3a**, **3c**, **3g**–**3k**, **3m**); (*ii*) CH_2_Cl_2_, 20 °C, 48 h (**3b**, **3e**, **3n**, **3q**, **3r**, **3u**, **3v**); (*iii*) 1,2-DCE, 84 °C, 3 h (**3l**); (*iv*) MeOH, 20 °C, 24 h, NH_4_ReO_4_ (1 equiv) (**3d**, **3f**, **3t**), NaBPh_4_ (1 equiv) (**3o**, **3s**), NaAuCl_4_·2H_2_O (1 equiv) (**3p**). (**A**): cycloaddition reaction between pyridylselenyl reagents and alkynes. (**B**): Anion exchange in selenazolium salts.

[1,3]-Selenazolo-[3,2-a]-pyridin-4-ium salts **3a**–**3v** featuring (BPh_4_)^−^, (AuCl_4_)^−^, (ReO_4_)^−^ and Cl^−^ anions were synthesized via a cycloaddition reaction between ambiphilic pyridylselenyl reagents and corresponding alkynes in a 1:1 molar ratio (Scheme 2, Experimental Section).

While the direct addition of **1** to alkynes provided the desired pyridinium-fused selenazolium salts only for specific substrates (**3b**, **3e**, **3l**, **3n**, **3q**, **3r**, **3u**, and **3v**), a general and high-yielding synthesis was achieved by performing the reaction in the presence of excess NaBPh_4_ in methanol. Under these optimized conditions, the target selenazolium salts precipitate cleanly as their tetraphenylborate salts, facilitating isolation (Scheme 2A). This protocol demonstrated reactivity with representative terminal and internal alkynes of diverse electronic nature, including electron-rich, electron-poor, and polycyclic aromatics, as well as aliphatic systems (Scheme 2B).

The cyclization proceeded with high regioselectivity for most alkynes, invariably placing the alkyne-derived substituent at the 4-position of the selenazole ring (Scheme 2). The notable exceptions were 2-pyridyl- and 2-quinolylacetylenes, which afforded mixtures of two regioisomers.

**Scheme 3:**
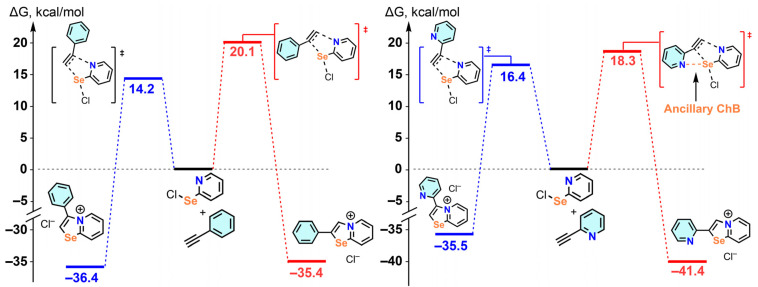
Calculated Gibbs free energy profiles (ΔG in kcal/mol) for the regioselective cyclization of ethynylbenzene (**left**) and 2-ethynylpyridine (**right**). The pathways leading to the 3-substituted selenazolopyridinium products are shown in blue, while those leading to the 2-substituted isomers are shown in red. All stationary points were optimized at the BP86-D4/def2-TZVP level of theory with solvation effects (methanol or DCM) modeled using CPCM.

**Figure 1:**
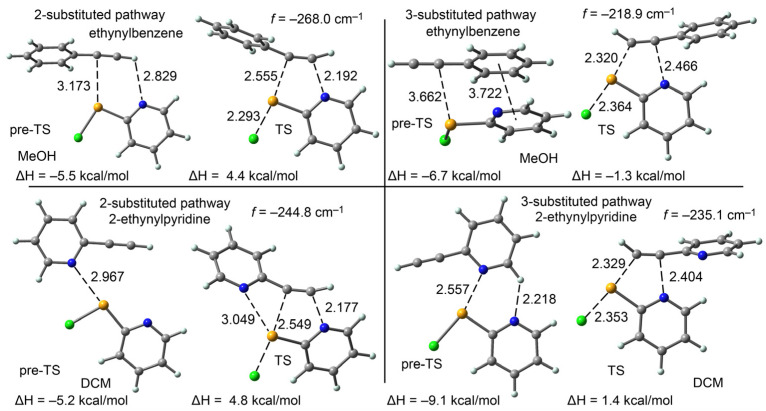
Optimized geometries of the transition states (TS) and the preceding pre-reaction complexes (pre-TS), corresponding to the endpoints of the intrinsic reaction coordinate (IRC) pathway. Key interatomic distances are given in Å. Calculations were performed under standard conditions with implicit solvation models (methanol for ethynylbenzene, upper panel; dichloromethane for 2-ethynylpyridine, lower panel). The imaginary frequencies characterizing each transition state are also indicated.

To rationalize the divergent regiochemistry observed experimentally, we performed DFT calculations on the cyclization of ethynylbenzene and 2-ethynylpyridine (Scheme 3). For ethynylbenzene, the formation of the 3-phenyl-selenazolopyridinium (blue pathway) is both kinetically and thermodynamically favored over the 2-phenyl isomer. The difference in activation free energy between the two transition states is 5.9 kcal/mol, which is consistent with the exclusive formation of the 3-substituted product. In contrast, the reaction with 2-ethynylpyridine reveals a competition between kinetics and thermodynamics. While the route yielding the 3-pyridyl isomer remains kinetically favored, the energy difference between the competing transition states drops significantly to only 1.9 kcal/mol. Furthermore, the formation of the 2-pyridyl isomer (red pathway) is thermodynamically favored by 5.9 kcal/mol. The lower energy of the transition state leading to the 2-isomer, as well as the increased product stability, is attributed to the formation of an ancillary chalcogen bond between the pyridine nitrogen atom and the selenium center. This specific interaction accounts for the formation of regioisomeric mixtures with 2-pyridyl- and 2-quinolylacetylenes, distinguishing them from their carbocyclic counterparts. In both cases, the formation of pre-reaction complexes that correspond to the extreme points of the IRC trajectories is observed. The geometric features and enthalpies of the pre-reaction complexes and transition states are given in Figure 1.

The optimized geometries of the pre-reaction complexes and transition states shown in Figure 1 highlight the key role of Se···N chalcogen bonding in stabilizing the complexes formed with 2-ethynyl pyridine (lower panel). In contrast, the complexes involving ethynylbenzene (upper panel) are stabilized primarily by Se···π(C≡C) and π···π stacking interactions. While the ΔG values for the pre-reaction complexes (pre-TS) are positive, the corresponding Δ values are negative, as expected for the formation of supramolecular assemblies. Notably, when entropic effects are excluded from the calculations, one of the transition states even shows a negative ΔH relative to the separated reactants.

**Figure 2:**
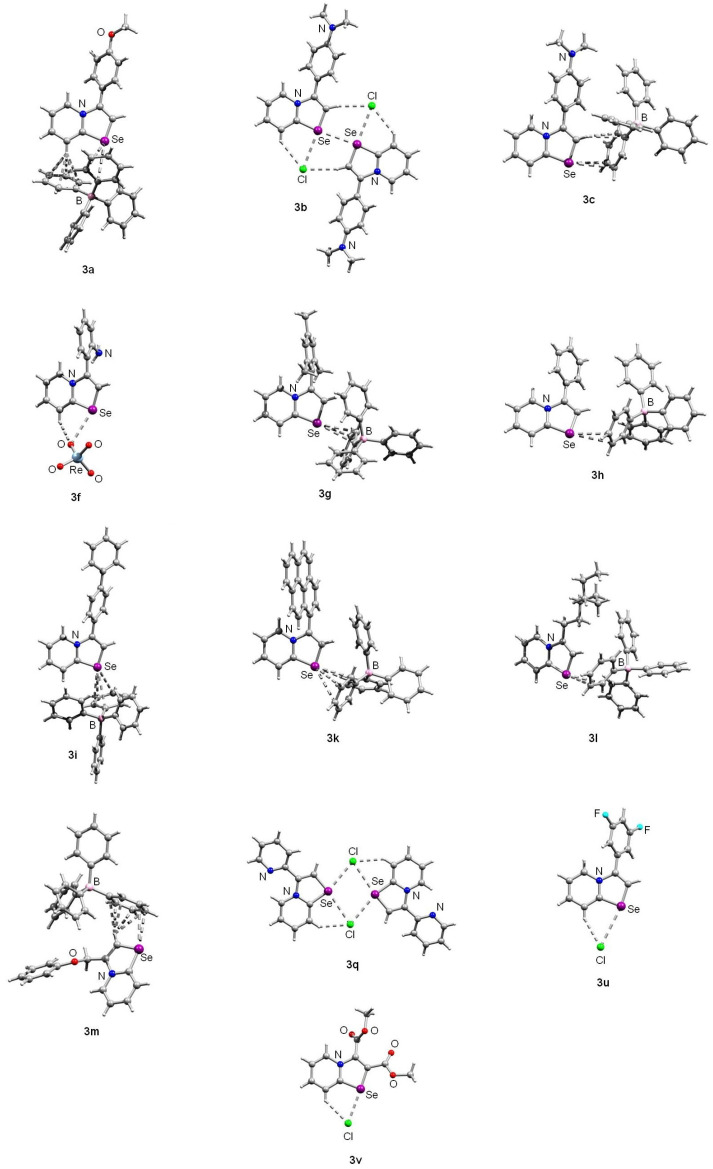
Ball-and-stick representations of the crystal structures of **3a**–**3v** demonstrating Se-centered ChB. Grey and light-grey spheres represent carbon and hydrogen, respectively.

All the new compounds were thoroughly characterized using ^1^H and ^13^C NMR spectroscopy, HRMS and IR spectroscopy. Additionally, most of the compounds were successfully recrystallized from a CH_2_Cl_2_-Et_2_O mixture, yielding high-quality single crystals suitable for X-ray structural analysis. The crystallographic data unequivocally confirmed the molecular structures of the compounds (CCDC 2524056-2524074, Figure 2).

The bicyclic heterocyclic systems in **3a**–**3v** are virtually planar (RSMD is in 0.009−0.029 Å range). The selenium atom in **3a**–**3v** adopted a T-shaped geometry, with the C1−Se−C7 being slightly less than 90° (Table S2). The Se−C1 separation (1.852(6)−1.876(3) Å) and Se−C7 separation (1.849(4)−1.895(10) Å) for **3a**–**3v** is less than the standard single C−Se bond distance (1.90 Å) but longer than the double Se=C bond distance (1.82 Å), indicating back-donation of the selenium electron pairs to the pyridine ring and the 1,3-selenazole fragment. The C6–N distance was in the interval between 1.401(3) Å and 1.447(7) Å and was typical for the C–N single bond. The C=C separation (1.328(15)−1.359(13) Å) corresponded to a typical double bond (Table S1).

The solid-state structures of salts **3a**, **3c**, and **3g**–**3m** featuring (BPh_4_)^−^ anions exhibited multiple Se⋯π ChB contacts with 1,3-selenazolium cations; however, the geometry of Se⋯π interactions in all cases was slightly different (Figure 2). For compounds **3a** and **3i** the π-systems of the phenyl rings of BPh_4_^−^ anion approached the selenium atoms along the Se-C7 bond extension, while, in the other cases, the aromatic rings were directed along the axis of the Se-C1 covalent bond of the chalcogen-containing adducts. In addition to the presence of revealed Se⋯π interactions, which are of the Se⋯aromatic ring–face interaction type, crystal structures of some compounds, such as **3a**, **3c**, and **3m**, were further stabilized by weak intermolecular C−H⋯π interactions between the proton of the heterocycle and the phenyl ring of the anion (Figure 2).

1,3-Selenazolium cations in adducts **3b** and **3q**–**3v** expectedly interacted with the Cl^−^ anions via a pair of “chelating” Se⋯Cl and H⋯Cl non-covalent interactions, forming a typical supramolecular synthon (Figure 2), previously observed in similar Se-containing heterocycles [42,43,44,46,47,48,49,50,51]. Interestingly, compound **3b** self-assembled into supramolecular dimers in the solid state via Se⋯Se, Se⋯Cl ChB and H⋯Cl HB interactions. It should be noted that, for the earlier described adducts of 2-pyridylselenyl reagents with unsaturated substrates, dimer formation via short Se⋯Se contact (3.347 Å) was not observed. Compound **3q** also formed dimers via four Se···Cl ChB interactions with two bridging Cl^−^ anions. In compound **3f**, non-covalent “chelation” of the ReO_4_^−^ anion via Se⋯O ChB and H⋯Cl HB interactions was observed (Figure 1).

To obtain single crystals suitable for the X-ray structural analysis and to study self-assembly and supramolecular self-organization of chemical systems in the solid state, a series of salts of (BPh_4_)^−^ (**3o**, **3s)**, (AuCl_4_)^−^ (**3p**) and (ReO_4_)^−^ (**3d**, **3f**, **3t**) were prepared via anion metathesis in [1,3]selenazolo [3,2-a]pyridin-4-ium chlorides at 20 °C for 24 h in MeOH (Scheme 2B).

**Figure 3:**
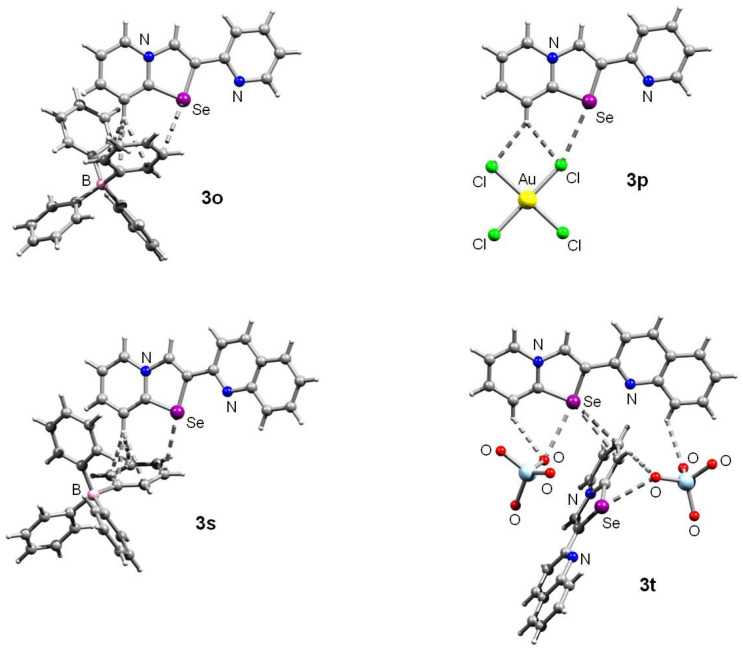
Ball-and-stick representations of the crystal structures of **3o**, **3p**, **3s** and **3t** demonstrating Se-centered ChB. Grey and light-grey spheres represent carbon and hydrogen, respectively.

The chloride salts **3n** and **3r** failed to produce X-ray quality single crystals; however, BPh_4_^−^, AuCl_4_^−^ and ReO_4_^−^ salts (**3o**, **3s**, **3p** and **3t**, respectively) easily crystallized to give large monocrystals, suitable for X-ray structural analysis, which confirmed the regioselective formation of the adducts (Figure 3).

Analysis of the crystal structures of **3o** and **3s** showed the presence of interactions of (BPh_4_)^−^ anions with the 1,3-selenazolium cations via the combination Se⋯π ChB and C−H⋯π contacts. For the pyridyl-substituted (AuCl_4_)^−^ salt of 1,3-selenazole **3p**, the selenium atom participated in Se⋯Cl ChB with the anion, and a bifurcated H⋯Cl HB between the proton and two Cl atoms was also observed. In case of the anion substitution of (BPh_4_)^−^ to (ReO_4_)^−^ in the quinolyl-substituted salt of 1,3-selenazole led to self-assembly of the adduct **3t** into 1D zig-zag supramolecular chains along the a-axis via Se⋯O between the anion and the Se⋯π ChBs pyridine ring of the adjacent cation (Figure 3).

**Figure 4:**
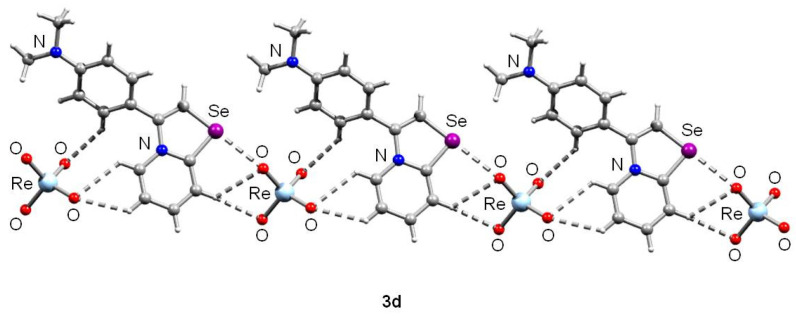
Ball-and-stick representations of the crystal structure of **3d**, demonstrating its self-assembly into 1D supramolecular polymer via Se⋯O and five H⋯O interactions.

The perrhenate salt **3d** self-assembled into 1D supramolecular polymer along the c-axis via Se⋯O ChB and H⋯O HB in the solid state (Figure 4). Bifurcated H⋯O HB was observed between the H atom of the pyridine ring and the ReO_4_^−^ anion with involved Se⋯O ChB, which formed a robust supramolecular synthon.

**Scheme 4:**
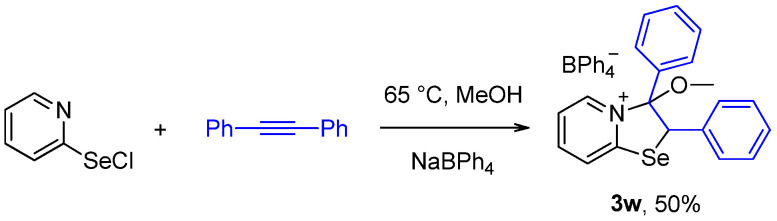
Synthesis of **3w**.

The reaction of 2-pyridylselenyl chloride with diphenylacetylene was carried out at 65 °C for 3 h in MeOH with excess NaBPh_4_ to give product **3w** in 50% yield. However, in this case, a methanolysis process was observed, involving the addition of a nucleophile to the carbon atom bonded to the nitrogen of the pyridine ring (Scheme 4).

**Figure 5:**
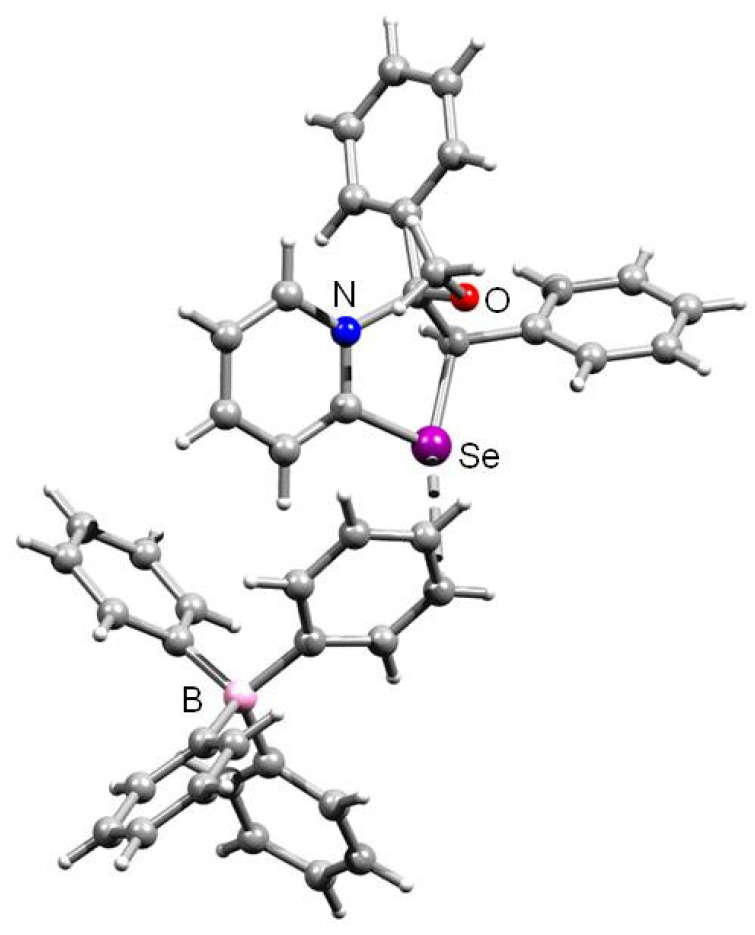
Ball-and-stick representations of the crystal structure of **3w**. Grey and light-grey spheres represent carbon and hydrogen, respectively.

The resulting adduct **3w** obtained in 50% yield was structurally characterized by means of single-crystal X-ray analysis (Figure 5).

Although anion-π interactions involving the aromatic surfaces are undoubtedly relevant in the crystal packing of these salts, our theoretical analysis focuses specifically on the less explored chalcogen bonding interactions centered on the selenium atom. We selected the chloride, perrhenate, and tetrachloroaurate salts for this investigation, as they exhibit highly directional contacts compared to the bulky tetraphenylborate derivatives. To rigorously quantify these interactions, we first calculated the Molecular Electrostatic Potential (MEP) of a model unsubstituted selenazolopyridinium cation to assess the relative electrophilicity of the two σ-holes. Subsequently, we characterized the topology of the electron density in the relevant supramolecular assemblies using the Quantum Theory of Atoms in Molecules (QTAIM) and estimated the charge transfer contributions via Natural Bond Orbital (NBO) analysis.

**Figure 6:**
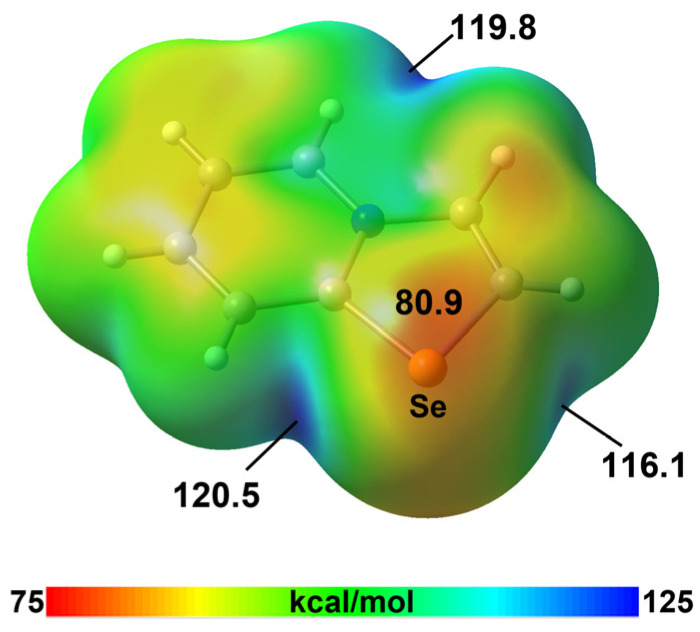
Molecular Electrostatic Potential (MEP) surface of the model unsubstituted selenazolopyridinium cation (isosurface 0.001 a.u.). The values at selected points of the surface are given in kcal/mol. The color scale ranges from +75 (red) to +125 (blue) kcal/mol. Calculations were performed at the PBE0-D4/def2-TZVP level of theory.

To assess the ability of the selenium atom to participate in chalcogen bonding, we computed the MEP surface of a model unsubstituted selenazolopyridinium cation (Figure 6). As expected for a cationic species (+1 charge), the MEP is positive over the entire molecular surface. However, the charge distribution around the selenium atom is highly anisotropic. The region corresponding to the selenium lone pairs appears red (less positive, ~80.9 kcal/mol), while two regions of high positive potential, corresponding to the sigma-holes, are located along the extensions of the C-Se bonds. The calculations reveal that the sigma-hole opposite to the Se-C(selenazole) bond is more positive (120.5 kcal/mol) than the one opposite to the Se-C(pyridine) bond (116.1 kcal/mol). This enhanced electrophilicity is attributed to the cooperative electrostatic contribution from the adjacent hydrogen atom of the pyridine ring. Additionally, the MEP surface exhibits high positive values (119.8 kcal/mol) in the regions surrounding the C-H bonds adjacent to the cationic nitrogen atom, reflecting the strong electron-withdrawing effect of the pyridinium core.

**Figure 7:**
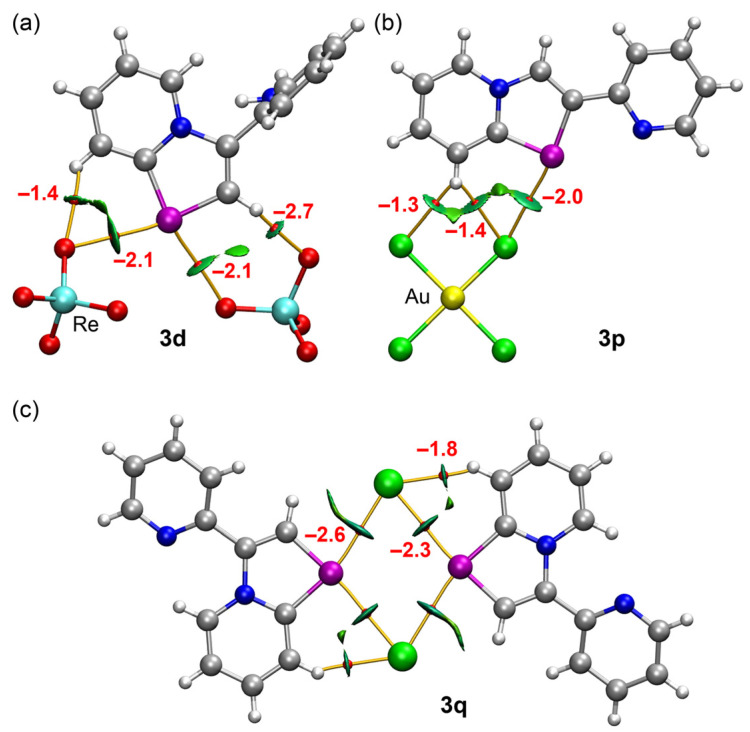
QTAIM/NCIplot analysis of selected supramolecular assemblies from the crystal structures of (**a**) **3d**, (**b**) **2p**, and (**c**) **3q**. The superposition of the bond critical points (BCPs, small red spheres) and bond paths (orange lines) with the NCIplot isosurfaces (s = 0.5 a.u.; color scale from –0.035 to 0.035 a.u.) is shown. The interaction energies, estimated using QTAIM-based predictors, are indicated in red next to the corresponding BCPs in kcal/mol.

The supramolecular architectures of compounds **3d**, **2p**, and **3q** were further analyzed using QTAIM and NCIplot computational tools (Figure 7). In these ionic systems, standard supermolecular binding energy calculations are dominated by strong, non-directional electrostatic forces between counterions, rendering them unsuitable for evaluating specific local contacts. Therefore, we estimated the strength of individual non-covalent interactions using predictors based on the electron density properties at the bond critical points (BCPs), as detailed in the computational methods. The resulting energy values are indicated in red adjacent to the corresponding BCPs. For the perrhenate salt **3d** (Figure 7a), the analysis reveals that both selenium σ-holes engage in chalcogen bonding with oxygen atoms of two neighboring anions. These ChBs are defined by BCPs, interatomic bond paths, and characteristic green reduced density gradient (RDG) isosurfaces, with both contacts showing identical interaction energies of –2.1 kcal/mol. Simultaneously, two CH···O hydrogen bonds—one involving the pyridine ring and another the selenazole ring—are established, characterized by typical disk-shaped RDG surfaces. The strengths of these HBs are comparable to the ChBs, indicating a synergistic contribution of both interaction types to the assembly’s stability. In contrast, for the tetrachloroaurate salt **2p** (Figure 7b), the bulky 2-pyridyl substituent sterically blocks one selenium σ-hole; consequently, only one ChB is formed with the AuCl_4_ anion (−2.0 kcal/mol). However, the analysis highlights a prominent bifurcated CH···Cl,Cl hydrogen bond with a combined interaction energy of −2.7 kcal/mol, suggesting that HBs are the dominant directing force in **2p**. Finally, the analysis of the chloride salt **3q** (Figure 7c) focused on a centrosymmetric dimer where two chloride anions bridge two cations via four ChBs. These Se···Cl interactions are notably stronger (−2.6 and −2.3 kcal/mol) than those involving the non-coordinating anions in **3d** and **2p**, reflecting the higher nucleophilicity of the chloride anion. Although ancillary CH···Cl contacts are present (−1.8 kcal/mol), the greater number and strength of the chalcogen bonds confirm their dominant role in the crystal packing of **3q**.

**Figure 8:**
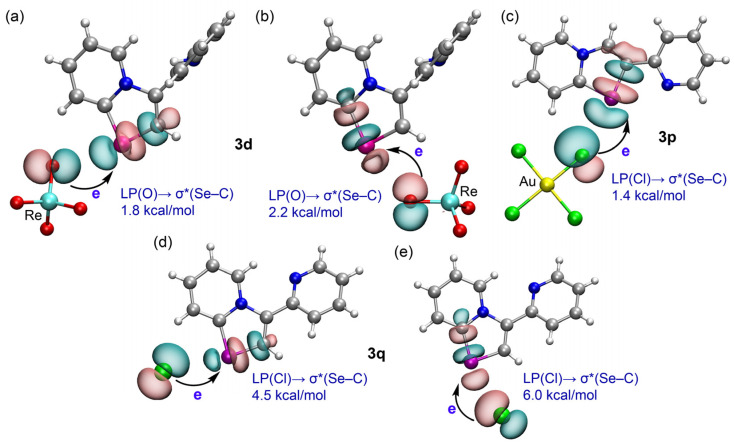
Natural Bond Orbital (NBO) analysis of the chalcogen bonding interactions in (**a**,**b**) **3d**, (**c**) **3p**, and (**d**,**e**) **3q**. The plots illustrate the overlap between the donor LP orbitals of the anions and the acceptor antibonding σ*(Se–C) orbitals of the cation. The E^(2)^ values are given in kcal/mol.

Finally, to assess the covalent contributions to the stability of these supramolecular assemblies, we performed Natural Bond Orbital (NBO) analysis (Figure 8). In all cases, the calculations confirm the presence of a characteristic charge transfer from a lone pair (LP) orbital of the anion (donor) to an antibonding σ*(Se–C) orbital of the selenazolopyridinium cation (acceptor). For the perrhenate salt **3d**, the second-order perturbation analysis yields stabilization energies (E^(2)^) of 1.8 and 2.2 kcal/mol for the LP(O) → σ*(Se–C) interactions, indicating a modest orbital overlap. A similarly weak interaction is observed for the tetrachloroaurate salt **3p**, where the LP(Cl) → σ*(Se–C) charge transfer provides a stabilization of only 1.4 kcal/mol. In sharp contrast, the chloride salt **3q** exhibits significantly stronger orbital interactions, with E^(2)^ values of 4.5 and 6.0 kcal/mol. This substantial increase in stabilization energy is consistent with the QTAIM results and reflects the higher nucleophilicity of the chloride anion compared to the oxygen and chlorine atoms in the complex ReO_4_^−^ and [AuCl_4_]^−^ anions, respectively.

### Antifungal Activity Evaluation

The fungicidal activity of imidazolium [1,3]-selenazolo-[3,2-a]-pyridin-4-ium salts **3a**–**3v** was tested in vitro with negative and positive controls treated with a reference commercial fungicide (Triadimefon). The list of target phytopathogenic fungi included those infecting various agricultural plants such as bananas, tomatoes and onions (*Fusarium oxysporum* (*F.o.*)); cereals (*Bipolaris sorokiniana (B.s.)*, syn. *Helminthosporium sativum*) and corn (*Fusarium moniliforme* (*F. m*.)); beet, tomatoes and potatoes (*Rhizoctonia solani* (*R.s.*)); apples (*Venturia inaequalis* (*V.i.*)); and soybeans and green beans (*Sclerotinia sclerotiorum (S.s.))*. The effect of compounds on the radial growth of mycelium was studied by counting the percentage of mycelium growth inhibition (MGI) at the concentration of 30 mg × L^−1^.

In a panel of low to moderately active pyridinium-fused 1,3-selenazoles (see Table 1 for details), **3b** and **3u** demonstrated specific targeting of *B.s.* (MGI 100 and 87%, respectively, as opposed to 73% for triadimefon). Two salts, **3l** and **3r**, stood out uniquely for their broader spectrum action and were especially active against *S.s.*, *V.i.* and *B.s.* (MGI 100/90, 100 and 100%, respectively, as opposed to 57, 61 and 73% for triadimefon) (Table 2). Remarkably, compounds **3b**, **3l** and **3r** completely suppressed the growth of *B.s.* even at a concentration as low as 15 mg × L^−1^.

High potency of **3l** is most likely attributed to its specific structure, i.e., the cationic part bearing long alkyl chain makes it amphiphilic. The amphiphilicity of such compounds endows them the ability to self-aggregate into micelles, vesicles and membranes in aqueous solutions [52]. The topic of this type of compound known as surface-active ionic liquids (SAILs) is steadily growing [53,54]. Imidazolium-based ILs are being extensively studied for their multifunctional applications, including antifungal [55]. It was previously shown that imidazole-based ionic liquids cause complete plasma wall separation and cytoplasmic extravasation in cells. Thus, the molecule enters the cell membrane of fungi through its alkyl chain tail and disturbs the arrangement of phospholipid bilayers, leading to a gradual loss of intracellular substances and cell inactivity [56].

In terms of factors influencing fungicidal activity, the tested salts can be divided into two groups based on anion type: tetraphenylborate and chloride. Two compound pairs –**3b** and **3c**, and **3r** and **3s**—differ only in their counterions, BPh4— and Cl—, respectively. Neither anion alone inhibited fungal growth at the concentrations used in this study. Notably, the chloride-containing compounds (**3b** and **3r**) exhibited strong antifungal activity, while their tetraphenylborate analogs (**3c** and **3s**) showed poor activity.

This finding contradicts previous reports suggesting that more hydrophobic or bulky anions in ionic liquids generally enhance antifungal efficacy when paired with the same low-molecular-mass cation [57]. It was earlier shown that the relative position between the anion and cation comprising the bulk ionic liquid is one of the most important structural factors that determine the physical and chemical properties of a particular ionic liquid and that the anions usually are employed to tune the physicochemical properties of ILs [58,59]. Our results, however, align with other earlier work indicating that, although the cation plays a dominant role in antifungal activity, the anion also contributes significantly [55,60,61].

As noted before, **3r** containing quinoline moiety displayed the highest antifungal activity. In contrast, **3n**, containing only a pyridine moiety, showed no significant antifungal properties. This suggests that the quinoline substituent and the pyridinium-fused 1,3-selenazole scaffold act synergistically, producing a mycelial growth inhibition greater than either component alone. Indeed, the quinoline molecule on its own was not shown to inhibit any fungal growth, in contrast to 2-ethynylquinoline, which was used in the synthesis of 3**r** (Table 2). The latter is supported by the recently designed highly potent series of iodiconazole-based triazole compounds containing ethynyl groups [62].

The quinoline scaffolds have been privileged for their numerous biological activities in the pharmaceutical and agrochemical field within the last decade [63,64]. As far as antifungal application is concerned, along with 1,2,3-triazole appended quinolines, sulfur-containing pharmacores such as sulfonyl, thiol, and thiolone are attributed to noteworthy fungicidal properties [65].

## 3. Materials and Methods

**General remarks**. No uncommon hazards are noted derived from the experimental work carried out. All manipulations were carried out in air. All the reagents used in this study were obtained from the commercial sources (Aldrich, TCI-Europe, Strem, ABCR). Commercially available solvents were purified by conventional methods and distilled immediately prior to use. NMR spectra were recorded on a Bruker Avance neo 700 (^1^H: 700 MHz, ^13^C: 176 MHz, Karlsruhe, Germany); chemical shifts (*δ*) are given in ppm and coupling constants (*J*) in Hz.

### 3.1. Computational Methods

The geometries and energies of all systems included in the mechanistic study (Scheme 3) were fully optimized at the BP86-D4/def2-TZVP level of theory using the program ORCA version 5.0.4 [66]. For the calculations, we used the BP86 GGA functional [67,68] with D4 correction for the dispersion [69] and the def2-TZVP basis set [70]. Solvation effects were accounted for using the Conductor-like Polarizable Continuum Model (CPCM) with methanol or dichloromethane as the solvent [71]. The minimum and transition state nature of the complexes and compounds have been confirmed by performing frequency calculations. To confirm that each transition state correctly connects its corresponding minima, intrinsic reaction coordinate (IRC) calculations were carried out. The IRC paths were traced in both forward and reverse directions from the transition state geometry, leading to the respective product and pre-reaction complex, thus validating the reaction pathway. This computational approach has been validated in prior work on related reactions, demonstrating its suitability by striking an effective balance between accuracy and computational demands [39,40,43,45,48,49,50].

For the calculations, we used the BP86 GGA functional [67,68] with D4 correction for the dispersion [69] and the def2-TZVP basis set [70]. Solvation effects were accounted for using the Conductor-like Polarizable Continuum Model (CPCM) with methanol or dichloromethane as the solvent [71]. The minimum and transition state nature of the complexes and compounds have been confirmed by performing frequency calculations.

The single-point calculations using the X-ray coordinates of compounds **3d**, **2p**, and **3q** were performed using the Turbomole 7.7 program [72]. The level of theory used was the same as that used for the mechanistic study (BP86-D4/def2-TZVP). The Molecular Electrostatic Potential (MEP) surfaces were generated at the same level of theory on the 0.001 a.u. isosurface. The Quantum Theory of Atoms in Molecules (QTAIM) [73] distribution of bond critical points (CPs) and bond paths, as well as Non-Covalent Interaction (NCIplot) reduced density gradient (RDG) isosurfaces [74], were computed using the MultiWFN program [75] and plotted using the VMD program [76]. The following settings were used for the RDG plots: s = 0.5 a.u.; cut-off ρ = 0.04 a.u.; and color scale −0.035 a.u. ≤ sign(λ_2_)ρ ≤ 0.035 a.u. The Natural Bond Orbital (NBO) analysis [77] was performed at the same level using the NBO 7.0 program. The energies of the intermolecular HB interactions were estimated using Espinosa’s equation (E_HB_ = 0.5 × V(r)), while the strength of the ChB interactions was evaluated using the predictor proposed by Bauzá and Frontera for period 4 elements (E_ChB_ = 0.375 × V(r) − 0.57) [78].

### 3.2. Bioassay of Fungicidal Activities

The effect of the chemicals on mycelial radial growth was determined by dissolving compounds **a**, **c**, **g**, **h**, **i**, **j**, **k**, **m**, **o** and **s** in acetonitrile and compounds **b**, **l**, **n**, **r**, **u** and **v** in methanol. The compounds were dissolved in the respective solvents at a concentration of 3 mg/mL. Then aliquots were suspended in potato-saccharose agar at 50 °C to give the concentration 30 µg/mL. Petri dishes containing 15 mL of the agar medium were inoculated by placing 2 mm mycelial agar discs on the agar surface. Plates were incubated at 25 °C and radial growth was measured after 72 h. The agar medium without sample was used as the blank control. Three replicates of each test were carried out. The mycelium elongation diameter (mm) of fungi settlements was measured after 72 h of culture. The growth inhibition rates (I) were calculated using the following equation:

$$I=\frac{\mathrm{Control}\;\mathrm{settlement}\;\mathrm{diameter}\left(mm\right)-\mathrm{Test}\;\mathrm{settlement}\;\mathrm{diameter}\left(mm\right)}{\mathrm{Control}\;\mathrm{settlement}\;\mathrm{diameter}\left(mm\right)}\times 100\%$$

### 3.3. Synthesis of ***3a***–***3w***

The use of sodium perrhenate and sodium tetrachloroaurate for the precipitation of the cationic products was motivated primarily by crystallographic considerations. The presence of heavy atoms in the counterions significantly enhances the electron density of the crystalline salts, thereby enabling the collection of high-resolution X-ray diffraction data for unambiguous structural confirmation.

**Method A**. A solution of alkyne (1 equiv.) in MeOH (2 mL) was added to a solution of 2-pyridylselenyl chloride (1 equiv.) in MeOH (2 mL), and the reaction mixture was heated at 65 °C for 3 h. After that, the saturated MeOH solution of NaBPh_4_ (50 µL) was added to the reaction mixture leading to the formation of precipitate, which was filtered, washed with MeOH (3 × 1 mL) and Et_2_O (3 × 1 mL) and dried under vacuum.

**Method B**. A solution of NH_4_ReO_4_ (1 equiv.) in MeOH (1.5 mL) was added to a solution of [1,3]selenazolo [3,2-a]pyridin-4-ium chloride (1 equiv.) in MeOH (1.5 mL), and the reaction was left at 20 °C for 24 h. The solvent was evaporated under reduced pressure, the formed solid washed with Et_2_O (3 × 1 mL) and dried under vacuum.


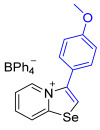


**3a** was prepared according to Method A. 2-Pyridylselenyl chloride (20 mg, 104 µmol) and 1-ethynyl-4-methoxybenzene (14 mg, 104 μmol) were used. Colorless precipitate. Yield: 37 mg (59%). ^1^H NMR (700 MHz, CD_2_Cl_2_) *δ* 8.50 (d, *J* = 6.8 Hz, 1H, H-5), 7.80 (s, 1H, H-2), 7.78 (d, *J* = 8.6 Hz, 1H, H-8), 7.69 (t, *J* = 7.9 Hz, 1H, H-7), 7.39 (t, *J* = 7.0 Hz, 1H, H-6), 7.36–7.33 (m, 8H, *o*-C_BPh4_), 7.29 (d, *J* = 8.7 Hz, 2H, H-2’,6’ of Ar), 7.12 (d, *J* = 8.7 Hz, 2H, H-3’,5’ of Ar), 6.97 (t, *J* = 7.4 Hz, 8H, *m*-C_BPh4_), 6.82 (t, *J* = 7.2 Hz, 4H, *p*-C_BPh4_), 3.91 (s, 3H, OMe). ^13^C NMR (176 MHz, CD_2_Cl_2_) *δ* 164.6 (q, *J* = 49.3 Hz, *ipso*-C_BPh4_), 162.8 (*C*-OCH_3_), 158.7 (N*C*Se), 142.5 (C3), 136.6 (*o*-C_BPh4_), 136.1 (C5), 135.4 (C7), 132.1 (*o*-C_6_H_4_OMe), 128.1 (C6), 126.3 (*m*-C_BPh4_), 125.6 (C8), 123.0 (C2), 122.5 (*p*-C_BPh4_), 119.0 (*ipso-C*-C_6_H_4_OMe), 116.1 (*m*-C_6_H_4_OMe), 56.3 (OCH_3_). IR (*ν*, cm^−1^): 3056 (w, arom. C-H), 1608 (m, C=N/C=C), 1580 (m), 1501 (m), 1478 (m), 1446 (m), 1306 (m), 1253 (s, C-O-C), 1176 (m), 1110 (w), 1067 (w), 1030 (m), 734 (vs, BPh_4_), 704 (vs, BPh_4_), 611 (s), 466 (w). MS (ESI^+^), found: 290.0084 [M − BPh_4_]^+^; calcd for C_14_H_12_NOSe: 290.0079.


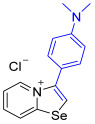


**3b**. A solution of 4-ethynyl-N,N-dimethylaniline (26.4 mg, 182 μmol) in CH_2_Cl_2_ (2 mL) was added to a suspension of 2-pyridylselenyl chloride (35 mg, 182 µmol) in CH_2_Cl_2_ (2 mL), and the reaction mixture was stirred at 20 °C for 48 h. The solvent was evaporated under reduced pressure, and the residue was washed with Et_2_O (3 × 1 mL) until a yellow solid was formed and dried under vacuum. Yield: 52 mg (84%). ^1^H NMR (700 MHz, CDCl_3_) *δ* 9.65 (d, *J* = 8.6 Hz, 1H, H-8), 8.81 (s, 1H, H-2), 8.77 (d, *J* = 6.7 Hz, 1H, H-5), 8.02 (t, *J* = 7.7 Hz, 1H, H-7), 7.65 (t, *J* = 6.8 Hz, 1H, H-6), 7.33 (d, *J* = 8.5 Hz, 2H, H-2’,6’ of Ar), 6.83 (d, *J* = 8.5 Hz, 2H, H-3’,5’ of Ar), 3.07 (s, 6H, NMe_2_). ^13^C NMR (176 MHz, CDCl_3_) *δ* 161.0 (NCSe), 151.9 (*C*-NMe_2_), 141.6 (C3), 134.0 (C5), 133.7 (C7), 131.1 (*o*-C_6_H_4_NMe_2_), 130.6 (C6), 130.1 (C8), 121.6 (C2), 114.0 (*ipso*-C_6_H_4_NMe_2_), 112.6 (*m*-C_6_H_4_NMe_2_), 40.3 (NMe_2_). IR (*ν*, cm^−1^): 3023 (w, arom. C-H), 2910 (w, aliph. C-H), 1610 (s, C=N/C=C), 1518 (m), 1448 (m), 1375 (m), 1282 (m), 1200 (m), 1066 (w), 954 (w), 827 (m), 781 (s), 720 (m), 615 (m), 554 (m), 517 (m). MS (ESI^+^), found: 303.0400 [M − Cl]^+^; calcd for C_15_H_15_N_2_Se: 303.0395.


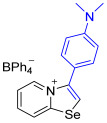


**3c** was prepared according to Method A. 2-Pyridylselenyl chloride (20 mg, 104 µmol) and 4-ethynyl-N,N-dimethylaniline (15 mg, 104 μmol) were used. Orange precipitate. Yield: 35 mg (54%). ^1^H NMR (700 MHz, CD_2_Cl_2_) *δ* 8.63 (d, *J* = 6.8 Hz, 1H, H-5), 7.81 (s, 1H, H-2), 7.77 (d, *J* = 8.5 Hz, 1H, H-8), 7.66 (t, *J* = 7.9 Hz, 1H, H-7), 7.38–7.33 (m, 9H, *o*-C_BPh4_), 7.21 (d, *J* = 8.4 Hz, 2H, H-2’,6’ of Ar), 6.98 (t, *J* = 7.3 Hz, 8H, *m*-C_BPh4_), 6.86–6.78 (m, 6H, H-3’,5’ of Ar, *p*-C_BPh4_), 3.07 (s, 6H, NMe_2_). ^13^C NMR (176 MHz, CD_2_Cl_2_) *δ* 164.7 (q, *J* = 49.3 Hz, *ipso*-C_BPh4_), 158.5 (NCSe), 152.8 (C-NMe_2_), 143.9 (C3), 136.5 (*o*-C_BPh4_), 136.0 (C5), 135.6 (C7), 131.4 (*o*-C_6_H_4_NMe_2_), 128.0 (C6), 126.3 (*m*-C_BPh4_), 124.0 (C2), 122.8 (C4), 122.4 (*p*-C_BPh4_), 114.5 (*ipso*-C_6_H_4_NMe_2_), 112.9 (*m*-C_6_H_4_NMe_2_), 40.5 (NMe_2_). IR (*ν*, cm^−1^): 3123 (w, arom. C-H), 3051 (w), 3032 (w), 2997 (w, aliph. C-H), 2888 (w), 1605 (m, C=N/C=C), 1583 (m), 1515 (m), 1480 (m), 1443 (m), 1424 (m), 1362 (m), 1182 (w), 1150 (w), 1065 (w), 1030 (w), 944 (w), 819 (m), 734 (vs, BPh_4_), 701 (vs, BPh_4_). MS (ESI^+^), found: 303.0400 [M − BPh_4_]^+^; calcd for C_15_H_15_N_2_Se: 303.0395.


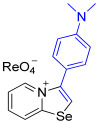


**3d** was prepared according to Method B. 3-(4-(Dimethylamino)phenyl)-[1,3]selenazolo [3,2-a]pyridin-4-ium chloride (11.4 mg, 34 µmol) and NH_4_ReO_4_ (9.1 mg, 34 μmol) were used. Yellow solid. Yield: 16 mg (86%). 9.18 (d, *J* = 6.8 Hz, 1H, H-5), 9.00 (d, *J* = 8.6 Hz, 1H, H-8), 8.66 (s, 1H, H-2), 8.37 (t, *J* = 7.9 Hz, 1H, H-7), 8.03 (t, *J* = 7.6 Hz, 1H, H-6), 7.54 (d, *J* = 8.9 Hz, 2H, *o*-C_6_H_4_NMe_2_), 6.97 (d, *J* = 8.9 Hz, 2H, *m*-C_6_H_4_NMe_2_), 3.10 (s, 6H, NMe_2_). ^13^C NMR (176 MHz, C_3_D_6_O) *δ* 160.0 (NCSe), 153.1 (C-NMe_2_), 144.6 (C3), 137.4 (C5), 136.8 (C7), 132.1 (C6), 128.5 (C2), 125.1 (C8), 123.5 (*o*-C_6_H_4_NMe_2_), 115.1 (*ipso*-C_6_H_4_NMe_2_), 113.2 (*m*-C_6_H_4_NMe_2_), 40.2 (NMe_2_). IR (*ν*, cm^−1^): 3112 (w, arom. C-H), 3060 (w), 2916 (w, aliph. C-H), 1604 (m, C=N/C=C), 1445 (m), 1366 (m, arom. C-N), 1184 (w), 1121 (w), 900 (vs, ReO_4_), 815 (m), 765 (s), 718 (m). MS (ESI^+^), found: 303.0399 [M − ReO_4_]^+^; calcd for C_15_H_15_N_2_Se: 303.0395.


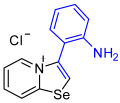


**3e**. A solution of 2-ethynylaniline (12.7 mg, 108 μmol) in CH_2_Cl_2_ (2 mL) was added to a suspension of 2-pyridylselenyl chloride (20.8 mg, 108 µmol) in CH_2_Cl_2_ (2 mL), and the reaction mixture was stirred at 20 °C for 48 h. Subsequently, formed pale yellow precipitate was separated, and the solid was washed with Et_2_O (3 × 1 mL) and dried under vacuum. Yield: 25 mg (75%). ^1^H NMR (700 MHz, D_2_O) *δ* 8.77 (s, 1H, H-2), 8.74 (d, *J* = 8.6 Hz, 1H, H-8), 8.67 (d, *J* = 6.8 Hz, 1H, H-5), 8.22 (t, *J* = 8.0 Hz, 1H, H-7), 7.83 (t, *J* = 7.0 Hz, 1H, H-6), 7.64–7.61 (m, 1H, H-4’), 7.44 (d, *J* = 9.1 Hz, 1H, H-6’), 7.25 (d, *J* = 8.3 Hz, 1H, H-3’), 7.23 (d, *J* = 8.3 Hz, 1H, H-5’). ^13^C NMR (176 MHz, D_2_O) *δ* 158.7 (NCSe), 145.5 (C-NH_2_), 139.6 (C3), 136.2 (C5), 135.9 (C7), 132.9 (C6), 132.2 (C6’), 127.2 (C8), 126.8 (C4’), 122.4 (*ipso*-C_6_H_4_NMe_2_), 119.9 (C2), 117.6 (C5’), 113.4 (C3’). IR (*ν*, cm^−1^): 3314 (m, br, N-H stretch), 3022 (w, arom. C-H), 2976 (w), 2776 (w), 1647 (m, N-H bend), 1615 (s, C=N/C=C), 1585 (m), 1560 (m), 1534 (m), 1489 (m), 1449 (vs), 1282 (m), 1152 (m), 1117 (m), 1070 (m), 715 (s), 628 (m), 541 (m), 507 (m), 458 (s). MS (ESI^+^), found: 275.0088 [M − Cl]^+^; calcd for C_13_H_11_N_2_Se: 275.0082.


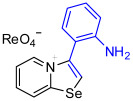


**3f** was prepared according to Method B. 3-(2-Aminophenyl)-[1,3]selenazolo [3,2-a]pyridin-4-ium chloride (15 mg, 48 µmol) and NH_4_ReO_4_ (13 mg, 48 μmol) were used. Yellow solid. Yield: 21 mg (83%). ^1^H NMR (700 MHz, DMSO-d_6_) *δ* 9.00 (d, *J* = 8.6 Hz, 1H, H-8), 8.72 (s, 1H, H-2), 8.45 (d, *J* = 6.7 Hz, 1H, H-5), 8.24 (t, *J* = 8.2 Hz, 1H, H-7), 7.88 (t, *J* = 7.5 Hz, 1H, H-6), 7.37 (t, *J* = 8.3 Hz, 1H, H-4’), 7.26 (d, *J* = 7.4 Hz, 1H, H-6’), 6.94 (d, *J* = 8.2 Hz, 1H, H-3’), 6.79 (t, *J* = 7.3 Hz, 1H, H-5’). ^13^C NMR (176 MHz, DMSO-d_6_) *δ* 159.6 (NCSe), 146.7 (C-2’, C-NH_2_), 139.9 (C3), 136.4 (C-7), 135.2 (C-5), 132.5 (C-4’), 132.1 (C-6’), 129.0 (C-3), 127.5 (C-6), 122.2 (C-8), 116.9 (C-5’), 116.1 (C-1’, quaternary Ar), 111.7 (C-3’). IR (*ν*, cm^−1^): 3395 (w, N-H), 3326 (w, N-H), 3267 (w), 1634 (m, C=N), 1448 (m), 1344 (m), 1046 (m), 891 (vs, ReO_4_), 886 (vs, ReO_4_), 771 (s, *γ* C-H), 744 (s, *γ* C-H). MS (ESI^+^), found: 275.0087 [M − ReO_4_]^+^; calcd for C_13_H_11_N_2_Se: 275.0082.


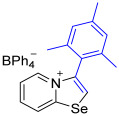


**3g** was prepared according to Method A. 2-Pyridylselenyl chloride (30 mg, 156 µmol) and 2-ethynyl-1,3,5-trimethylbenzene (22 mg, 156 μmol) were used. Colorless precipitate. Yield: 50 mg (52%). ^1^H NMR (700 MHz, CD_2_Cl_2_) *δ* 8.00 (d, *J* = 6.7 Hz, 1H, H-5), 7.85 (s, 1H, H-2), 7.67 (d, *J* = 8.6 Hz, 1H, H-8), 7.62 (t, *J* = 7.9 Hz, 1H, H-7), 7.40–7.38 (m, 8H, *o*-H_BPh4_), 7.36 (t, *J* = 6.9 Hz, 1H, H-6), 7.13 (s, 2H, m-CH, Mes), 6.99 (t, *J* = 7.4 Hz, 8H, *m*-H_BPh4_), 6.83 (t, *J* = 7.2 Hz, 4H, *p*-H_BPh4_), 2.42 (s, 3H, *p*-Me, Mes), 1.89 (s, 6H, *o*-Me, Mes). ^13^C NMR (176 MHz, CD_2_Cl_2_) *δ* 164.8 (q, *J* = 49.3 Hz, *ipso*-C_BPh4_), 158.7 (NCSe), 143.1 (Mesityl C4), 141.1 (C3), 139.3 (Mesityl C2,6), 136.6 (*o*-C_BPh4_), 136.2 (C5), 134.6 (C7), 130.2 (Mesityl C3,5), 128.3 (Mesityl C1), 126.4 (*m*-C_BPh4_), 126.2 (C2), 123.4 (C8), 123.1 (*p*-C_BPh4_), 122.5 (C6), 21.7 (Mesityl p-CH_3_), 19.9 (Mesityl *o*-CH_3_). IR (*ν*, cm^−1^): 3086 (w, arom. C-H), 3005 (w, aliph. C-H), 2983 (w), 1611 (m, C=N/C=C), 1577 (m), 1477 (m), 1424 (m), 1379 (w, methyl *δ*(CH_3_), 1279 (m), 1149 (w), 1109 (w), 1032 (m), 847 (m, mesityl ring C-H), 733 (vs, BPh_4_), 701 (vs, BPh_4_), 611 (s). MS (ESI^+^), found: 302.0447 [M − BPh_4_]^+^; calcd for C_16_H_16_NSe: 302.0442.


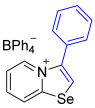


**3h** was prepared according to Method A. 2-Pyridylselenyl chloride (25 mg, 130 µmol) and phenylacetylene (13 mg, 130 μmol) were used. Colorless precipitate. Yield: 41 mg (55%). ^1^H NMR (700 MHz, CD_3_CN) *δ* 8.78 (d, *J* = 6.8 Hz, 1H, H-5), 8.61 (d, *J* = 8.6 Hz, 1H, H-8), 8.48 (s, 1H, H-2), 8.12 (t, *J* = 7.9 Hz, 1H, H-7), 7.75–7.71 (m, 2H, H-6, *p*-CH of Ph), 7.70 (t, *J* = 7.4 Hz, 2H, *m*-CH of Ph), 7.61 (d, *J* = 6.9 Hz, 2H, *o*-CH of Ph), 7.32–7.29 (m, 8H,), 7.02 (t, *J* = 7.5 Hz, 8H, *m*-H_BPh4_), 6.87 (t, *J* = 7.2 Hz, 4H, *p*-H_BPh4_). ^13^C NMR (176 MHz, CD_3_CN) *δ* 164.9 (q, *J* = 49.3 Hz, *ipso*-C of BPh_4_), 159.7 (NCSe), 143.2 (C-3), 137.2 (C-5), 137.0 (*o*-C of BPh_4_), 136.7 (C-7), 132.3 (C-2), 131.3 (*ipso*-C of Ph), 130.7 (*o*-C of Ph), 128.9 (C-6), 128.3 (*m*-C of Ph), 126.5 (C-8), 126.4 (*m*-C of BPh_4_), 123.5 (*p*-C of Ph), 122.7 (*p*-C of BPh_4_).IR (*ν*, cm^−1^): 3092 (w, arom. C-H), 3053 (w), 2998 (w), 1618 (m, C=N/C=C), 1578 (m), 1477 (m), 1445 (s), 1302 (w), 1281 (m), 1152 (m), 1117 (m), 1069 (m), 1033 (m), 733 (vs, BPh_4_), 703 (vs, BPh_4_). MS (ESI^+^), found: 259.9978 [M − BPh_4_]^+^; calcd for C_13_H_10_NSe: 259.9973.


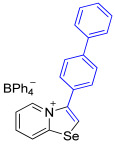


**3i** was prepared according to Method A. 2-Pyridylselenyl chloride (20 mg, 104 µmol) and 4-ethynyl-1,1’-biphenyl (18 mg, 104 μmol) were used. Yellow precipitate. Yield: 36 mg (53%). ^1^H NMR (700 MHz, C_3_D_6_O) *δ* 9.18 (d, *J* = 6.8 Hz, 1H, H-5), 8.97 (d, *J* = 8.6 Hz, 1H, H-8), 8.85 (s, 1H, H-2), 8.34 (t, *J* = 7.9 Hz, 1H, H-7), 8.00–7.97 (m, 3H, H-6 and H of biphenyl), 7.84 (d, *J* = 8.3 Hz, 2H, H of biphenyl), 7.78 (d, *J* = 7.2 Hz, 2H, *o*-CH of Ph), 7.54 (t, *J* = 7.7 Hz, 2H, *m*-CH of Ph), 7.46 (t, *J* = 7.4 Hz, 1H, *p*-CH of Ph), 7.33–7.30 (m, 8H, *o*-H_BPh4_), 6.90 (t, *J* = 7.4 Hz, 8H, *m*-H_BPh4_), 6.75 (t, *J* = 7.2 Hz, 4H, *p*-H_BPh4_). ^13^C NMR (176 MHz, C_3_D_6_O) *δ* 165.1 (q, *J* = 49.3 Hz, *ipso*-C of BPh_4_), 160.2 (C-1), 144.6 (C-3), 143.2 (biphenyl CH), 140.4 (biphenyl CH), 137.5 (C-5), 137.0 (C-7 and *o*-C of BPh_4_), 132.0 (C-2), 130.1 (biphenyl CH), 129.9, 129.2, 129.0, 128.5, 128.1, 127.9, 127.0 (biphenyl CH), 126.0 (*m*-C of BPh_4_), 123.7 (C-8), 122.2 (*p*-C of BPh_4_). IR (*ν*, cm^−1^): 3089 (w, arom. C-H), 3055 (w), 2998 (w), 1610 (m, C=N/C=C), 1577 (m), 1477 (m), 1425 (m), 1283 (m), 1115 (m), 1072 (w), 1033 (m), 731 (vs, BPh_4_), 704 (vs, BPh_4_). MS (ESI^+^), found: 336.0292 [M − BPh_4_]^+^; calcd for C_19_H_14_NSe: 336.0286.


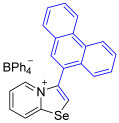


**3j** prepared according to Method A. 2-Pyridylselenyl chloride (30.5 mg, 158 µmol) and 9-ethynylphenanthrene (32 mg, 158 μmol) were used. Colorless precipitate. Yield: 55 mg (51%). ^1^H NMR (700 MHz, C_3_D_6_O) *δ* 9.05 (d, *J* = 8.3 Hz, 2H, phenanthryl), 9.01 (d, *J* = 8.4 Hz, 1H, phenanthryl), 8.98 (d, *J* = 8.7 Hz, 1H, H-8), 8.77 (d, *J* = 6.8 Hz, 1H, H-5), 8.28 (t, *J* = 7.9 Hz, 1H, H-7), 8.24 (s, 1H, H-2), 8.10 (d, *J* = 7.7 Hz, 1H, phenanthryl), 7.92 (t, *J* = 8.3 Hz, 1H, phenanthryl), 7.83 (t, *J* = 7.6 Hz, 1H, H-6), 7.80 (q, *J* = 7.0 Hz, 2H, phenanthryl), 7.59 (t, *J* = 8.0 Hz, 1H, phenanthryl), 7.54 (d, *J* = 7.3 Hz, 1H, phenanthryl), 7.36–7.32 (m, 8H, *o*-H_BPh4_), 6.90 (t, *J* = 7.5 Hz, 8H, *m*-H_BPh4_), 6.76 (t, *J* = 7.2 Hz, 4H, *m*-H_BPh4_). 165.1 (q, *J* = 49.5 Hz, *ipso*-C of BPh_4_), 160.3 (C-1), 157.7 (phenanthrene CH), 141.7 (C-3), 137.8 (C-5), 137.1 (C-7), 137.0 (*o*-C of BPh_4_), 134.2, 132.5, 131.8, 131.8, 130.6, 130.5, 130.0, 129.0, 128.9, 128.7, 128.6 (phenanthrene CH), 126.0 (*m*-C of BPh_4_), 125.0, 124.7, 124.0 (phenanthrene CH and C-2), 123.8 (C-8), 122.3 (*p*-C of BPh_4_). IR (*ν*, cm^−1^): 3054 (w, arom. C-H), 1614 (m, C=N/C=C), 1578 (w), 1478 (m), 1449 (m), 1428 (m), 1265 (w), 1149 (w), 1031 (w), 843 (w), 734 (s, *γ* C-H), 707 (vs, BPh_4_). MS (ESI^+^), found: 360.0292 [M − BPh_4_]^+^; calcd for C_21_H_14_NSe: 360.0286.


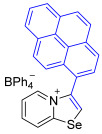


**3k** prepared according to Method A. 2-Pyridylselenyl chloride (24.2 mg, 126 µmol) and 1-ethynylpyrene (28.4 mg, 126 μmol) were used. Yellow precipitate. Yield: 48 mg (54%). ^1^H NMR (700 MHz, C_3_D_6_O) *δ* 9.08 (s, 1H, H-2), 9.00 (d, J = 8.7 Hz, 1H, H-8), 8.73 (d, J = 6.8 Hz, 1H, H-5), 8.54 (d, J = 7.8 Hz, 1H, pyrenyl), 8.49 (d, J = 7.5 Hz, 1H, pyrenyl), 8.42 (t, J = 9.4 Hz, 2H, pyrenyl and H-7), 8.36 (d, J = 9.0 Hz, 1H, pyrenyl), 8.31 (d, J = 7.7 Hz, 1H, pyrenyl), 8.29 (d, J = 8.5 Hz, 1H, pyrenyl), 8.23 (d, J = 9.2 Hz, 1H, pyrenyl), 8.21 (t, J = 7.6 Hz, 1H, pyrenyl), 7.83 (d, J = 9.1 Hz, 1H, pyrenyl), 7.80 (t, J = 7.0 Hz, 1H, H-6), 7.35–7.33 (m, 8H, *o*-H_BPh4_), 6.90 (t, J = 7.4 Hz, 8H, *m*-H_BPh4_), 6.75 (t, J = 7.2 Hz, 4H, *p*-H_BPh4_). ^13^C NMR (176 MHz, C_3_D_6_O) 165.1 (q, *J* = 49.5 Hz, *ipso*-C of BPh_4_), 160.4 (C-1), 141.9 (C-3), 137.8 (C-5), 137.1 (C-7), 137.0 (*o*-C of BPh_4_), 134.5, 132.1, 131.7, 131.6, 130.8, 130.5, 130.4, 129.0, 128.7, 128.2, 128.0, 127.7, 127.5, 126.4 (pyrenyl CH), 126.0 (*m*-C of BPh_4_), 125.6, 124.9, 124.1 (pyrenyl CH), 123.8 (C-8), 122.5 (C-2), 122.3 (*p*-C of BPh_4_). IR (*ν*, cm^−1^): 3052 (w, arom. C-H), 2986 (w), 1610 (w, C=N/C=C), 1580 (m), 1475 (m), 1271 (m), 1242 (m), 1153 (m), 1122 (m), 1070 (m), 1032 (m), 853 (s, pyrenyl *γ*(C-H)), 764 (m), 734 (vs, BPh_4_), 697 (vs, BPh_4_). MS (ESI^+^), found: 384.0291 [M − BPh_4_]^+^; calcd for C_23_H_14_NSe: 384.0286.


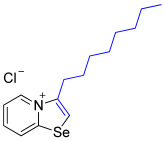


**3l**. A solution of 1-decin (11.6 mg, 84 μmol) in 1,2-DCE (2 mL) was added to a suspension of 2-pyridylselenyl chloride (16.2 mg, 84 µmol) in 1,2-DCE (2 mL), and the reaction mixture was heated at 84 °C for 3 h. The solvent was evaporated under reduced pressure and the formed dark brown oil washed with Et_2_O (5 × 1 mL) and dried under vacuum. Yield: 21 mg (76%). ^1^H NMR (700 MHz, D_2_O) *δ* 9.13 (d, *J* = 6.8 Hz, 1H, H-5), 8.64 (d, *J* = 8.6 Hz, 1H, H-8), 8.34 (s, 1H, H-2), 8.15 (t, *J* = 7.9 Hz, 1H, H-7), 7.90 (t, *J* = 7.0 Hz, 1H, H-6), 3.06 (t, *J* = 7.5 Hz, 2H, H-1’ of octyl), 1.84 (p, *J* = 7.6 Hz, 2H, H-2’ of octyl), 1.45 (p, *J* = 7.6 Hz, 2H, H-3’ of octyl), 1.37–1.33 (m, 2H, H-4’ of octyl), 1.31–1.23 (m, 6H, H-5’, H-6’, H-7’ of octyl), 0.83 (t, *J* = 6.9 Hz, 3H, Me). ^13^C NMR (176 MHz, D_2_O) *δ* 158.9 (C-1), 142.9 (C-3), 135.2 (C-5), 135.1 (C-7), 127.1 (C-6), 122.2 (C-2), 121.6 (C-8), 31.1 (C-1′), 28.3 (C-2′), 28.3 (C-3′), 28.2 (C-4′), 28.0 (C-5′), 25.1 (C-6′), 22.0 (C-7′), 13.4 (C-8′). IR (*ν*, cm^−1^): 2925 (m, ν C–H aliph), 2854 (m, ν C–H aliph), 1602 (m, ν C=N/C=C_arom), 1548 (m, ν C=C), 1466 (m, δ C–H), 1378 (m, δ C–H), 1312 (m, ν C–N), 1248 (m), 1184 (m), 1042 (m), 832 (m, *γ* C–H arom), 756 (s, *γ* C–H). MS (ESI^+^), found: 296.0917 [M − Cl]^+^; calcd for C_15_H_22_NSe: 296.0912.


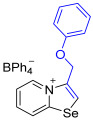


**3m** prepared according to Method A. 2-Pyridylselenyl chloride (28 mg, 147 µmol) and (prop-2-yn-1-yloxy)benzene (19 mg, 147 μmol) were used. Colorless precipitate. Yield: 49 mg (55%). ^1^H NMR (700 MHz, C_3_D_6_O) *δ* 9.48 (d, *J* = 6.8 Hz, 1H, H-5), 9.06 (s, 1H, H-2), 8.89 (d, *J* = 8.6 Hz, 1H, H-8), 8.34–8.31 (m, 1H, H-7), 8.08 (t, *J* = 7.6 Hz, 1H, H-6), 7.38–7.33 (m, 10H, *m*-H of OPh, *o*-H_BPh4_), 7.12 (d, *J* = 9.5 Hz, 2H, *o*-H of OPh), 7.06 (t, *J* = 7.4 Hz, 1H, *p*-H of OPh), 6.91 (t, *J* = 7.4 Hz, 8H, *m*-H_BPh4_), 6.76 (t, *J* = 7.2 Hz, 4H, *p*-H_BPh4_), 5.69 (s, 2H, OCH_2_). ^13^C NMR (176 MHz, C_3_D_6_O) *δ* 165.1 (q, *J* = 49.5 Hz, *ipso*-C of BPh_4_), 160.6 (C-1), 158.4 (phenyl CH), 138.8 (C-3), 137.7 (C-5), 137.3 (C-7), 137.0 (*o*-C of BPh_4_), 130.6 (phenyl CH), 129.9 (phenyl CH), 128.7 (phenyl CH), 126.0 (*m*-C of BPh_4_), 123.9 (C-8), 123.1 (C-2), 122.3 (*p*-C of BPh_4_), 116.0 (*o*-C of OC_6_H_5_), 63.3 (OCH_2_). IR (*ν*, cm^−1^): 3082 (w, arom. C-H), 3055 (w), 3002 (w), 1597 (m, C=N/C=C), 1481 (m), 1425 (m), 1374 (w), 1288 (w), 1228 (s, *ν* C-O-C), 1149 (w), 1082 (w), 1035 (m), 996 (w), 843 (m), 808 (m), 734 (vs, BPh_4_), 707 (vs, BPh_4_). MS (ESI^+^), found: 290.0083 [M − BPh_4_]^+^; calcd for C_14_H_12_NOSe: 290.0079.


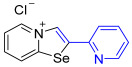


**3n**. A solution of 2-ethynylpyridine (16.5 mg, 160 μmol) in CH_2_Cl_2_ (2 mL) was added to a suspension of 2-pyridylselenyl chloride (30.8 mg, 160 µmol) in CH_2_Cl_2_ (2 mL), and the reaction mixture was stirred at 20 °C for 48 h. The solvent was evaporated under reduced pressure, and the residue was recrystallized from a mixture of MeOH/Et_2_O with the formation of dark brown powder, which was separated by decantation, washed with CH_2_Cl_2_ (3 × 1 mL) and Et_2_O (3 × 1 mL) and dried under vacuum. Yield: 21 mg (44%). ^1^H NMR (700 MHz, D_2_O) *δ* 9.22 (d, *J* = 6.6 Hz, 1H, H-5), 9.14 (s, 1H, H-3), 8.59 (d, *J* = 8.6 Hz, 1H, H-8), 8.57 (d, *J* = 4.9 Hz, 1H, H-6′), 8.13 (t, *J* = 7.4 Hz, 1H, H-7), 8.04 (d, *J* = 7.9 Hz, 1H, H-3′), 7.99 (td, *J* = 7.7, 1.6 Hz, 1H, H-4′), 7.89 (t, *J* = 7.0 Hz, 1H, H-6), 7.53–7.51 (m, 1H, H-5′). ^13^C NMR (176 MHz, D_2_O) *δ* 157.5 (C-1), 150.0 (C-2′), 147.6 (C-4′), 145.9 (C-3), 138.5 (C-5), 138.2 (C-5′), 136.5 (C-7), 127.2 (C-6), 126.9 (C-3′), 125.9 (C-4′), 122.9 (C-2), 120.7 (C-8). IR (*ν*, cm^−1^): 3086 (w, arom. C-H), 3053 (w), 3015 (w), 2922 (w), 1627 (m, C=N/C=C), 1587 (m), 1459 (s), 1437 (s), 1338 (m), 1226 (m), 1149 (m), 1076 (m), 1035 (m), 994 (m), 854 (m), 777 (vs, γ C-H pyridyl), 738 (m), 711 (m), 618 (m), 584 (m), 558 (m), 514 (s), 454 (s). MS (ESI^+^), found: 260.9931 [M − Cl]^+^; calcd for C_12_H_9_N_2_Se: 260.9925.


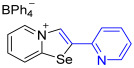


**3o**. 2-(Pyridin-2-yl)-[1,3]selenazolo [3,2-a]pyridin-4-ium chloride **3m** (14.5 mg, 49 μmol) was dissolved in MeOH (1.5 mL) and the addition of the MeOH solution of NaBPh_4_ (16.8 mg, 49 μmol) (1.5 mL) resulted in the formation of grey–green precipitate. The reaction was left at 20 °C for 24 h and a solution was decanted from the precipitate, which then was washed with MeOH (3 × 1 mL) and Et_2_O (3 × 1 mL) and dried under vacuum. Yield: 23 mg (81%). ^1^H NMR (700 MHz, CD_3_CN) *δ* 9.03 (d, *J* = 6.6 Hz, 2H, H-5), 8.96 (s, 1H, H-3), 8.69 (d, *J* = 4.8 Hz, 1H, H-6′), 8.57 (d, *J* = 8.6 Hz, 1H, H-8), 8.11 (t, *J* = 7.4 Hz, 1H, H-7), 8.0–8.02 (m, 2H, H-4′ and H-3’), 7.85 (t, *J* = 6.5 Hz, 1H, H-6), 7.57–7.54 (m, 1H, H-5′), 7.32–7.29 (m, 8H, *o*-H_BPh4_), 7.01 (t, *J* = 7.5 Hz, 8H, *m*-H_BPh4_), 6.86 (t, *J* = 7.2 Hz, 4H, *p*-H_BPh4_). ^13^C NMR (176 MHz, CD_3_CN) *δ* 164.9 (q, *J* = 49.5 Hz, *ipso*-C of BPh_4_), 151.4 (C-2′), 148.8 (C-4′), 148.4 (C-3), 139.4 (C-5), 139.1 (C-5′), 137.6 (C-7), 136.6 (*o*-C of BPh_4_), 128.1 (C-6), 127.5 (C-3′), 126.8 (C-4′), 126.5 (*m*-C of BPh_4_), 126.5 (C-2), 124.0 (C-8), 122.7 (*p*-C of BPh_4_), 120.9 (C-1). IR (*ν*, cm^−1^): 3078 (w, arom. C-H), 3050 (w), 2996 (w, aliph. C-H), 1580 (m, C=N/C=C), 1478 (m), 1427 (m), 1268 (w), 1148 (m), 1030 (m), 847 (m), 777 (s, γ C-H pyridyl), 733 (vs, BPh_4_), 703 (vs, BPh_4_). MS (ESI^+^), found: 260.9930 [M − BPh_4_]^+^; calcd for C_12_H_9_N_2_Se: 260.9925.


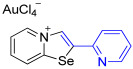


**3p**. 2-(Pyridin-2-yl)-[1,3]selenazolo [3,2-a]pyridin-4-ium chloride **3m** (9.9 mg, 33 μmol) was dissolved in MeOH (1.5 mL) and the addition of the MeOH solution of NaAuCl_4_·2H_2_O (13.3 mg, 33 μmol) (1.5 mL) resulted in the formation of yellow precipitate. The reaction was left at 20 °C for 24 h and a solution was decanted from the precipitate, which then was washed with Et_2_O (3 × 1 mL) and dried under vacuum. Yield: 13 mg (65%). ^1^H NMR (700 MHz, C_3_D_6_O) *δ* 9.64 (s, 1H, H-2), 9.58 (d, *J* = 6.6 Hz, 1H, H-5), 8.97 (d, *J* = 8.6 Hz, 1H, H-8), 8.75 (d, *J* = 5.1 Hz, 1H, H-6′), 8.39 (t, *J* = 7.4 Hz, 1H, H-7), 8.27 (d, *J* = 7.9 Hz, 1H, H-3′), 8.14 (t, *J* = 6.4 Hz, 1H, H-6), 8.12 (td, *J* = 7.7, 1.7 Hz, 1H, H-4′), 7.64–7.62 (m, 1H, H-5′). ^13^C NMR (176 MHz, C_3_D_6_O) *δ* 159.0 (C-1), 151.5 (C-2′), 149.1 (C-4′), 148.4 (C-3), 140.0 (C-5), 139.1 (C-5′), 137.8 (C-7), 128.4 (C-6), 128.3 (C-3′), 126.8 (C-4′), 124.2 (C-2), 120.9 (C-8). IR (*ν*, cm^−1^): 3105 (w, arom. C-H), 3068 (w), 3030 (w), 1618 (m, C=N/C=C), 1584 (m), 1532 (m), 1451 (s), 1429 (s), 1325 (m), 1256 (m), 1215 (m), 1163 (m), 1076 (m), 821 (m), 778 (s, γ C-H pyridyl), 735 (m), 707 (m), 685 (m), 615 (m), 521 (m). MS (ESI^+^), found: 260.9930 [M − AuCl_4_]^+^; calcd for C_12_H_9_N_2_Se: 260.9925.


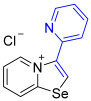


**3q**. A solution of 2-ethynylpyridine (16.5 mg, 160 μmol) in CH_2_Cl_2_ (2 mL) was added to a suspension of 2-pyridylselenyl chloride (30.8 mg, 160 µmol) in CH_2_Cl_2_ (2 mL), and the reaction mixture was stirred at 20 °C for 48 h. The solvent was evaporated under reduced pressure, and the residue was recrystallized from a mixture of MeOH/Et_2_O with the formation of brown powder **3m**, which was separated by decantation. Further, Et_2_O was added to the supernatant, resulting in the formation of a greyish-white powder. The solid was washed with Et_2_O (3 × 1 mL) and dried under vacuum. Yield: 10 mg (21%). ^1^H NMR (700 MHz, D_2_O) *δ* 9.39 (d, *J* = 6.9 Hz, 1H, H-5), 8.94 (s, 1H, H-2), 8.80 (d, *J* = 4.9 Hz, 1H, H-6′), 8.74 (d, *J* = 8.7 Hz, 1H, H-8), 8.23 (t, *J* = 8.0 Hz, 1H, H-7), 8.17 (t, *J* = 8.4 Hz, 1H, H-4′), 7.89 (d, *J* = 7.8 Hz, 1H, H-3′), 7.85 (t, *J* = 7.1 Hz, 1H, H-6), 7.74–7.71 (m, 1H, H-5′). ^13^C NMR (176 MHz, D_2_O) *δ* 159.1, (C-1), 149.7 (C-2′), 146.7 (C-3), 139.9 (C-5), 139.6 (C-5′), 136.6 (C-7), 136.3 (C-4′), 128.1 (C-6), 127.2 (C-3′), 126.9 (C-4′), 126.1 (C-2), 122.3 (C-8). IR (*ν*, cm^−1^): 3013 (w, arom. C-H), 1618 (m, C=N/C=C), 1586 (s), 1528 (m), 1454 (s), 1436 (s), 1322 (m), 1282 (m), 1252 (m), 1216 (m), 1163 (m), 1076 (m), 991 (m), 778 (s, γ C-H pyridyl), 765 (s), 735 (m), 710 (s), 685 (m), 615 (s), 519 (m).


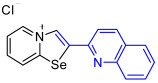


**3r**. A solution of 2-ethynylquinoline (36.2 mg, 236 μmol) in CH_2_Cl_2_ (2 mL) was added to a suspension of 2-pyridylselenyl chloride (45.5 mg, 236 µmol) in CH_2_Cl_2_ (2 mL), and the reaction mixture was stirred at 20 °C for 48 h. Subsequently, formed light brown precipitate was separated, and the solid was washed with CH_2_Cl_2_ (2 × 1 mL) and Et_2_O (3 × 1 mL) and dried under vacuum. Yield: 47 mg (58%). ^1^H NMR (700 MHz, D_2_O) *δ* 8.47 (d, *J* = 6.0 Hz, 1H, H-5), 8.29 (s, 1H, H-2), 8.03 (d, *J* = 8.2 Hz, 2H, H-8 and H-4′), 7.75 (t, *J* = 7.7 Hz, 1H, H-7), 7.48 (t, *J* = 6.6 Hz, 1H, H-6), 7.43–7.39 (m, 3H, H-3′, H-6′, and H-7′), 7.26–7.24 (m, 1H, H-5′), 7.15–7.13 (m, 1H, H-8′). ^13^C NMR (176 MHz, D_2_O) *δ* 156.6 (C-1), 147.1 (C-2′), 146.1 (C-3), 146.0 (C-8a′), 137.9 (C-5), 137.2 (C-7), 136.5 (C-4′), 130.6 (C-5′), 128.3 (C-6), 127.6 (C-6′), 127.4 (C-7′), 127.3 (C-4a′), 126.9 (C-3′), 126.3 (C-8), 122.8 (C-2), 116.5 (C-8′). IR (*ν*, cm^−1^): 3045 (w, arom. C-H), 2990 (w, aliph. C-H), 2923 (w), 1627 (m, C=N/C=C), 1593 (m), 1557 (m), 1501 (m), 1471 (s), 1345 (m), 1262 (m), 1221 (m), 1149 (m), 933 (m), 824 (s), 791 (s), 754 (vs, γ C-H quinolyl), 708 (s), 621 (m), 524 (m). MS (ESI^+^), found: 311.0087 [M − Cl]^+^; calcd for C_16_H_11_N_2_Se: 311.0082.


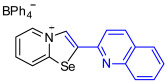


**3s**. 2-(Quinolin-2-yl)-[1,3]selenazolo [3,2-a]pyridin-4-ium chloride **3q** (14.9 mg, 43 μmol) was dissolved in MeOH (1.5 mL) and the addition of the MeOH solution of NaBPh_4_ (14.8 mg, 43 μmol) (1.5 mL) resulted in the formation of grey–green precipitate. The reaction was left at 20 °C for 24 h and a solution was decanted from the precipitate, which then was washed with MeOH (3 × 1 mL) and Et_2_O (3 × 1 mL) and dried under vacuum. Yield: 22 mg (82%). ^1^H NMR (700 MHz, C_3_D_6_O) *δ* 9.59 (s, 1H, H-2), 9.43 (d, *J* = 6.5 Hz, 1H, H-5), 8.91 (d, *J* = 8.6 Hz, 1H, H-8), 8.62 (d, *J* = 8.5 Hz, 1H, H-4′), 8.33 (t, *J* = 7.7 Hz, 1H, H-7), 8.25 (d, *J* = 8.5 Hz, 1H, H-3′), 8.14 (d, *J* = 8.4 Hz, 1H, H-8′), 8.09 (d, *J* = 8.0 Hz, 1H, H-5′), 8.06 (t, *J* = 6.8 Hz, 1H, H-6), 7.92 (t, *J* = 8.2 Hz, 1H, H-7′), 7.76 (t, *J* = 7.5 Hz, 1H, H-6′), 7.37–7.33 (m, 8H, *o*-H_BPh4_), 6.92 (t, *J* = 7.4 Hz, 8H, *m*-H_BPh4_), 6.76 (t, *J* = 7.2 Hz, 4H, *p*-H_BPh4_). ^13^C NMR (176 MHz, C_3_D_6_O) *δ* 165.1 (q, *J* = 49.4 Hz, *ipso*-C of BPh_4_), 149.3 (C-1), 148.7 (C-2′), 148.5 (C-3), 140.1 (C-5), 139.4 (C-7), 138.1 (C-8a′), 137.0 (*o*-C of BPh_4_), 132.1 (C-4′), 129.8 (C-5′), 129.6 (C-6′), 129.6 (C-7′), 129.4 (C-4a′), 129.1 (C-6), 128.4 (C-3′), 126.0 (*m*-C of BPh_4_), 126.0 (C-2), 124.2 (C-8), 122.3 (*p*-C of BPh_4_), 117.9 (C-8′). IR (*ν*, cm^−1^): 3098 (w, arom. C-H), 3056 (w), 3003 (w), 1621 (w, C=N/C=C), 1591 (m), 1501 (m), 1478 (m), 1449 (m), 1326 (m), 1259 (m), 1143 (m), 1122 (m), 1033 (m), 933 (m), 823 (m), 783 (m, γ C-H quinolyl), 734 (vs, BPh_4_), 703 (vs, BPh_4_). MS (ESI^+^), found: 311.0086 [M − BPh_4_]^+^; calcd for C_16_H_11_N_2_Se: 311.0082.


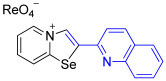


**3t** was prepared according to Method B. 2-(Quinolin-2-yl)-[1,3]selenazolo [3,2-a]pyridin-4-ium chloride (15 mg, 43 µmol) and NH_4_ReO_4_ (11.6 mg, 43 μmol) were used. Brown solid. Yield: 19 mg (78%). ^1^H NMR (700 MHz, DMSO-d_6_) *δ* 9.82 (s, 1H, H-2), 9.40 (d, *J* = 6.5 Hz, 1H, H-5), 8.89 (d, *J* = 8.6 Hz, 1H, H-8), 8.69 (d, *J* = 8.5 Hz, 1H, H-4′), 8.29 (t, *J* = 8.4 Hz, 2H, H-7 and H-3′), 8.11 (t, *J* = 7.1 Hz, 2H, H-6 and H-8′), 8.03 (t, *J* = 7.4 Hz, 1H, H-5′), 7.90 (t, *J* = 8.2 Hz, 1H, H-7′), 7.75 (t, *J* = 7.9 Hz, 1H, H-6′). ^13^C NMR (176 MHz, DMSO-d_6_) *δ* 157.6 (C-1), 148.6 (C-2′), 147.1 (C-3), 146.0 (C-8a′), 139.3 (C-5), 138.6 (C-7), 137.0 (C-4′), 131.3 (C-5′), 129.8 (C-6′), 128.7 (C-7′), 128.3 (C-4a′), 128.1 (C-6), 127.5 (C-3′), 123.1 (C-2), 117.3 (C-8). IR (*ν*, cm^−1^): 3068 (w, ν C–H), 1608 (m, ν C=N/C=C), 1576 (m, ν C=C), 1502 (m), 1460 (m), 1384 (m), 1328 (m, ν C–N), 1246 (m), 890 (vs, ReO_4_), 770 (s, *γ* C–H), 748 (s, *γ* C–H). MS (ESI^+^), found: 311.0088 [M − ReO_4_]^+^; calcd for C_16_H_11_N_2_Se: 311.0082.


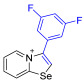


**3u**. A solution of 1-ethynyl-3,5-difluorobenzene (17.4 mg, 126 μmol) in CH_2_Cl_2_ (2 mL) was added to a suspension of 2-pyridylselenyl chloride (24.3 mg, 126 µmol) in CH_2_Cl_2_ (2 mL), and the reaction mixture was stirred at 20 °C for 48 h. Further, Et_2_O was added to the reaction mixture and a colorless precipitate formed, which was washed with Et_2_O (3 × 1 mL) and dried under vacuum. Yield: 32 mg (77%). ^1^H NMR (700 MHz, D_2_O) *δ* 9.75 (d, *J* = 6.7 Hz, 1H, H-5), 9.01–8.98 (m, 2H), 8.65 (d, *J* = 8.6 Hz, 1H, H-8), 8.52 (t, *J* = 8.0 Hz, 1H, H-7), 8.48 (t, *J* = 8.0 Hz, 1H, H-6), 8.03–8.01 (m, 1H), 7.93–7.90 (m, 1H, H-4′). ^13^C NMR (176 MHz, D_2_O) *δ* 163.2 (dd, ^1^*J*_CF_ = 248.5 Hz, ^3^*J*_CF_ = 12.1 Hz, C-3’,5’), 158.9 (NCSe), 139.6 (C-8a), 136.0 (^4^*J*_CF_ = 5.5 Hz, C-3), 130.5 (t, ^3^*J*_CF_ = 10.6 Hz, C-1’), 130.4 (C7), 127.2 (C-5), 126.4 (C-8), 122.3 (C-6), 113.7 (dd, ^2^*J*_CF_ = 21.7, ^4^*J*_CF_ = 5.5 Hz, C-2’,6’), 106.8 (t, ^2^*J*_CF_ = 26.6 Hz, C-4’). IR (*ν*, cm^−1^): 3076 (w, ν C–H), 1612 (m, ν C=N/C=C), 1578 (m, ν C=C), 1510 (m), 1472 (m), 1428 (m), 1356 (m), 1318 (m, ν C–N), 1236 (s, ν C–F), 1174 (s, ν C–F), 1112 (m), 1048 (m), 832 (s, γ C–H), 764 (s, γ C–H). MS (ESI^+^), found: 295.9791 [M − Cl]^+^; calcd for C_13_H_8_F_2_NSe: 295.9785.


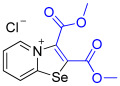


**3v**. A solution of dimethyl acetylenedicarboxylate (14.8 mg, 104 μmol) in CH_2_Cl_2_ (2 mL) was added to a suspension of 2-pyridylselenyl chloride (20 mg, 104 µmol) in CH_2_Cl_2_ (2 mL), and the reaction mixture was stirred at 20 °C for 48 h. Subsequently, formed pale yellow precipitate was separated, and the solid was washed with Et_2_O (3 × 1 mL) and dried under vacuum. Yield: 22 mg (64%). ^1^H NMR (700 MHz, D_2_O) *δ* 9.60 (d, *J* = 6.9 Hz, 1H, H-5), 8.77 (d, *J* = 8.7 Hz, 1H, H-8), 8.36 (t, *J* = 8.0 Hz, 1H, H-7), 8.04 (t, *J* = 7.1 Hz, 1H, H-6), 4.15 (s, 3H, Me), 4.05 (s, 3H, Me). ^13^C NMR (176 MHz, D_2_O) *δ* 161.6 (C=O), 159.2 (C=O), 158.6 (NCSe), 138.7 (C-7), 138.1 (C-5), 133.7 (C-3), 127.4 (C-6), 123.7 (C-8), 55.0 (Me), 54.8 (Me). IR (*ν*, cm^−1^): 3008 (w, arom. C-H), 2952 (w, aliph. C-H), 1751 (m, *ν* C=O ester), 1727 (s, *ν* C=O ester), 1597 (m, C=N/C=C), 1535 (w), 1474 (w), 1328 (m), 1295 (m), 1248 (s, *ν* C-O-C), 1222 (vs, *ν* C-O-C), 1149 (m), 1089 (m), 1053 (m), 1019 (m), 773 (s, *γ* C-H pyridinium), 688 (m), 581 (m). MS (ESI^+^), found: 299.9775 [M − Cl]^+^; calcd for C_11_H_10_NO_4_Se: 299.9770.


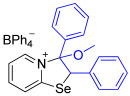


**3w** was prepared according to Method A. 2-Pyridylselenyl chloride (25.1 mg, 130 µmol) and diphenylacetylene (23.2 mg, 130 μmol) were used. Pale yellow precipitate. Yield: 45 mg (50%). ^1^H NMR (700 MHz, C_3_D_6_O) *δ* 8.78 (d, *J* = 6.2 Hz, 1H, H-5), 8.51 (d, *J* = 7.8 Hz, 1H, H-8), 8.46 (t, *J* = 7.9 Hz, 1H, H-7), 7.88 (t, *J* = 6.2 Hz, 1H, H-6), 7.59 (t, *J* = 7.4 Hz, 1H, Ph), 7.53 (t, *J* = 7.8 Hz, 2H, Ph), 7.43 (d, *J* = 8.1 Hz, 2H, Ph), 7.38 (t, *J* = 7.4 Hz, 1H, Ph), 7.36–7.33 (m, 8H, *o*-H_BPh4_), 7.29 (t, *J* = 7.8 Hz, 2H, Ph), 7.21 (d, *J* = 7.9 Hz, 2H, Ph), 6.91 (t, *J* = 7.4 Hz, 8H, *m*-H_BPh4_), 6.77 (t, *J* = 7.2 Hz, 4H, *p*-H_BPh4_), 5.94 (s, 1H, H-2), 3.31 (s, 3H, OMe). 165.1 (q, *J* = 49.2 Hz, *ipso*-C of BPh_4_), 161.2 (NCSe), 146.6 (C-3), 144.8 (Ph), 137.0 (*o*-C of BPh_4_), 132.7 (Ph), 131.8, 131.7 (Ph), 130.4 (Ph), 130.2, 130.0, 126.0 (*m*-C of BPh_4_), 124.2, 122.3 (*p*-C of BPh_4_), 112.0, 59.3 (OMe). IR (*ν*, cm^−1^): 3098 (w, arom C-H), 3056 (w), 3003 (w), 1621 (w, C=N/C=C), 1591 (m), 1501 (m), 1478 (m), 1449 (m), 1326 (m), 1259 (m), 1143 (m), 1122 (m), 1033 (m), 933 (m), 823 (m), 783 (m, *γ* C-H), 734 (vs, BPh4), 703 (vs, BPh4). MS (ESI^+^), found: 368.0558 [M − BPh_4_]^+^; calcd for C_20_H_18_NOSe: 368.0548.

## 4. Conclusions

In summary, here we introduced a robust and versatile synthetic route to pyridinium-fused 1,3-selenazoles through the electrophilic cyclization of 2-pyridylselenyl chloride with a diverse range of alkynes.

The work uncovered a notable regiochemical divergence, where most alkynes yield exclusively the 3-substituted isomer, while 2-pyridyl- and 2-quinolylacetylenes produce regioisomeric mixtures. DFT calculations elucidated that this behavior stems from a competition between kinetic and thermodynamic control. The formation of the 2-isomer is thermodynamically stabilized by an ancillary chalcogen bond between the selenium atom and the pyridine nitrogen of the alkyne substituent, a distinctive feature absent in non-nitrogenous analogs.

Comprehensive structural analysis, combining single-crystal X-ray diffraction with advanced computational tools (MEP, QTAIM, and NBO), provided deep insight into the supramolecular architectures of the synthesized salts in the solid state. The results highlight the significant and directional role of selenium-centered σ-hole interactions in crystal packing. The strength and prevalence of these interactions are modulated by the counterion’s nature, with more nucleophilic anions like chloride engaging in stronger, charge-transfer-assisted chalcogen bonds that dominate the solid-state assembly.

Finally, preliminary antifungal screening identified several compounds, notably **3b**, **3l**, and **3r**, with promising and specific activity against key phytopathogenic fungi, sometimes surpassing the commercial reference fungicide. This biological evaluation demonstrates the potential of this novel class of selenium-containing heterocycles in agrochemical applications.

## Figures and Tables

**Table 1:** Growth inhibition of phytopathogenic fungi in the presence of compounds (**a**–**v**).

		Mycelium Growth Inhibition, %				
	*R.s. ^a^*	*V.i. ^b^*	*F.m. ^c^*	*B.s. ^d^*	*S.s. ^e^*	*F.o. ^f^*
**a**	23	8	20	34	7	13
**b**	27	14	36	100	24	25
**c**	17	23	9	44	0	12
**g**	−9	0	14	25	0	13
**h**	25	8	14	55	14	10
**i**	9	14	7	25	0	0
**j**	7	11	20	33	2	16
**k**	9	14	29	43	0	0
**l**	74	100	84	100	100	63
**m**	0	−4	10	41	−10	9
**n**	23	33	23	65	35	18
**o**	17	15	16	40	9	13
**r**	58	100	84	100	90	24
**s**	0	7	20	36	10	4
**u**	23	0	30	87	8	21
**v**	28	18	26	25	17	10
TDF ^g^	68	61	83	73	57	68
NaBF_4_	40	20	13	47	15	11
2-Ethynylquinoline	100	53	82	100	28	56
Quinoline	30	25	25	33	15	5

*^a^ Rhizoctonia solani; ^b^ Venturia inaequalis; ^c^ Fusarium moniliforme; ^d^ Bipolaris sorokiniana. ^e^ Sclerotinia sclerotiorum; ^f^ Fusarium oxysporum; ^g^ Triadimefon.*

**Table 2:** Growth inhibition of phytopathogenic fungi in the presence of compounds **3l** and **3r**.

		Mycelium Growth Inhibition, %				
	*R.s. ^a^*	*V.i. ^b^*	*F.m. ^c^*	*B.s. ^d^*	*S.s. ^e^*	*F.o. ^f^*
**3l**	74	100	84	100	100	63
**3r**	58	100	84	100	90	24
**Ethynylquinoline**	100	53	79	100	28	56
**Quinoline**	30	25	25	33	15	5
**TDF *^g^***	68	61	83	73	57	68

*^a^ Rhizoctonia solani; ^b^ Venturia inaequalis; ^c^ Fusarium moniliforme; ^d^ Bipolaris sorokiniana. ^e^ Sclerotinia sclerotiorum; ^f^ Fusarium oxysporum; ^g^ Triadimefon.*

## Data Availability

The original contributions presented in this study are included in the article/Supplementary Material. Further inquiries can be directed to the corresponding author.

## Supplementary Materials

The following supporting information can be downloaded at: https://www.mdpi.com/article/10.3390/ijms27062908/s1, References [79,80,81,82,83] are cited in the supplementary materials.
