# Supplementary material for: Pyridinium-Fused 1,3-Selenazoles via Cyclizations of 2-Pyridylselenyl Chloride with Alkynes: Synthesis, Structures, and Antifungal Properties
Source: Int J Mol Sci. 2026 Mar 23;27(6):2908. doi: 10.3390/ijms27062908 (PMC13026896; doi:10.3390/ijms27062908)

Supporting information

X-ray crystal structure determination

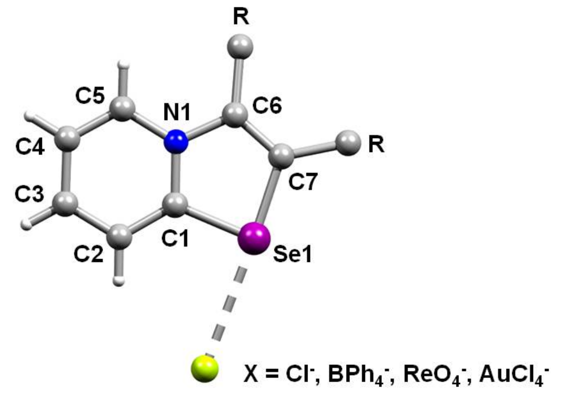

Figure S1. Atom numbering scheme in compounds.

The single-crystal X-ray diffraction data for compounds **3d**, **3k**, **3p**, **3u-3w** were obtained at N.D. Zelinsky Institute of Organic Chemistry of the Russian Academy of Sciences on a four-circle XtaLAB Rigaku Synergy-S diffractometer equipped with a HyPix-6000HE area-detector (T = 100 K, λ(Cu$K\alpha$)-radiation, graphite monochromator, shutterless ω-scan mode). The data were integrated and corrected for absorption by the *CrysAlisPro* program [1]. The single-crystal X-ray diffraction data for **3a-3c, 3f-3i, 3l-3o** and **3q-3t** were obtained at the Collective Use Center of the Institute of General and Inorganic Chemistry of the Russian Academy of Sciences on a three-circle Bruker D8 Venture (T = 100 K, graphite monochromator, ω and φ scanning mode). The data were indexed and integrated using the *SAINT* program [2], and then scaled and corrected for absorption using the *SADABS* program [3]. For details, see Table S1.

The structures were determined by direct methods and refined by full-matrix least squares technique on F^2 with anisotropic displacement parameters for non-hydrogen atoms. Hydrogen atoms are calculated from geometric considerations. All calculations were carried out using the SHELXL program [4] and OLEX2 program package [5].

Crystallographic data for all investigated compounds have been deposited with the Cambridge Crystallographic Data Center, CCDC 2524056-2524074. Copies of this information may be obtained free of charge from the Director, CCDC, 12 Union Road, Cambridge CB2 1EZ, UK (Fax: +44 1223 336033; e-mail: deposit@ccdc.cam.ac.uk or www.ccdc.cam.ac.uk).

References

[1] Rigaku, CrysAlisPro Software System, v. 1.171.41.106a, Rigaku Oxford Diffraction, 2021.

[2] Bruker, *SAINT*, *v. 8.40A*, Bruker AXS Inc., Madison, WI, **2019**.

[3] L. Krause, R. Herbst-Irmer, G. M. Sheldrick, D. Stalke, *J. Appl. Cryst.* **2015**, *48*, 3-10.

[4] G. M. Sheldrick, *Acta Cryst.* **2015**, *C71*, 3-8.

[5] O.V. Dolomanov, L.J. Bourhis, R.J. Gildea, J.A.K. Howard, H. Puschmann. *J. Appl. Cryst*. **2009** *42*, 339 – 3

Table S1. Selected bond lengths (Å) for compounds **3a–3w**.

	**Se1−C1**	**Se1−C7**	**C1−N1**	**C6−N1**	**C6−C7**
**3a**	1.861(2)	1.860(3)	1.375(3)	1.430(3)	1.350(3)
**3b**	1.872(9)	1.895(10)	1.374(12)	1.435(11)	1.359(13)
**3c**	1.864(9)	1.867(10)	1.386(11)	1.439(11)	1.336(13)
**3d**	1.852(6)	1.866(7)	1.368(8)	1.418(8)	1.345(10)
**3f**	1.865(12)	1.878(11)	1.393(14)	1.444(14)	1.328(15)
**3g**	1.862(3)	1.859(3)	1.364(4)	1.428(4)	1.347(5)
**3h**	1.865(3)	1.865(3)	1.362(4)	1.424(3)	1.346(4)
**3i**	1.856(4)	1.849(4)	1.377(4)	1.419(4)	1.338(5)
**3k**	1.865(4)	1.874(4)	1.367(4)	1.420(4)	1.349(5)
**3l**	1.862(3)	1.857(3)	1.366(3)	1.416(3)	1.341(3)
**3m**	1.856(4)	1.851(5)	1.376(6)	1.402(5)	1.352(6)
**3o**	1.869(2)	1.877(2)	1.369(3)	1.405(3)	1.347(3)
**3p**	1.859(2)	1.876(2)	1.364(3)	1.401(3)	1.342(3)
**3q**	1.865(2)	1.850(2)	1.373(3)	1.426(3)	1.347(3)
**3s**	1.863(3)	1.876(3)	1.369(3)	1.421(4)	1.330(4)
**3t**	1.863(6)	1.877(5)	1.363(7)	1.447(7)	1.357(8)
**3u**	1.876(3)	1.861(3)	1.372(4)	1.418(4)	1.334(4)
**3v**	1.865(2)	1.876(2)	1.369(2)	1.405(2)	1.348(2)
**3w**	1.855(4)	1.948(4)	1.363(5)	1.500(5)	1.552(6)

Table S2. Selected bond angles (˚) for compounds **3a–3w**.

	**∠C1−Se1−C7**	**∠Se1−C1−N1**	**∠Se1−C7−C6**	**∠C1−N1−C6**	**∠N1−C6−C7**
**3a**	85.95(11)	111.79(18)	114.3(2)	115.3(2)	112.6(2)
**3b**	85.4(4)	112.3(6)	113.8(7)	115.6(7)	112.7(8)
**3c**	86.0(4)	111.5(6)	114.6(7)	115.0(7)	112.8(8)
**3d**	85.7(3)	112.1(5)	113.9(5)	115.3(6)	113.0(6)
**3f**	86.6(5)	110.2(8)	114.5(8)	116.1(9)	112.5(10)
**3g**	86.08(15)	111.3(2)	114.1(3)	116.0(3)	112.5(3)
**3h**	85.45(12)	112.25(19)	114.0(2)	115.1(2)	113.1(3)
**3i**	85.63(17)	112.1(3)	114.5(3)	114.4(3)	113.3(3)
**3k**	85.94(15)	111.6(2)	113.3(3)	115.7(3)	113.4(3)
**3l**	85.81(11)	111.39(18)	114.13(19)	115.7(2)	112.9(2)
**3m**	86.14(19)	111.4(3)	113.5(3)	115.3(4)	113.6(4)
**3o**	85.78(10)	111.68(16)	112.67(17)	115.26(19)	114.6(2)
**3p**	85.9(1)	111.48(17)	112.59(18)	115.7(2)	114.3(2)
**3q**	84.96(9)	112.62(15)	115.49(16)	114.57(17)	112.29(19)
**3s**	85.35(12)	111.84(19)	114.1(2)	115.3(2)	113.4(3)
**3t**	85.6(2)	111.9(4)	114.9(4)	116.5(5)	111.0(5)
**3u**	85.20(13)	111.8(2)	114.5(2)	114.8(2)	113.7(3)
**3v**	85.19(7)	112.15(12)	113.63(12)	115.40(14)	113.62(15)
**3w**	85.76(17)	114.9(3)	105.8(3)	114.9(3)	104.8(3)

NMR spectra for **3a-3w**

^{1}H NMR spectrum (700 MHz, CD_2Cl_2) of **3a**

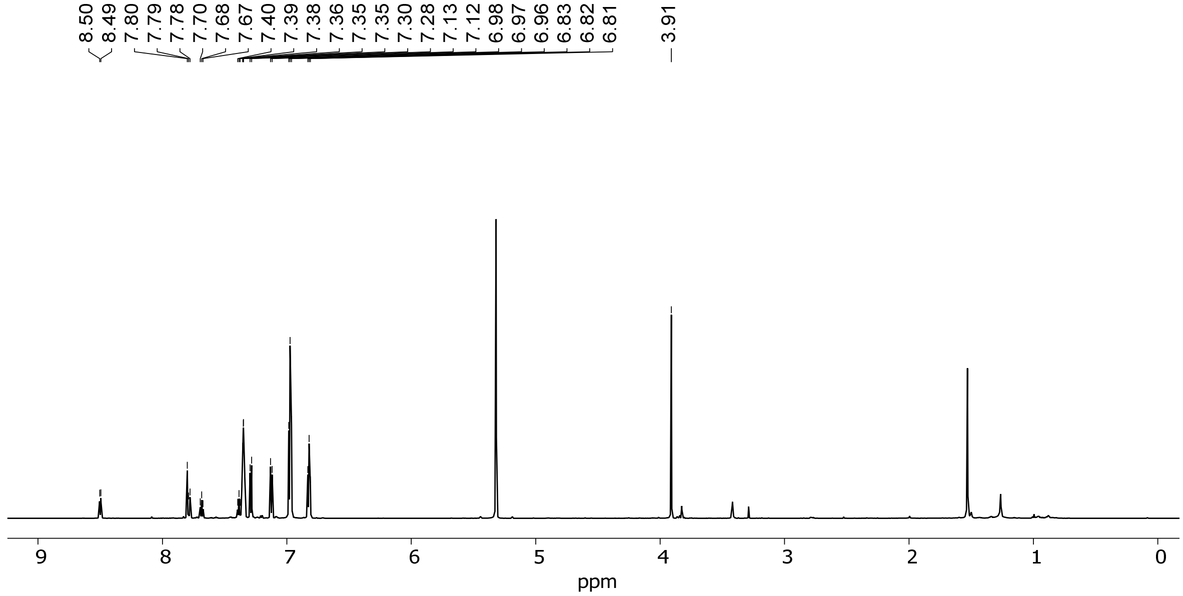

^{13}C NMR spectrum (176 MHz, CD_2Cl_2) of **3a**

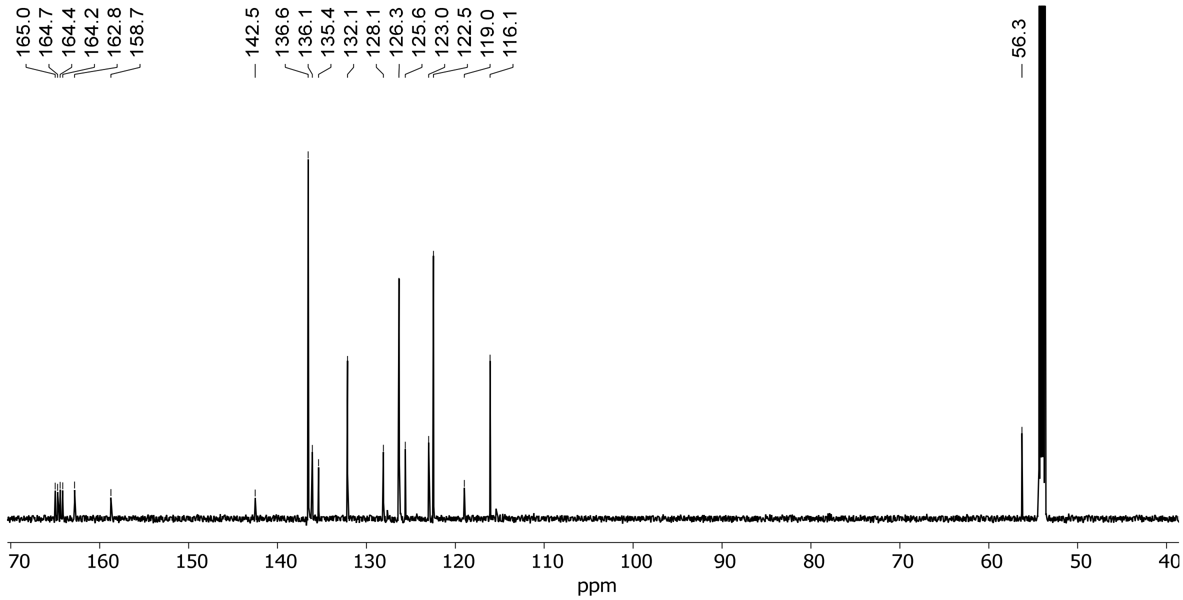

^{1}H NMR spectrum (700 MHz, $CDCl_3$) of **3b**

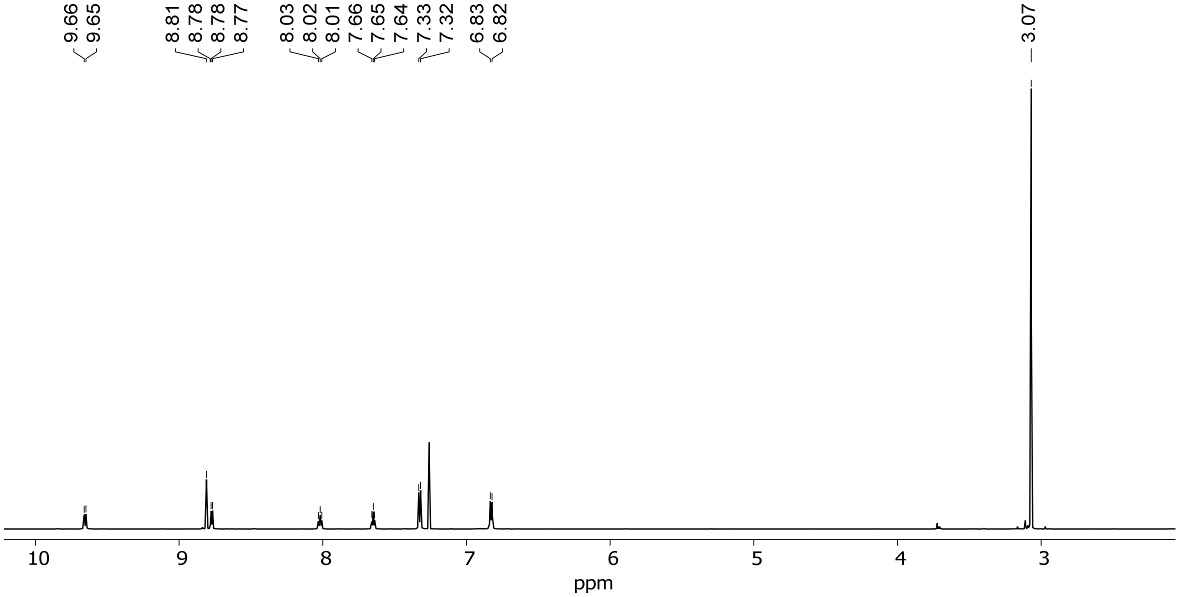

^{13}C NMR spectrum (176 MHz, $CDCl_3$) of **3b**

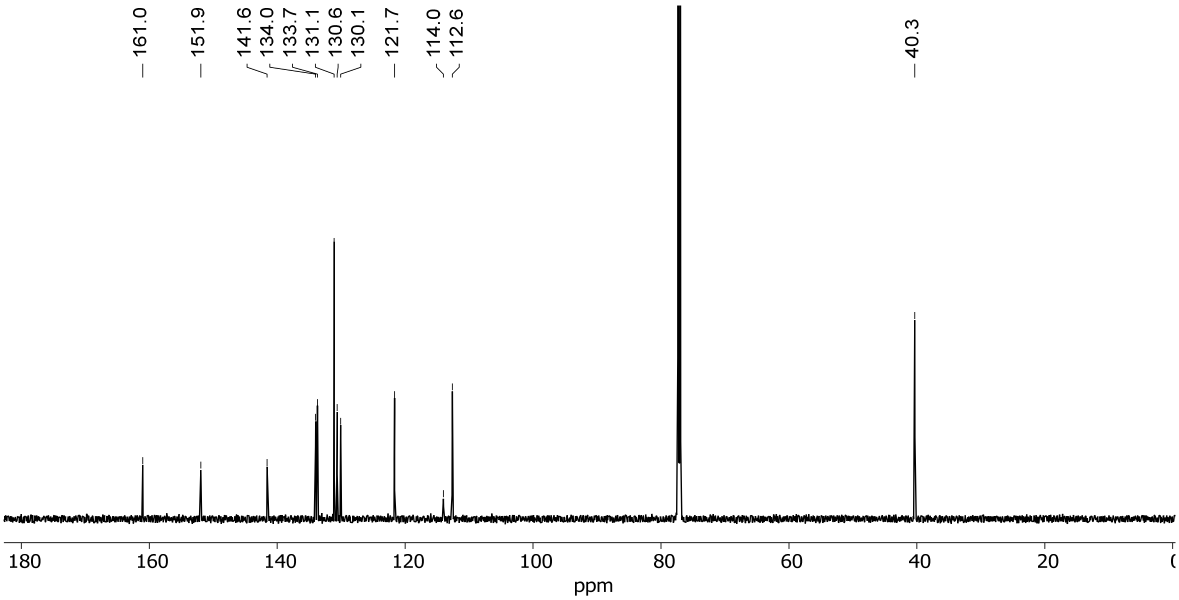

^{1}H NMR spectrum (700 MHz, CD_2Cl_2) of **3c**

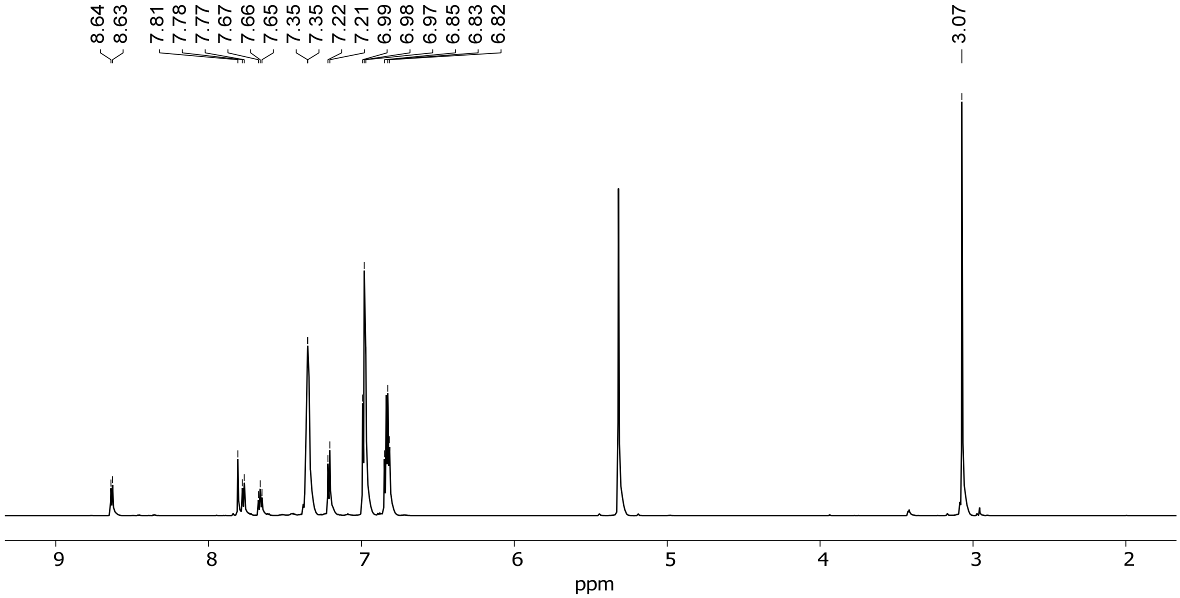

^{13}C NMR spectrum (176 MHz, CD_2Cl_2) of **3c**

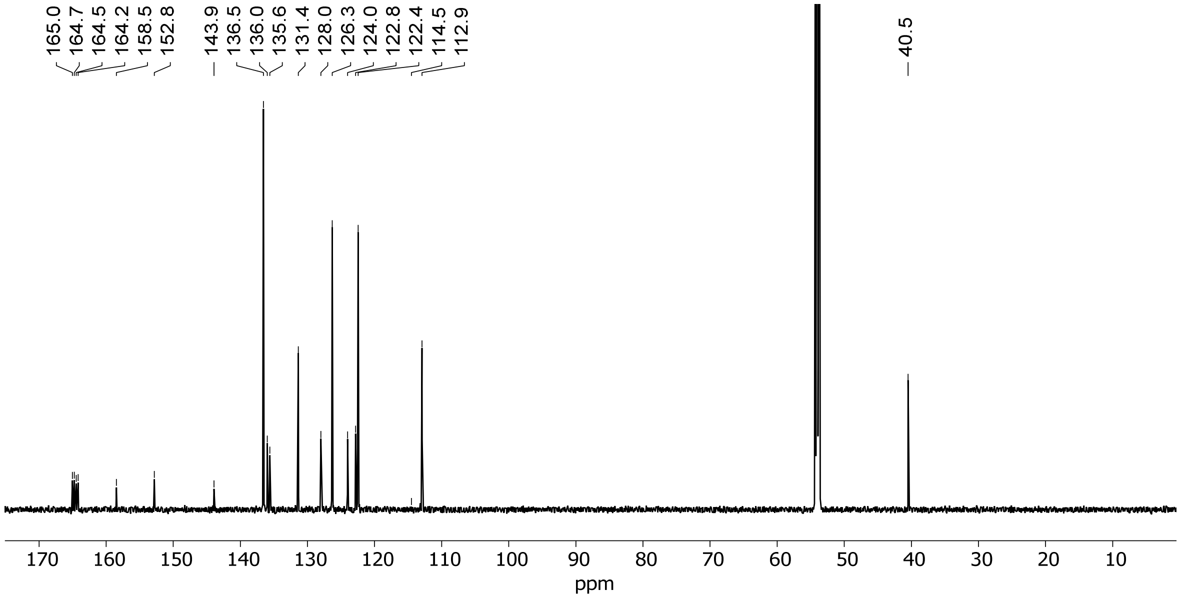

^{1}H NMR spectrum (700 MHz, C_3D_6O) of **3d**

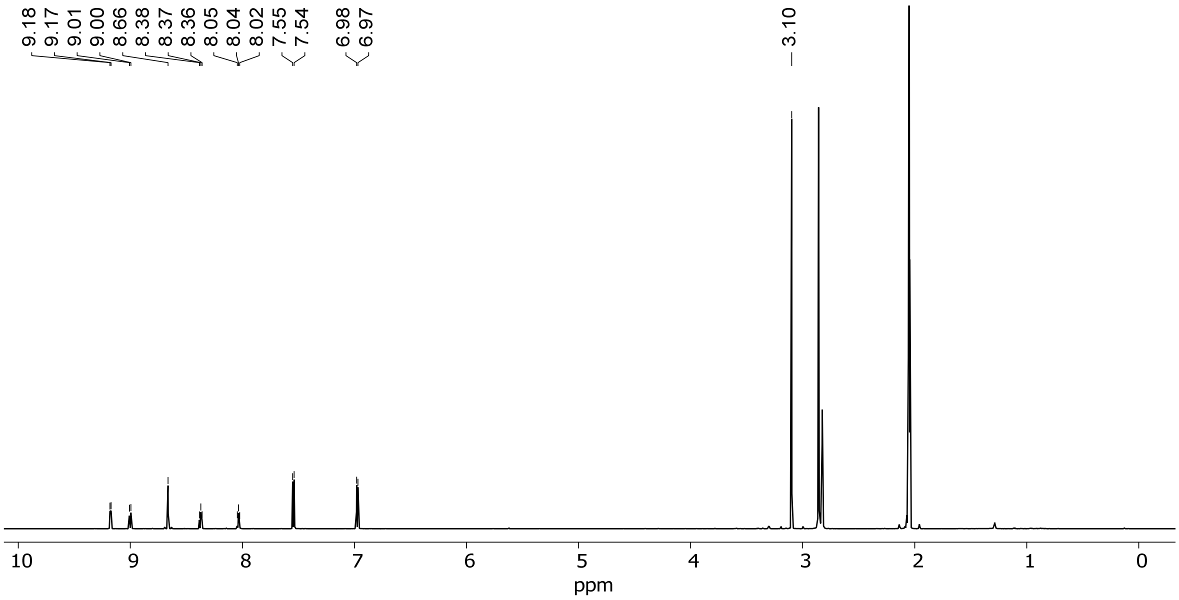

^{13}C NMR spectrum (176 MHz, C_3D_6O) of **3d**

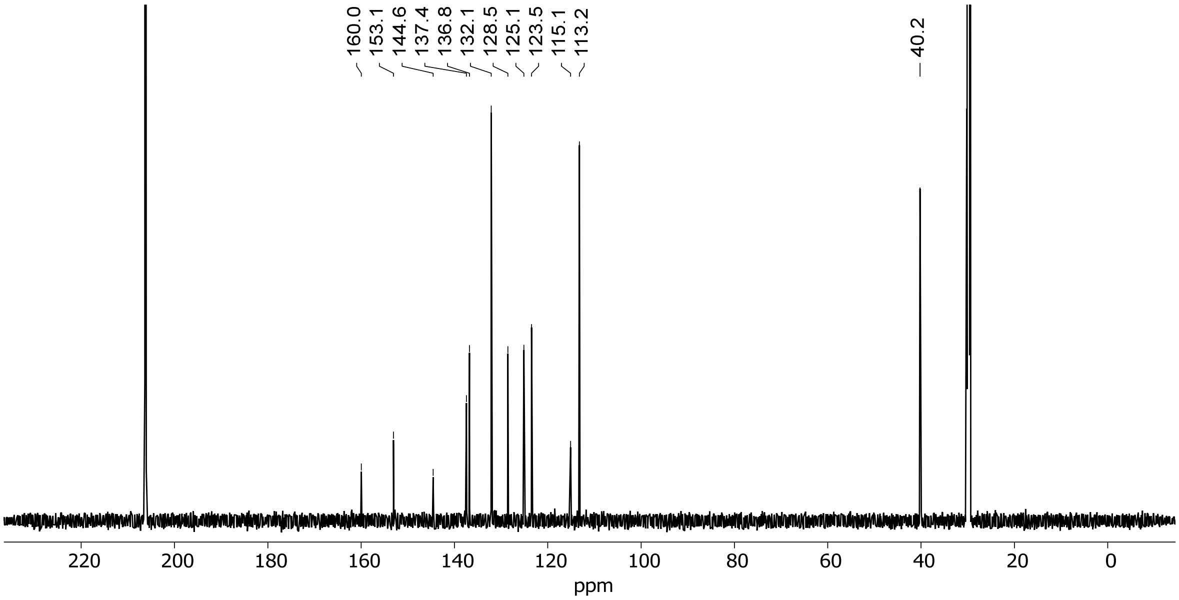

^{1}H NMR spectrum (700 MHz, D_2O) of **3e**

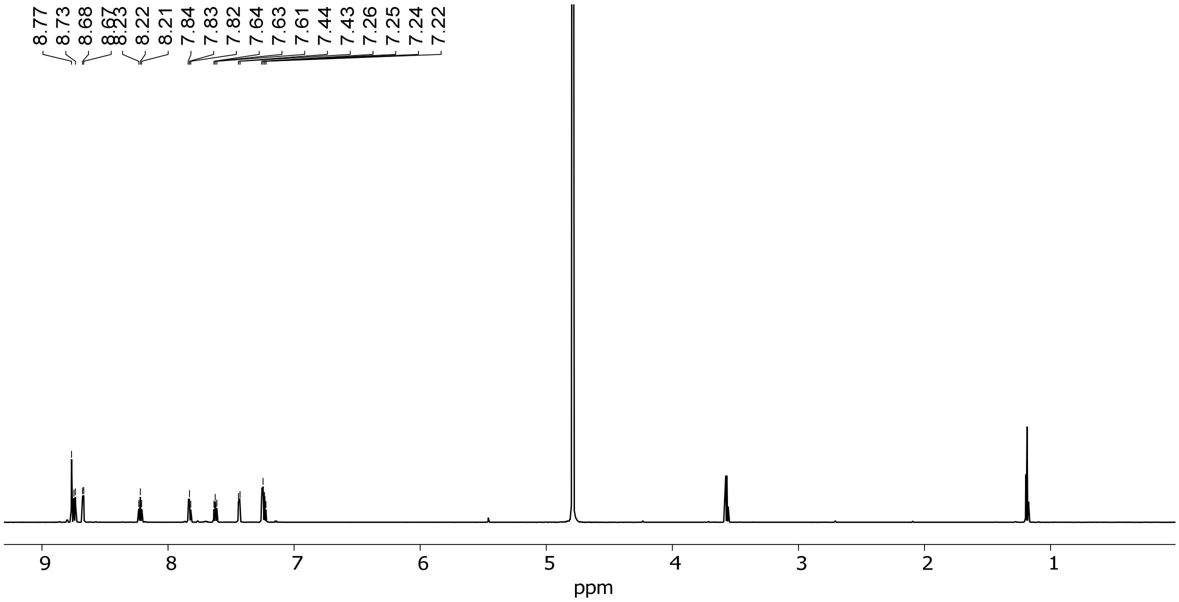

^{13}C NMR spectrum (176 MHz, D_2O) of **3e**

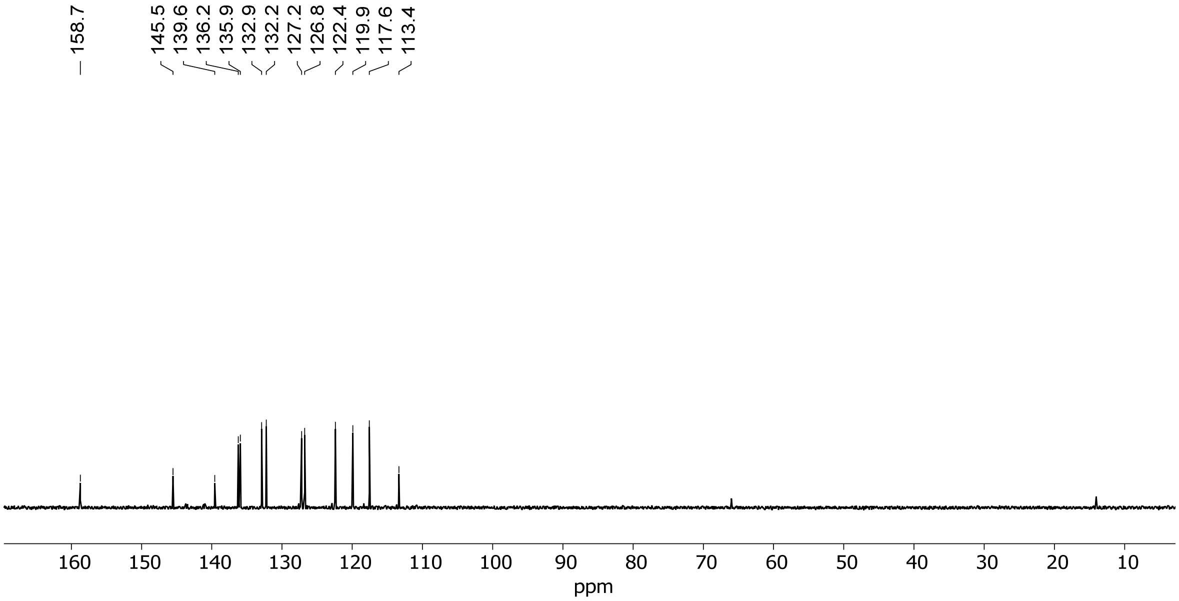

^{1}H NMR spectrum (700 MHz, DMSO-d$_6$) of **3f**

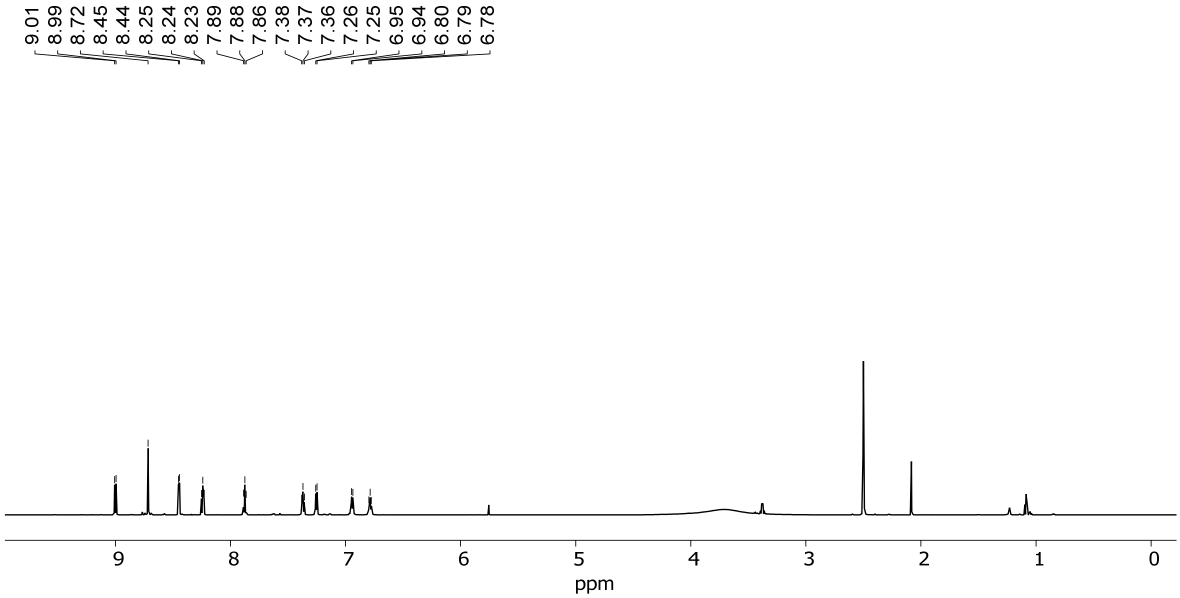

^{13}C NMR spectrum (176 MHz, DMSO-d$_6$) of **3f**

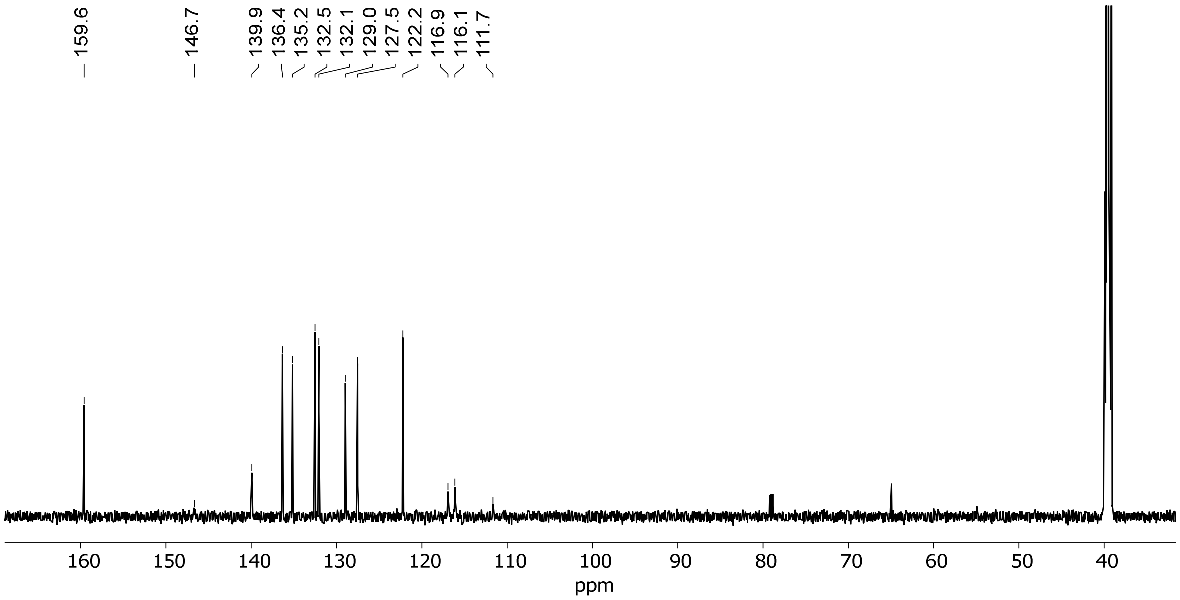

^{1}H NMR spectrum (700 MHz, CD_2Cl_2) of **3g**

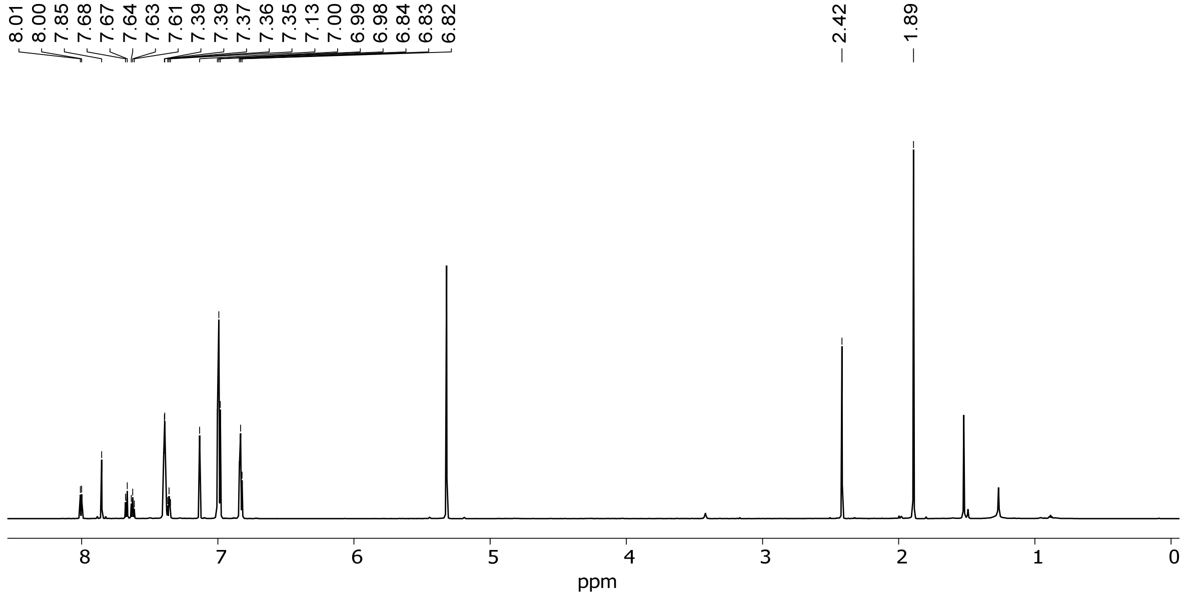

^{13}C NMR spectrum (176 MHz, CD_2Cl_2) of **3g**

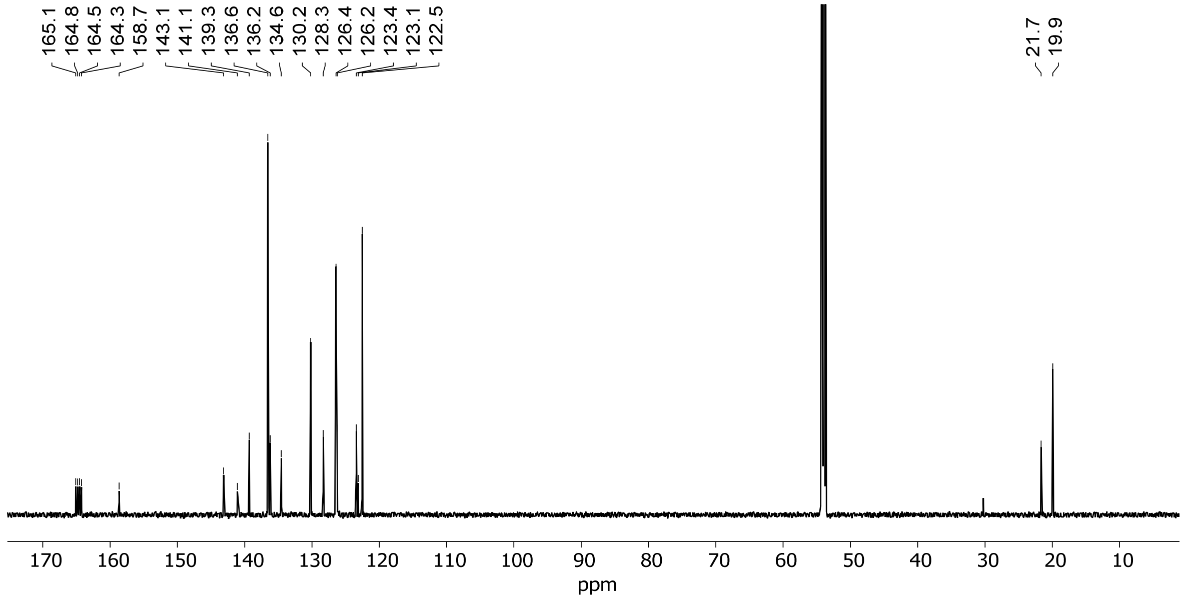

^{1}H NMR spectrum (700 MHz, CD_3CN) of **3h**

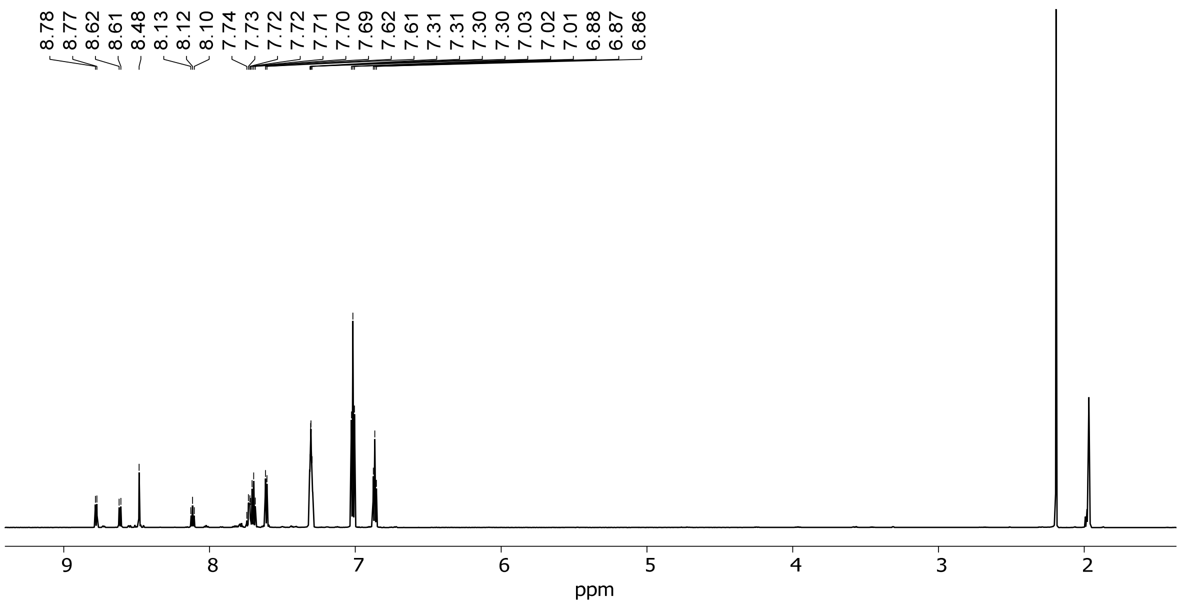

^{13}C NMR spectrum (700 MHz, CD_3CN) of **3h**

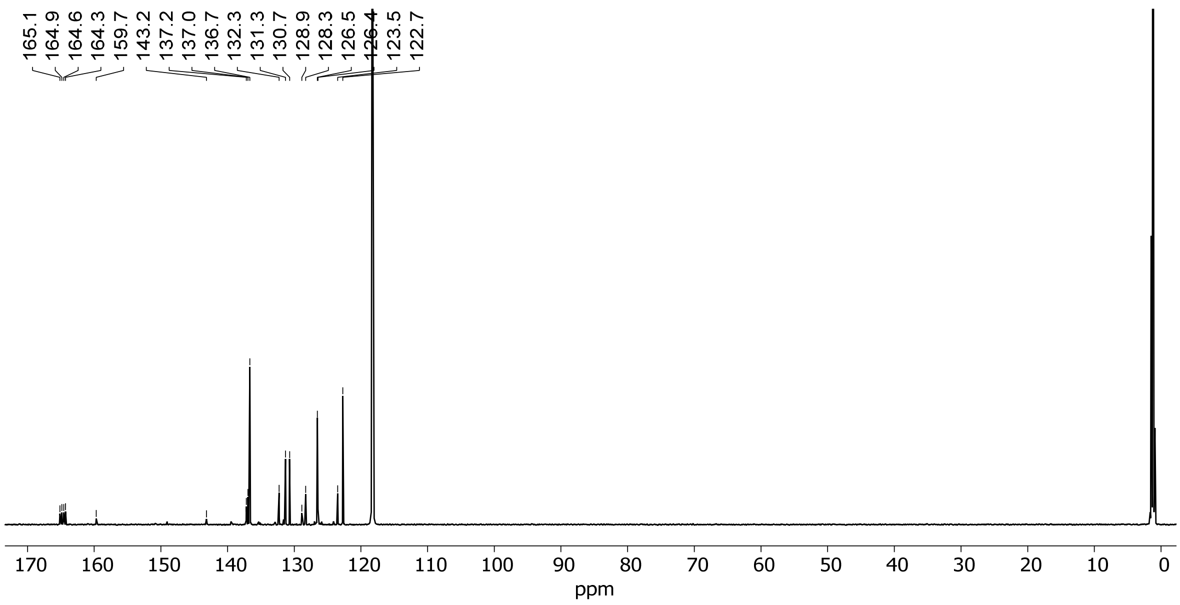

^{1}H NMR spectrum (700 MHz, C_3D_6O) of **3i**

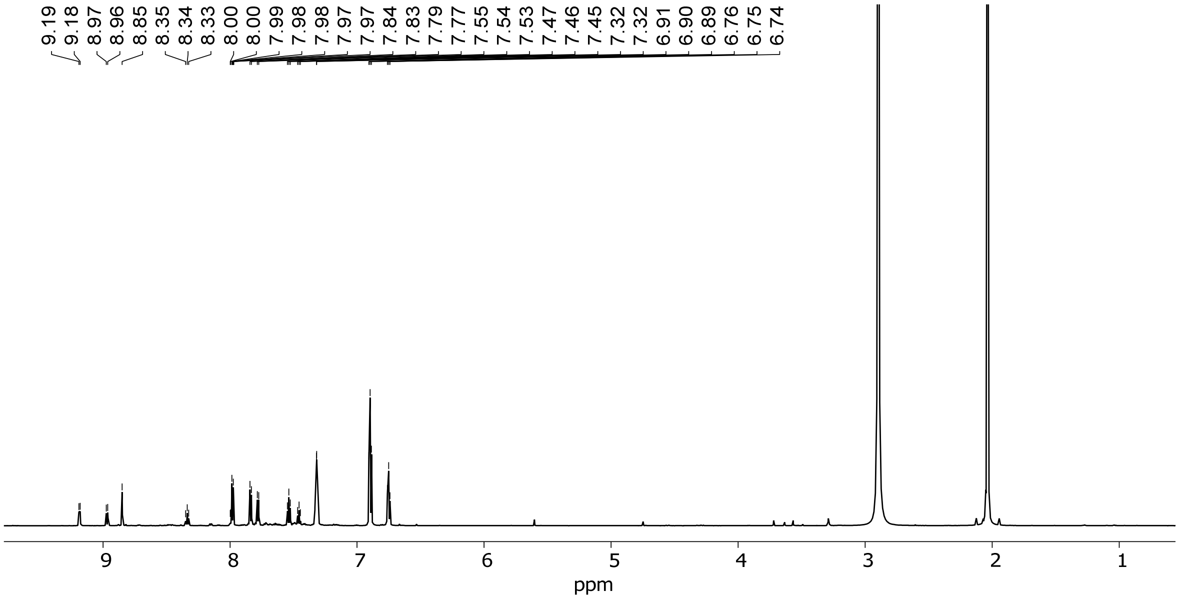

^{13}C NMR spectrum (700 MHz, C_3D_6O) of **3i**

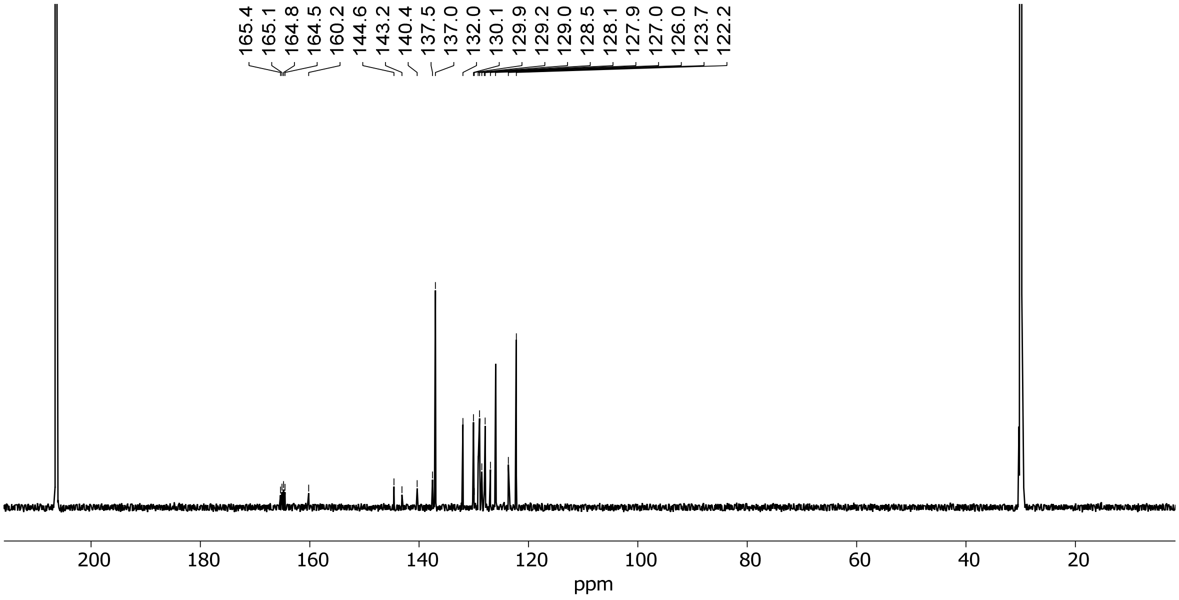

^{1}H NMR spectrum (700 MHz, C_3D_6O) of **3j**

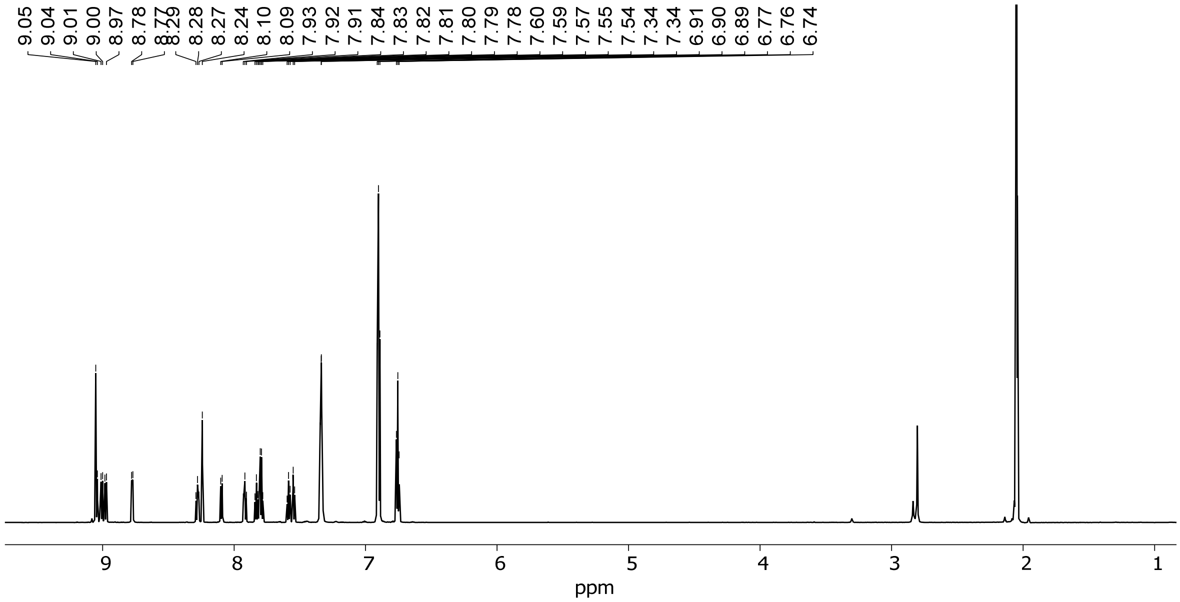

^{13}C NMR spectrum (176 MHz, C_3D_6O) of **3j**

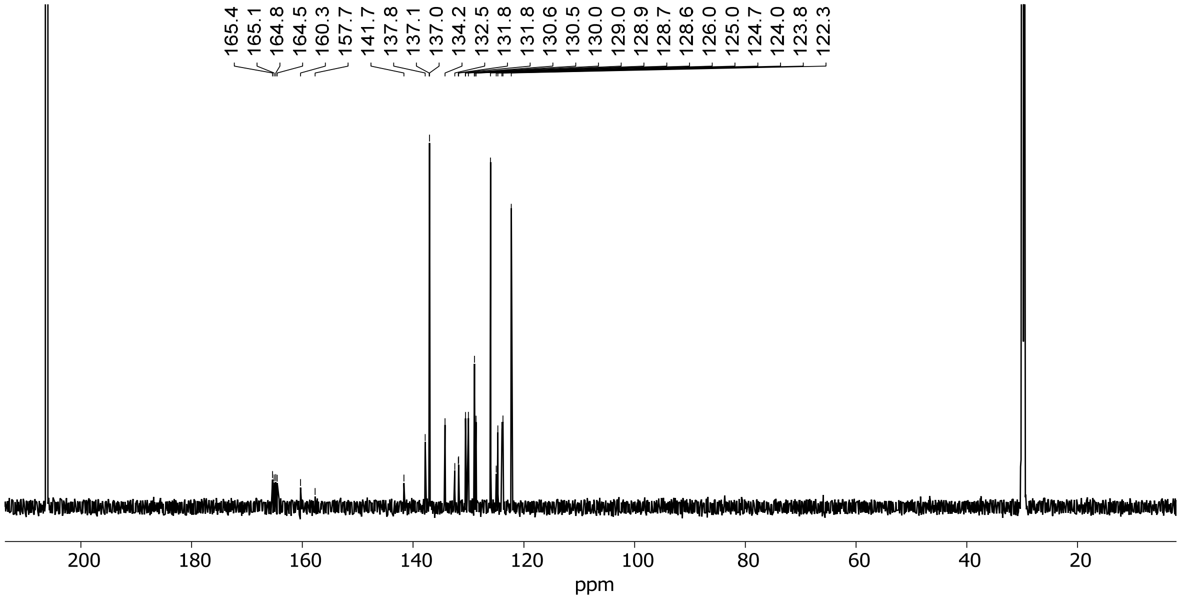

^{1}H NMR spectrum (700 MHz, C_3D_6O) of **3k**

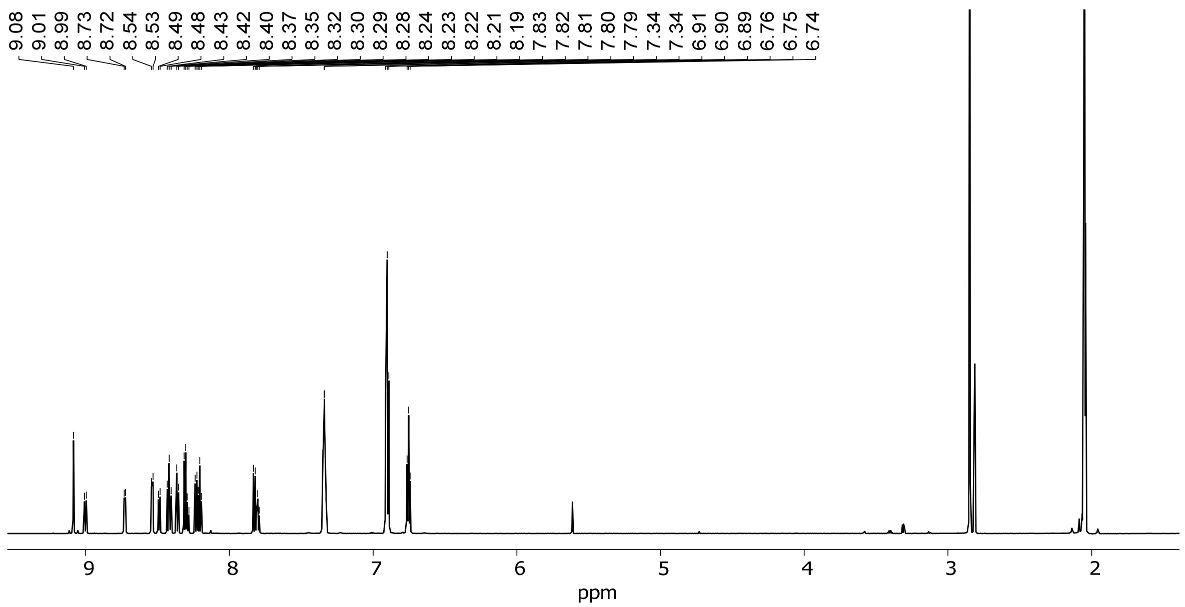

^{13}C NMR spectrum (176 MHz, C_3D_6O) of **3k**

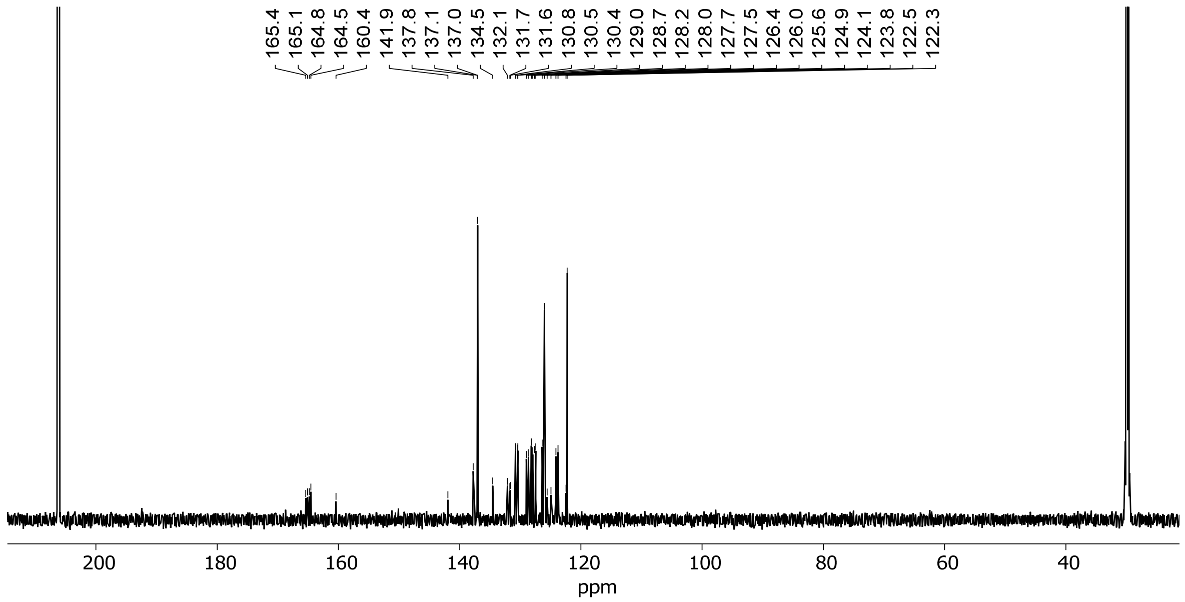

^{1}H NMR spectrum (700 MHz, D_2O) of **3l**

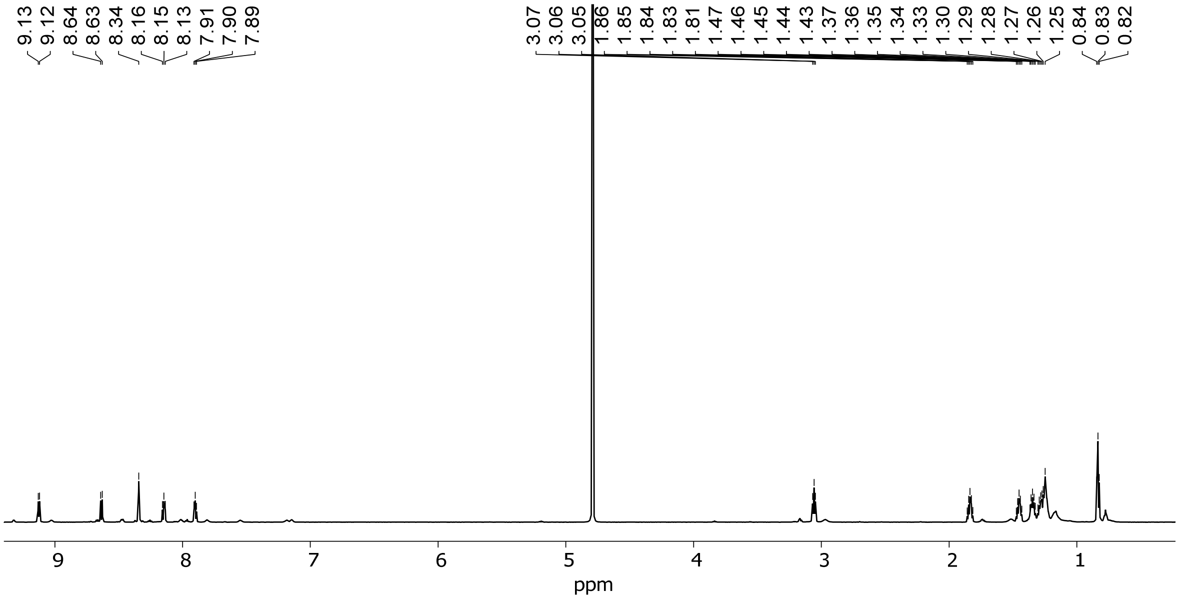

^{13}C NMR spectrum (176 MHz, D_2O) of **3l**

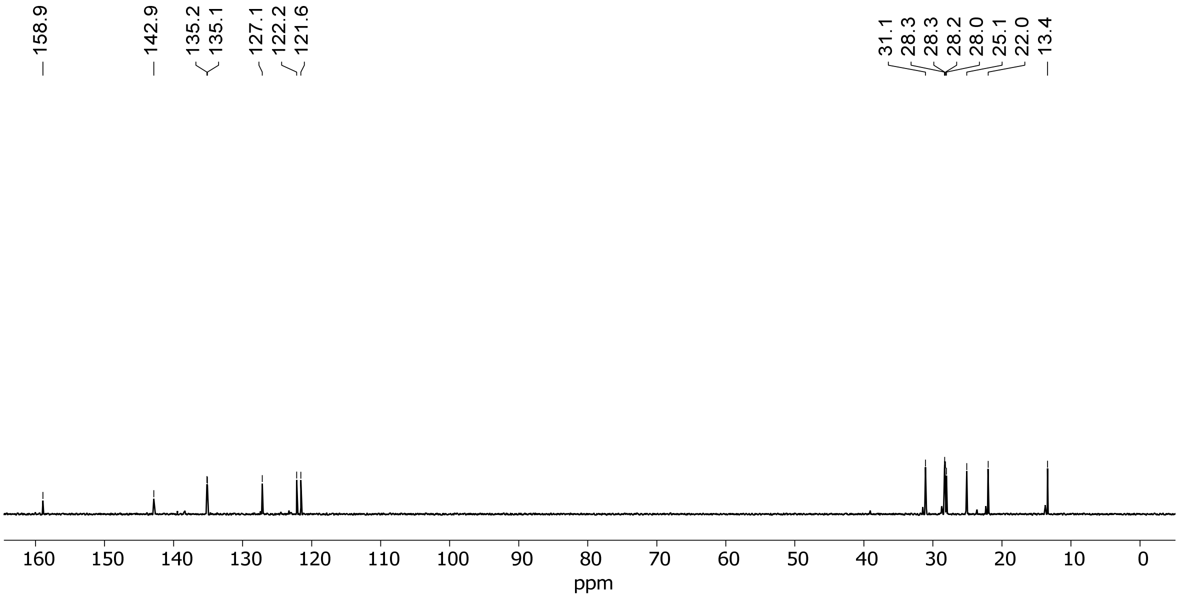

^{1}H NMR spectrum (700 MHz, C_3D_6O) of **3m**

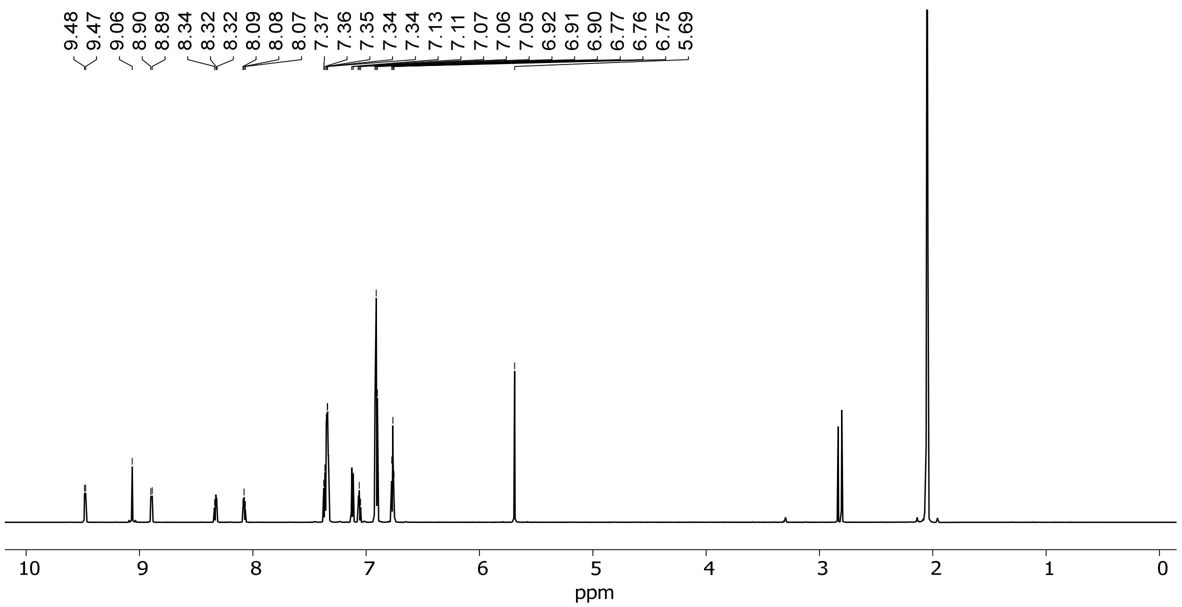

^{13}C NMR spectrum (176 MHz, C_3D_6O) of **3m**

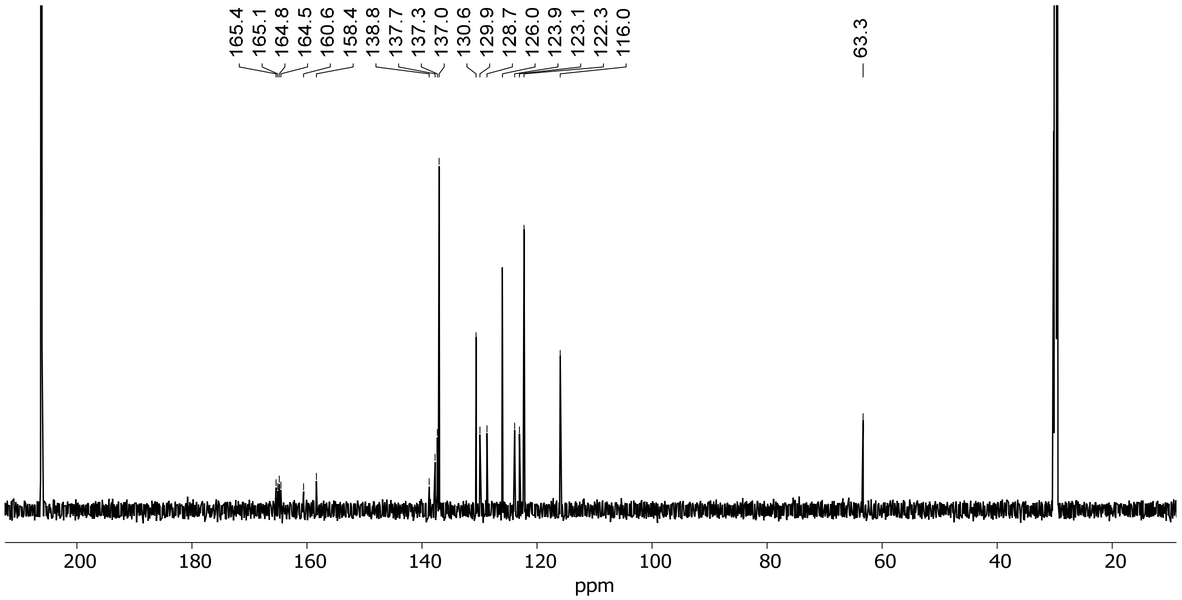

^{1}H NMR spectrum (700 MHz, D_2O) of **3n**

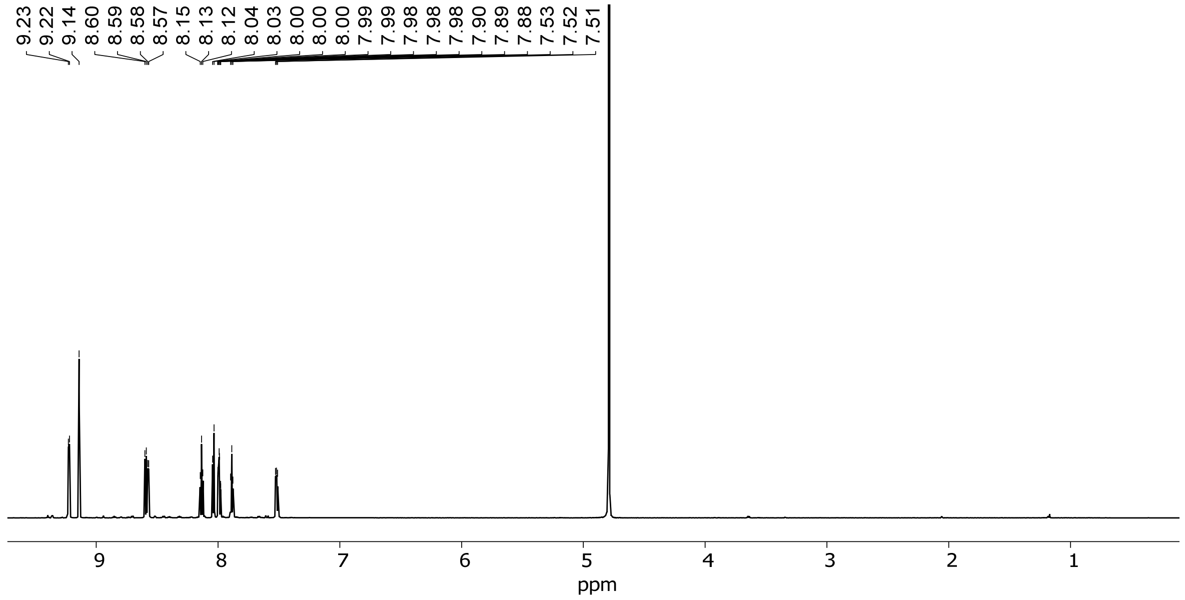

^{13}C NMR spectrum (176 MHz, D_2O) of **3n**

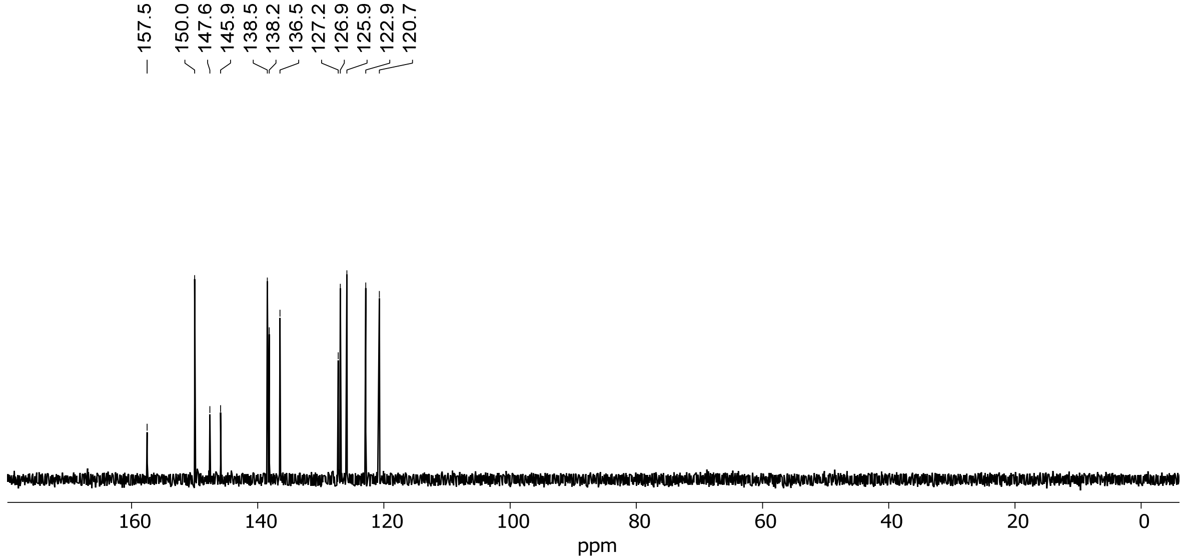

^{1}H NMR spectrum (700 MHz, CD_3CN) of **3o**

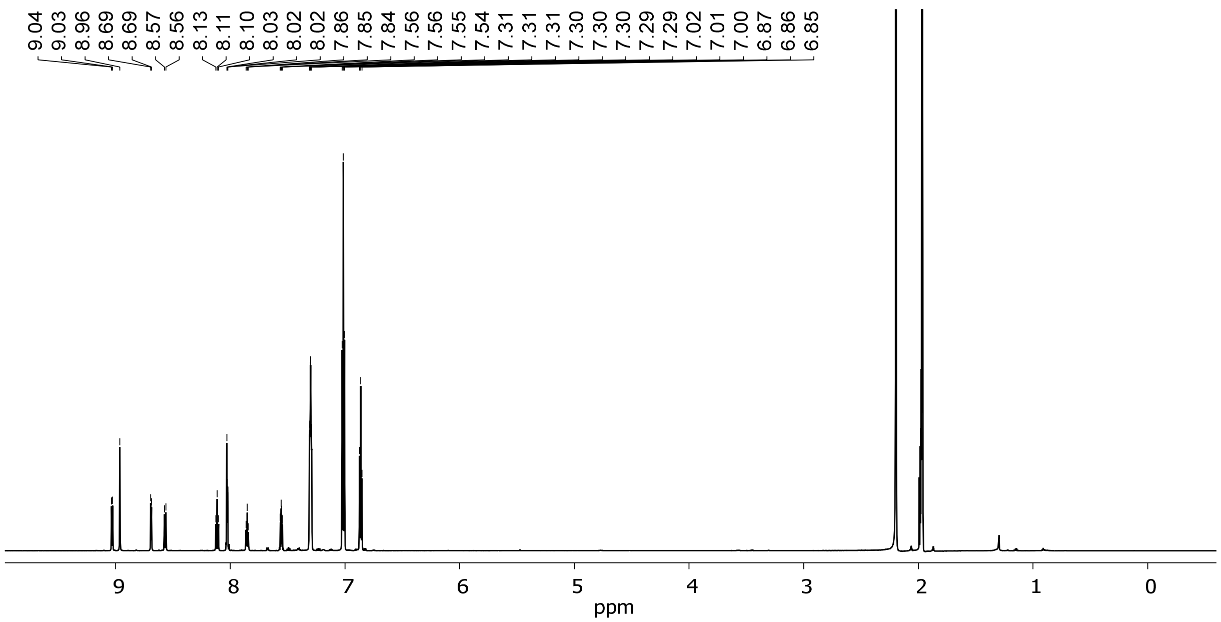

^{13}C NMR spectrum (176 MHz, CD_3CN) of **3o**

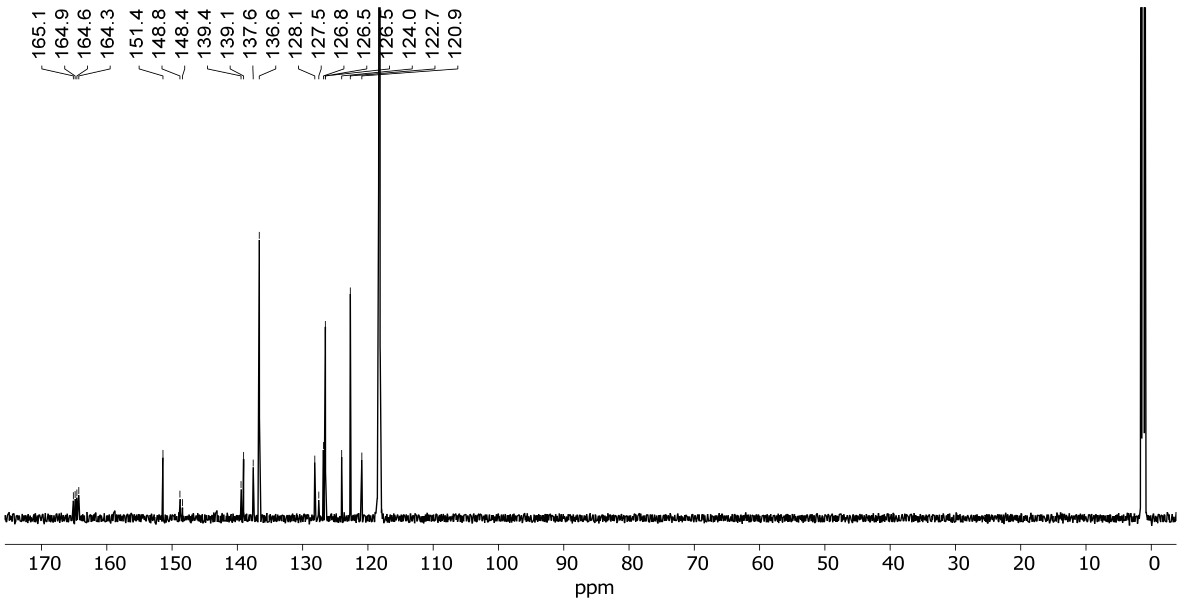

^{1}H NMR spectrum (700 MHz, C_3D_6O) of **3p**

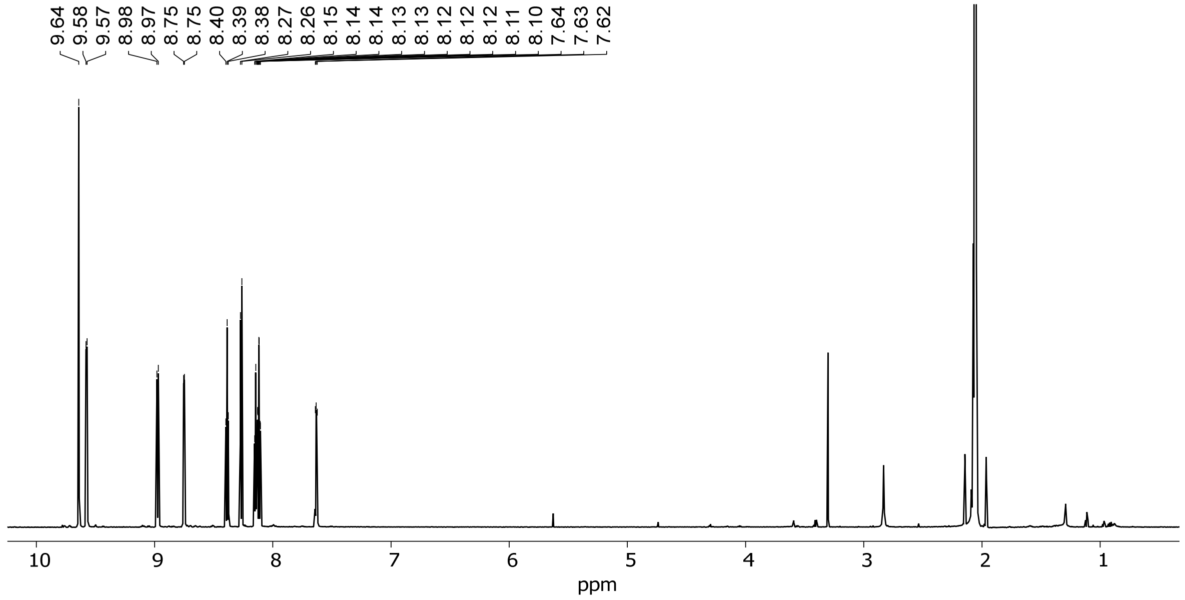

^{13}C NMR spectrum (176 MHz, C_3D_6O) of **3p**

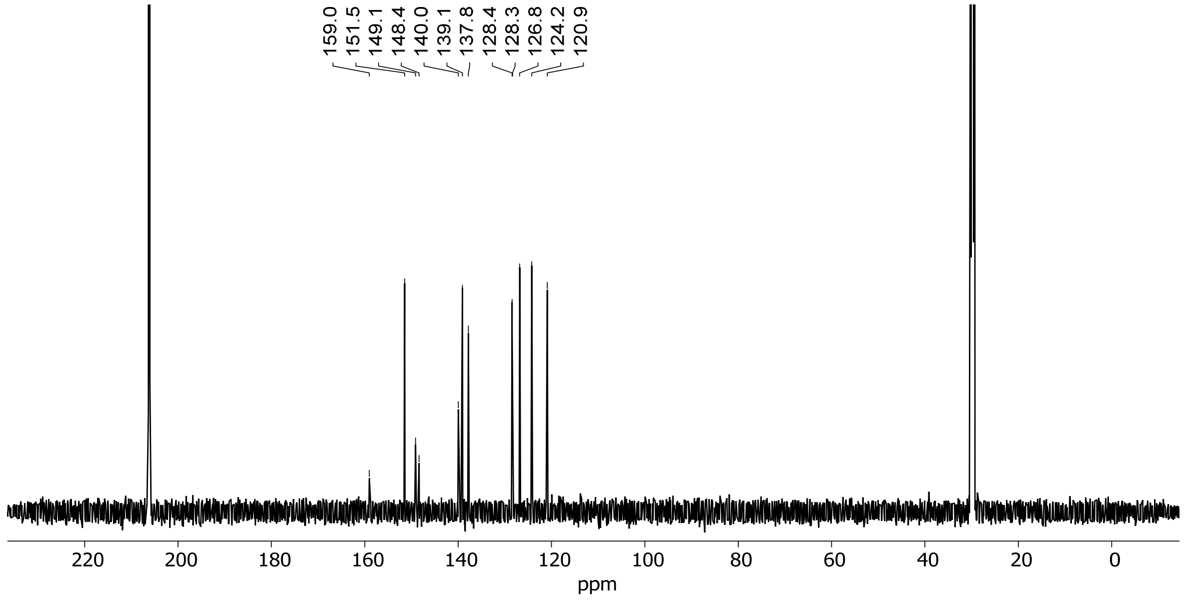

^{1}H NMR spectrum (700 MHz, D_2O) of **3q**

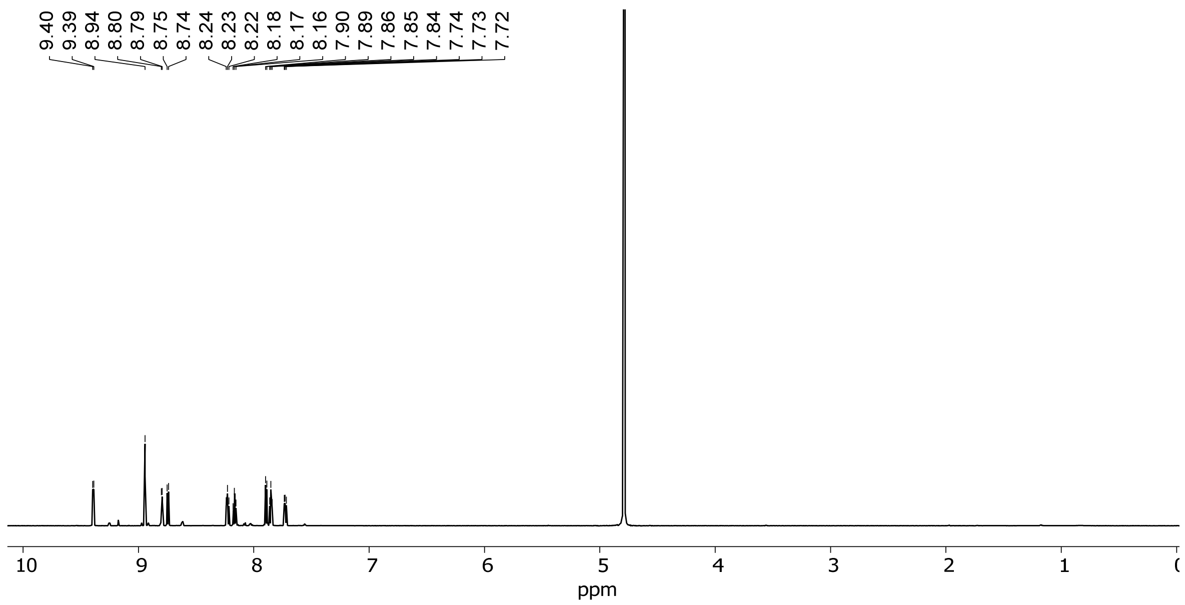

^{13}C NMR spectrum (176 MHz, D_2O) of **3q**

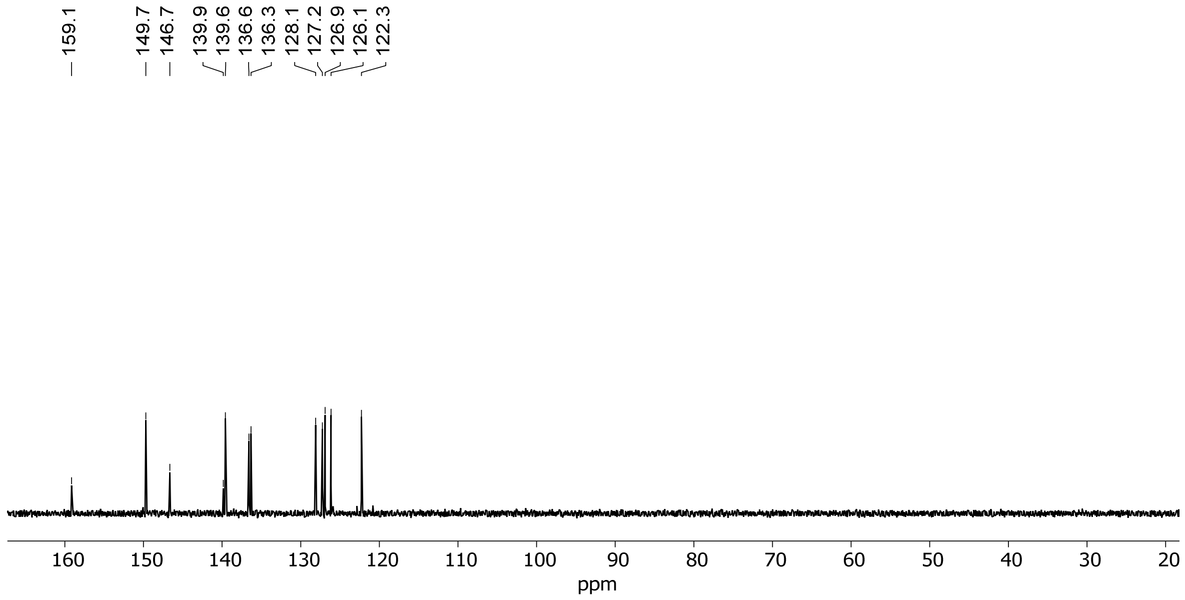

^{1}H NMR spectrum (700 MHz, D_2O) of **3r**

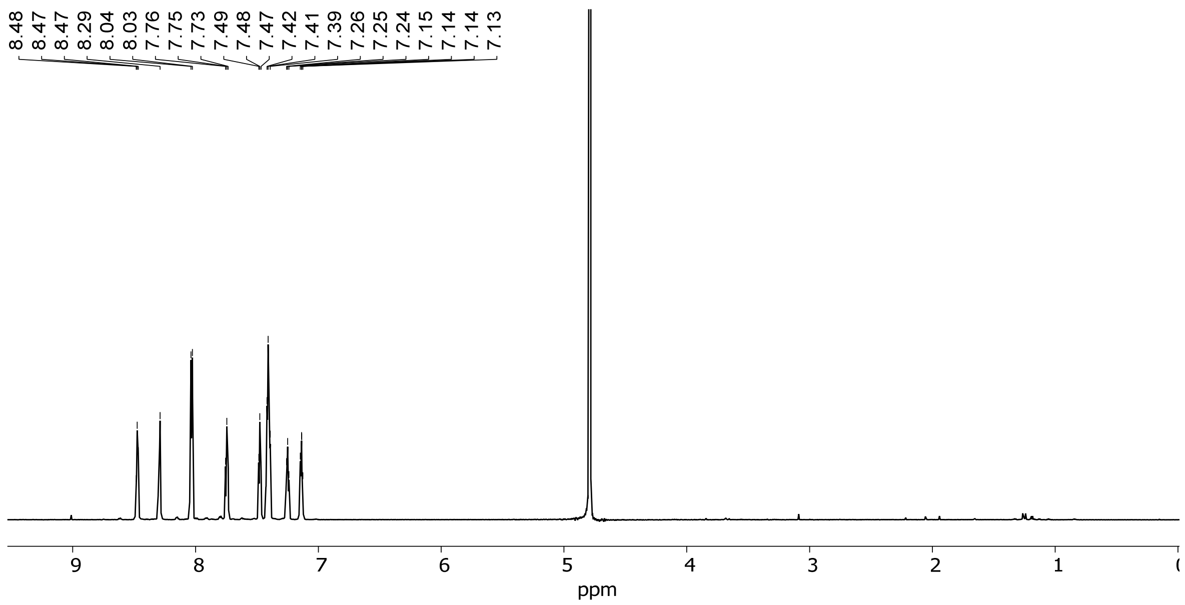

^{13}C NMR spectrum (176 MHz, D_2O) of **3r**

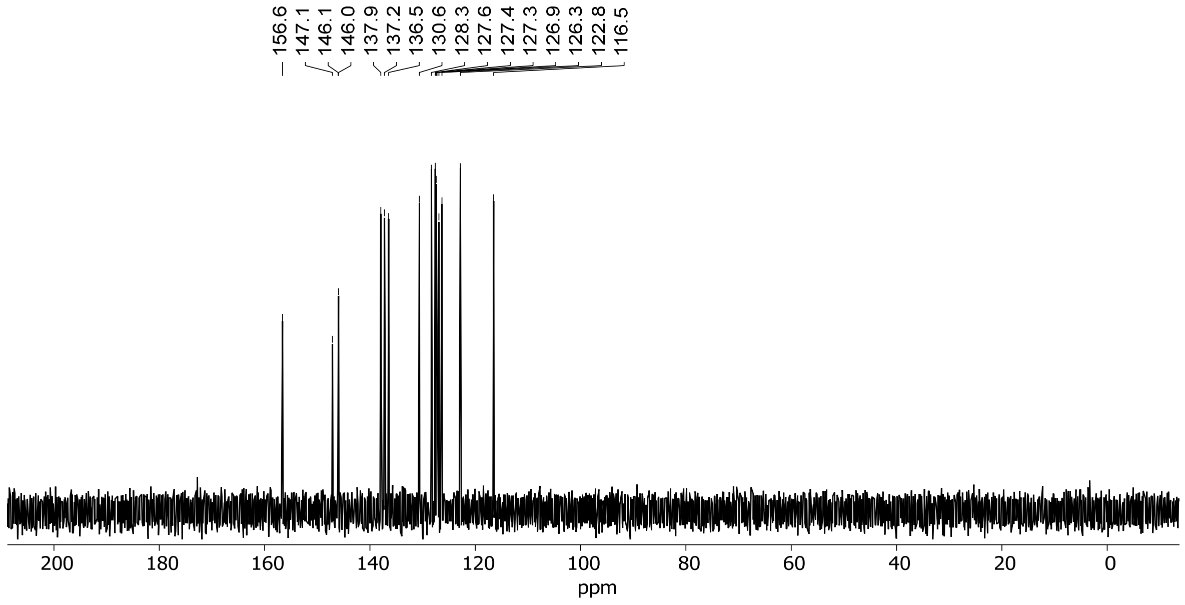

^{1}H NMR spectrum (700 MHz, C_3D_6O) of **3s**

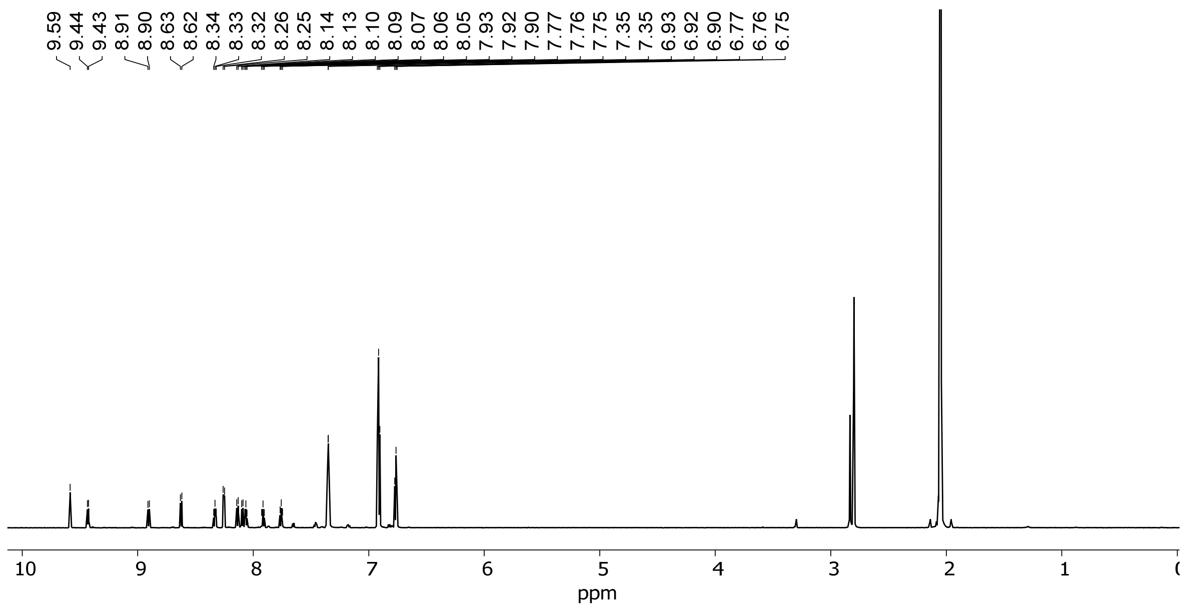

^{13}C NMR spectrum (176 MHz, C_3D_6O) of **3s**

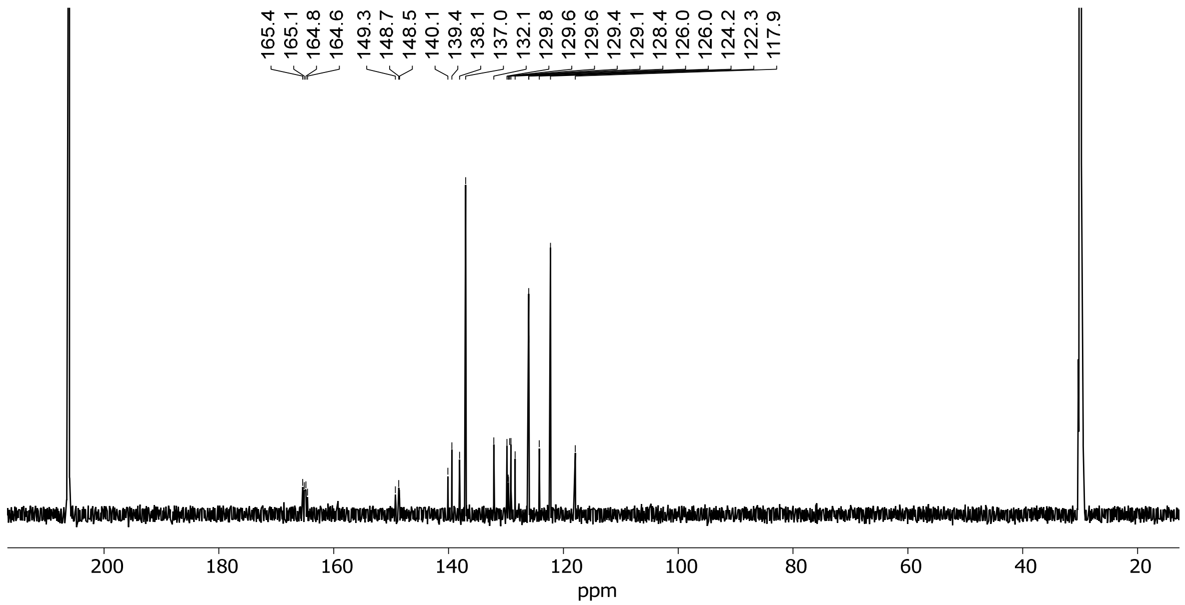

^{1}H NMR spectrum (700 MHz, DMSO-d$_6$) of **3t**

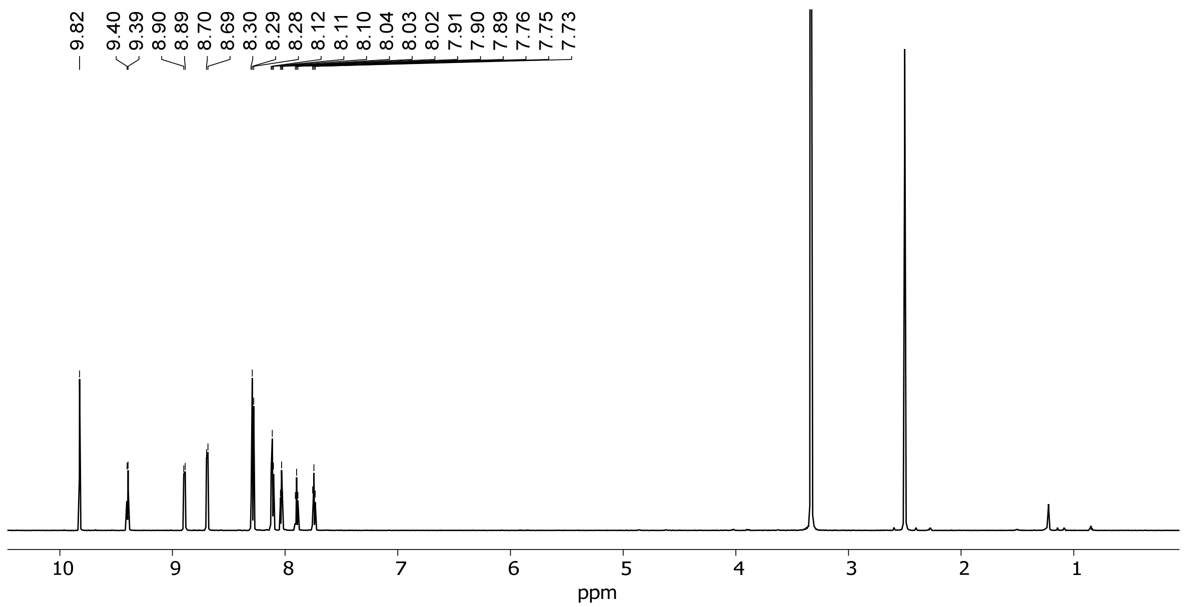

^{13}C NMR spectrum (176 MHz, DMSO-d$_6$) of **3t**

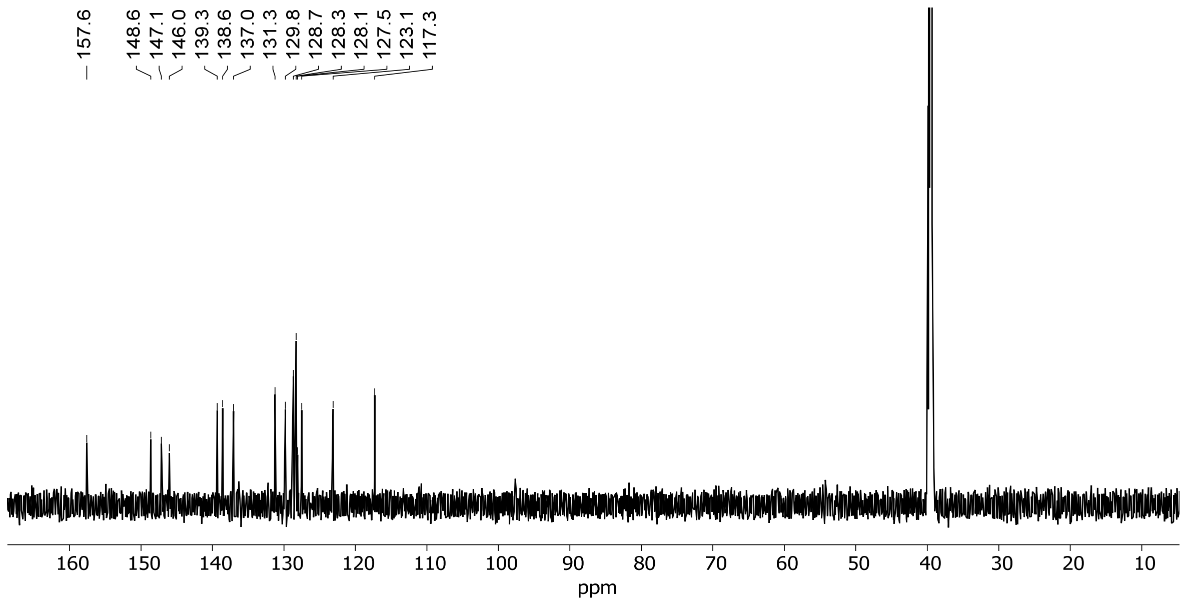

^{1}H NMR spectrum (700 MHz, D$_2$O) of **3u**

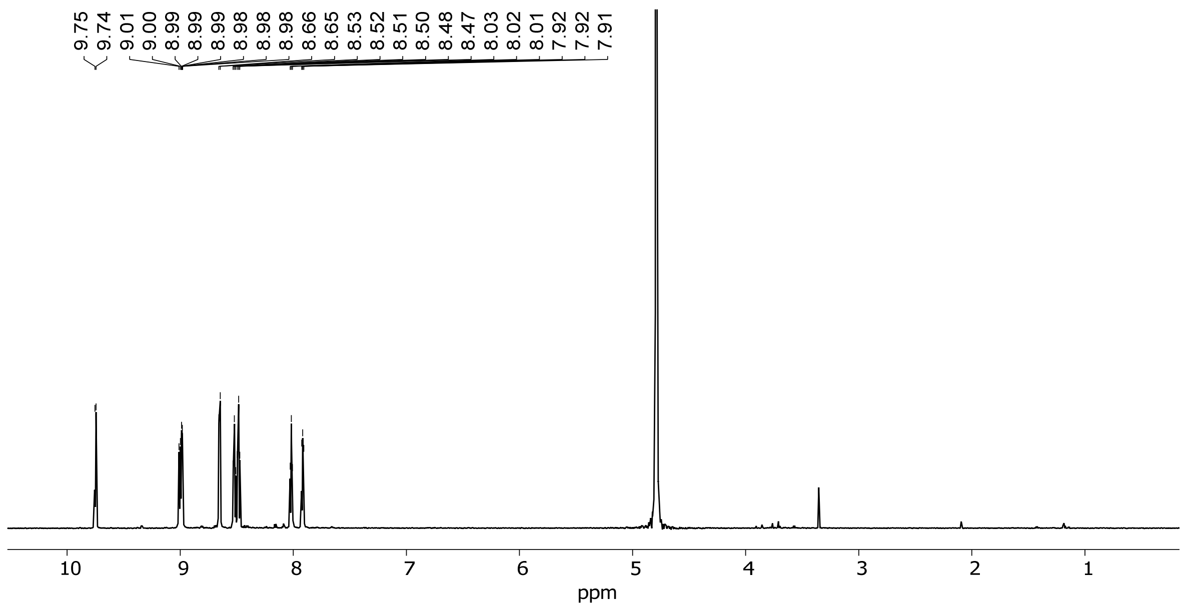

^{13}C NMR spectrum (176 MHz, D$_2$O) of **3u**

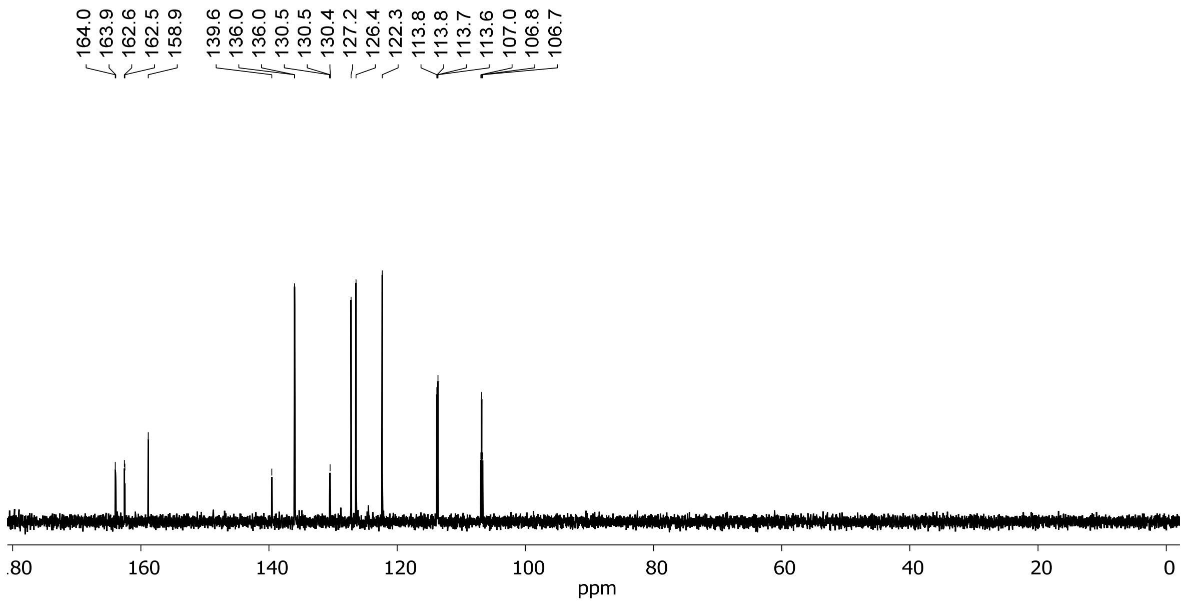

^1H NMR spectrum (700 MHz, D_2O) of **3v**

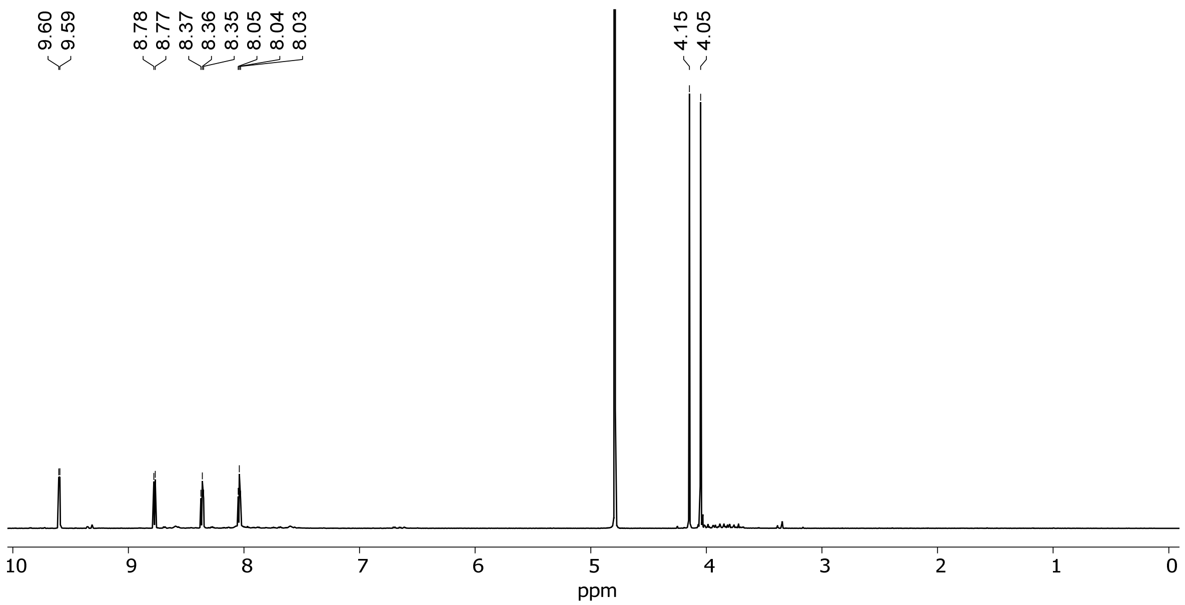

^{13}C NMR spectrum (176 MHz, D_2O) of **3v**

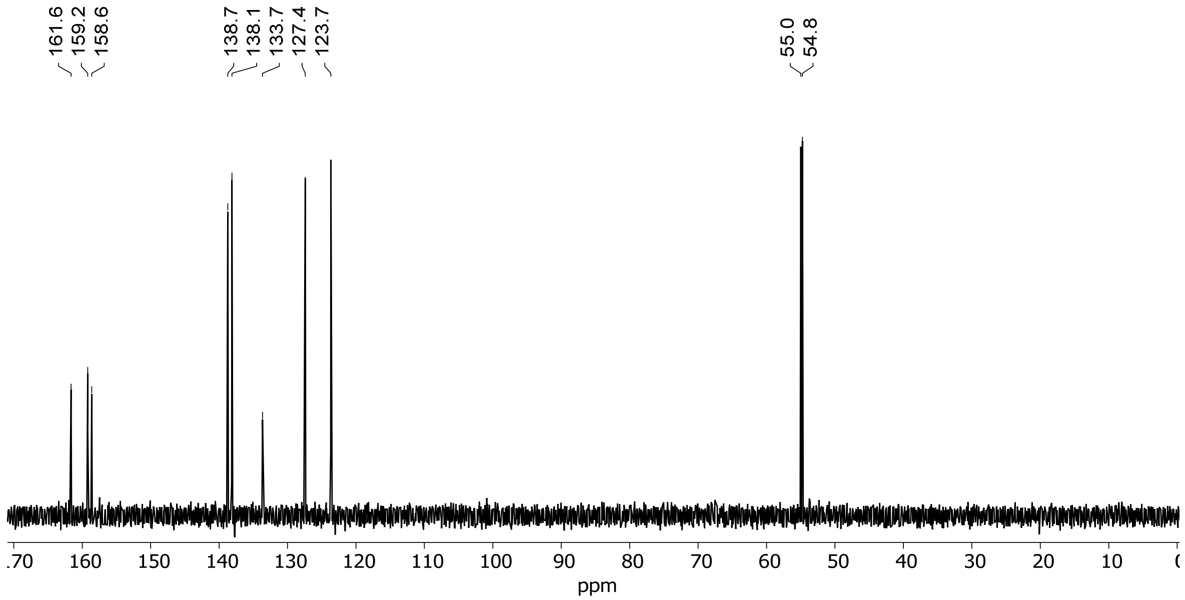

^{1}H NMR spectrum (700 MHz, C_3D_6O) of **3w**

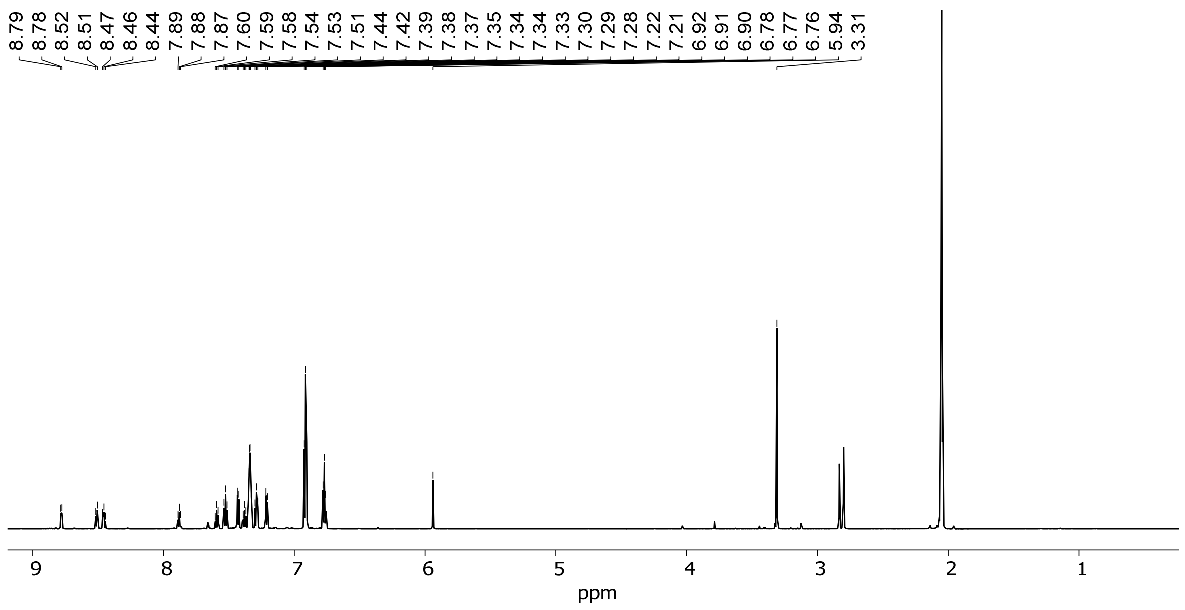

^{13}C NMR spectrum (176 MHz, C_3D_6O) of **3w**

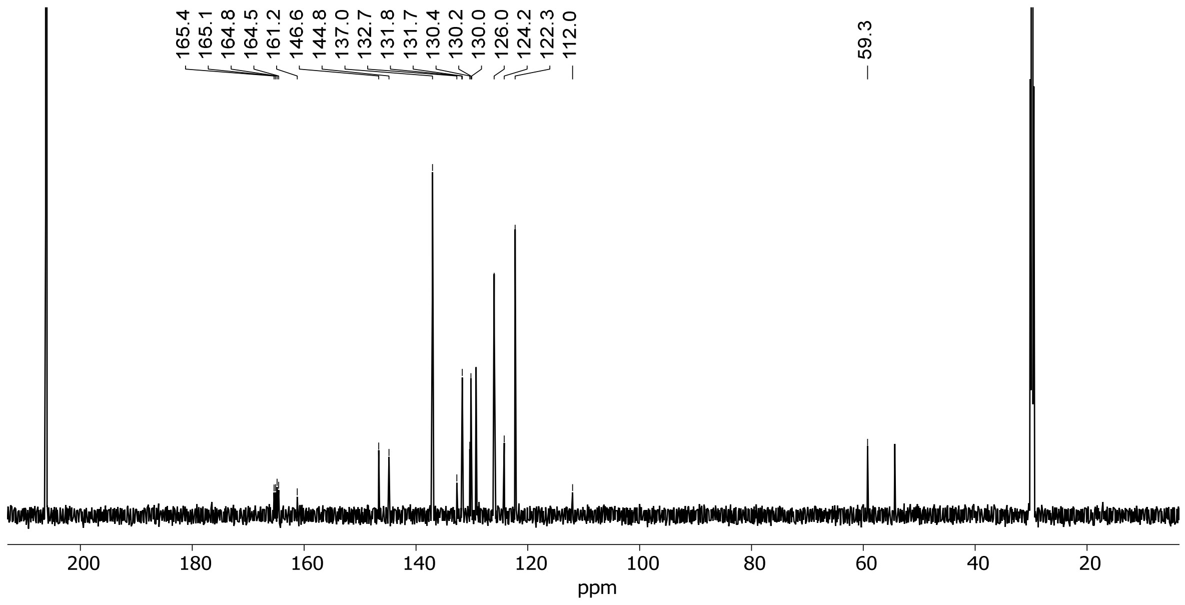

IR spectrum of **3a**

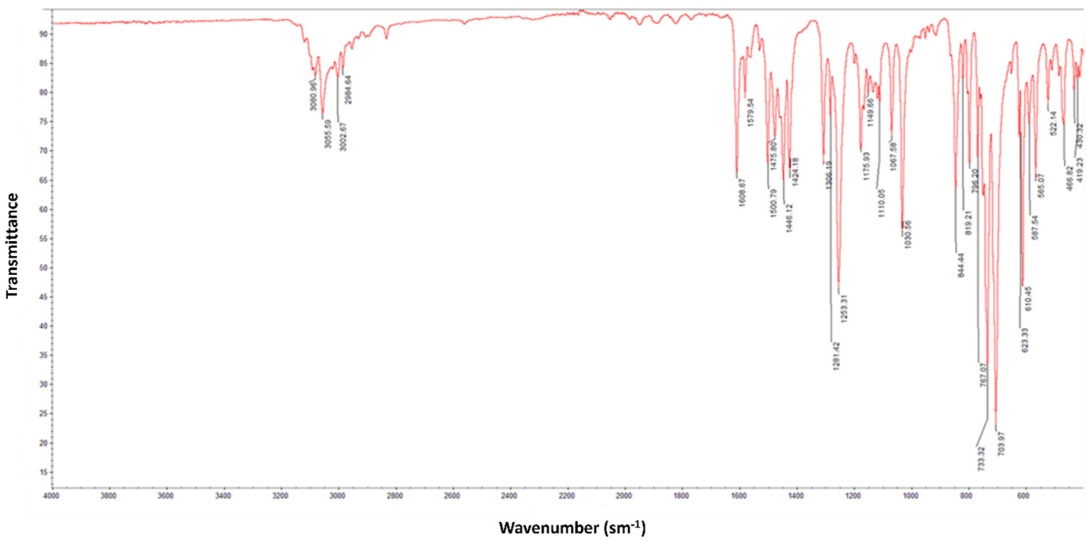

IR spectrum of **3b**

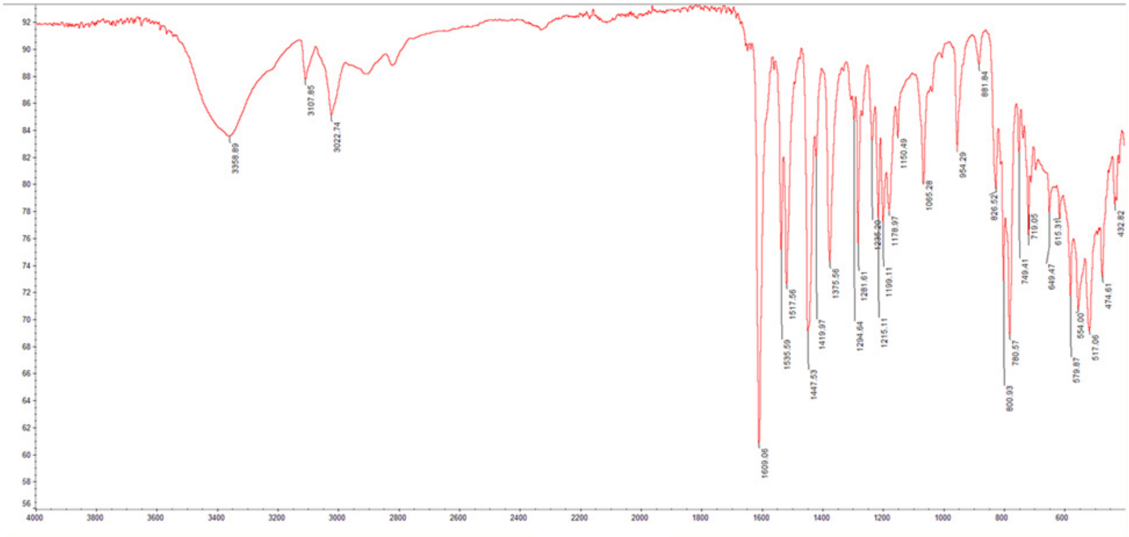

IR spectrum of **3c**

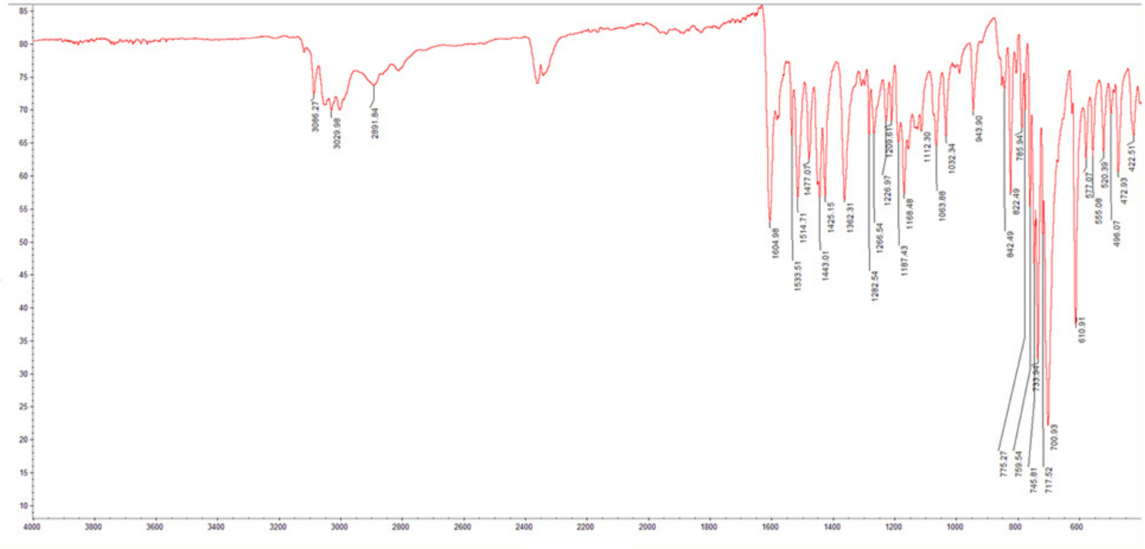

IR spectrum of **3d**

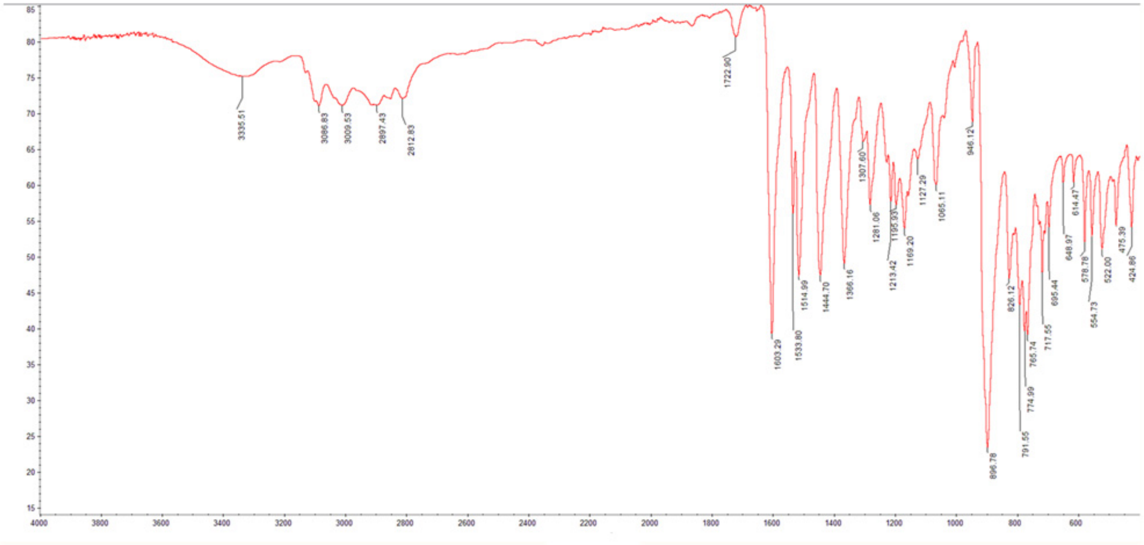

IR spectrum of **3e**

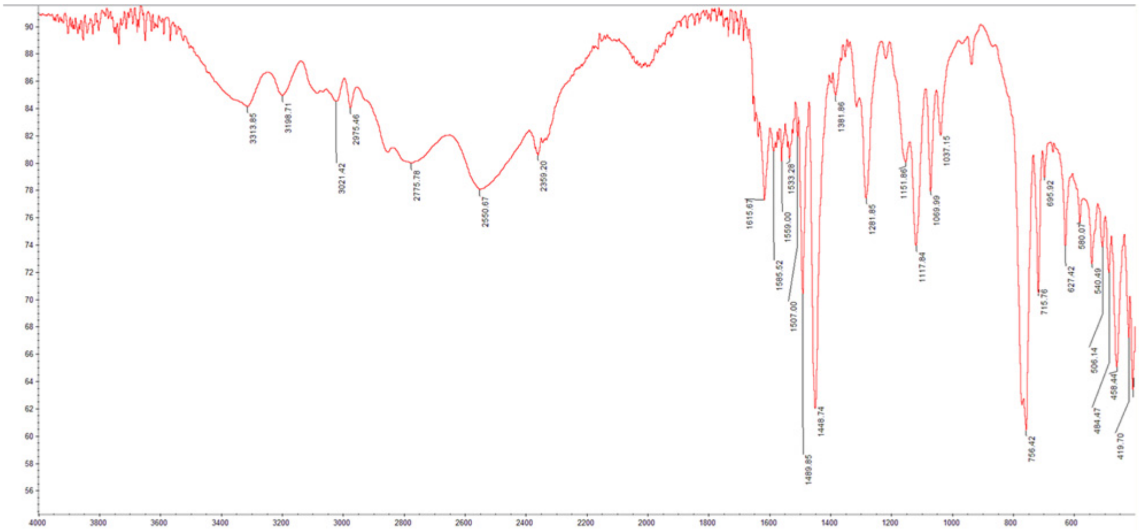

IR spectrum of **3f**

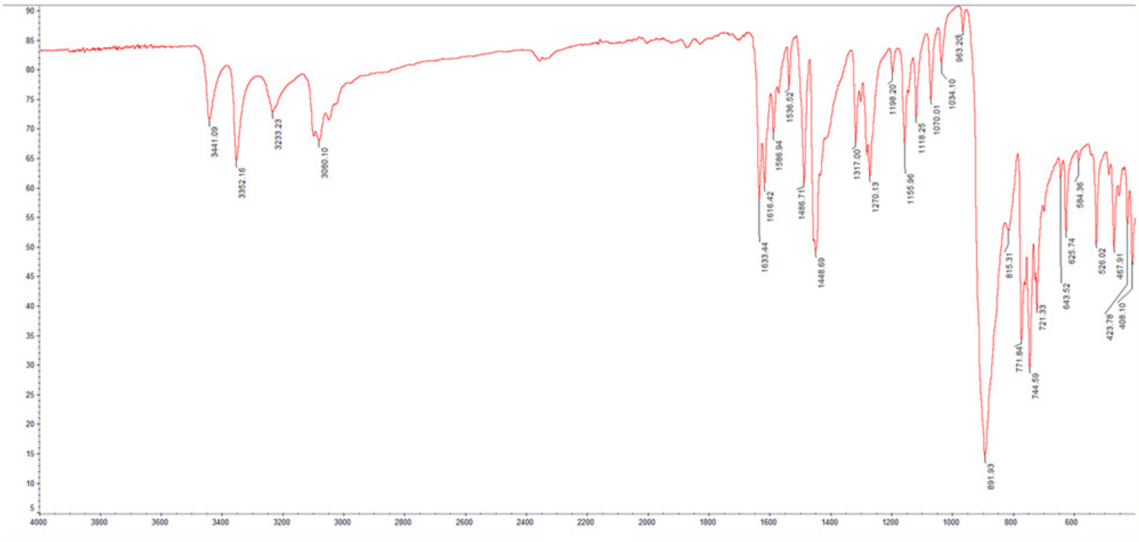

IR spectrum of **3g**

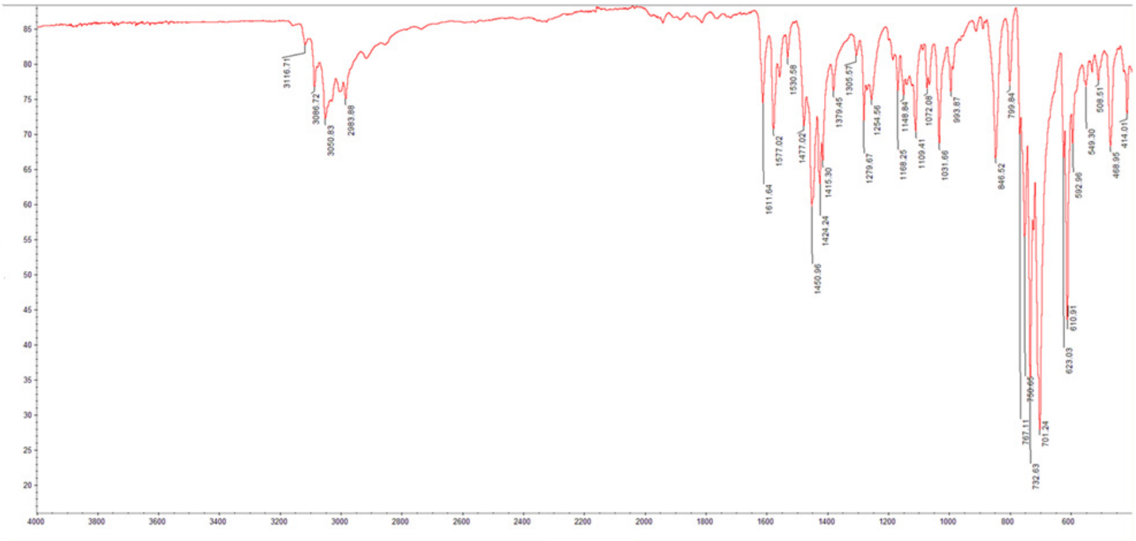

IR spectrum of **3h**

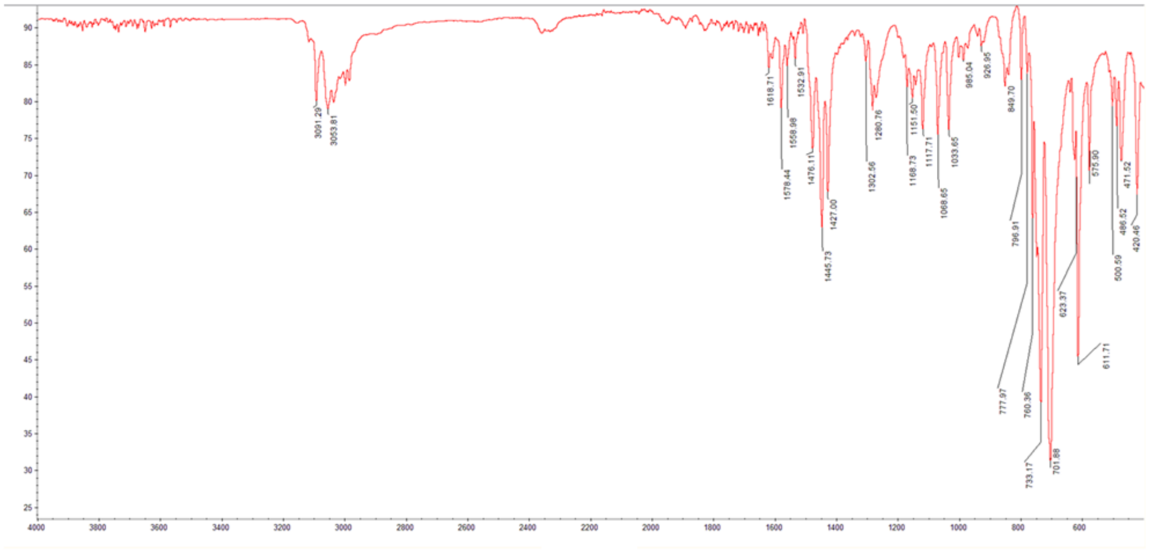

IR spectrum of **3i**

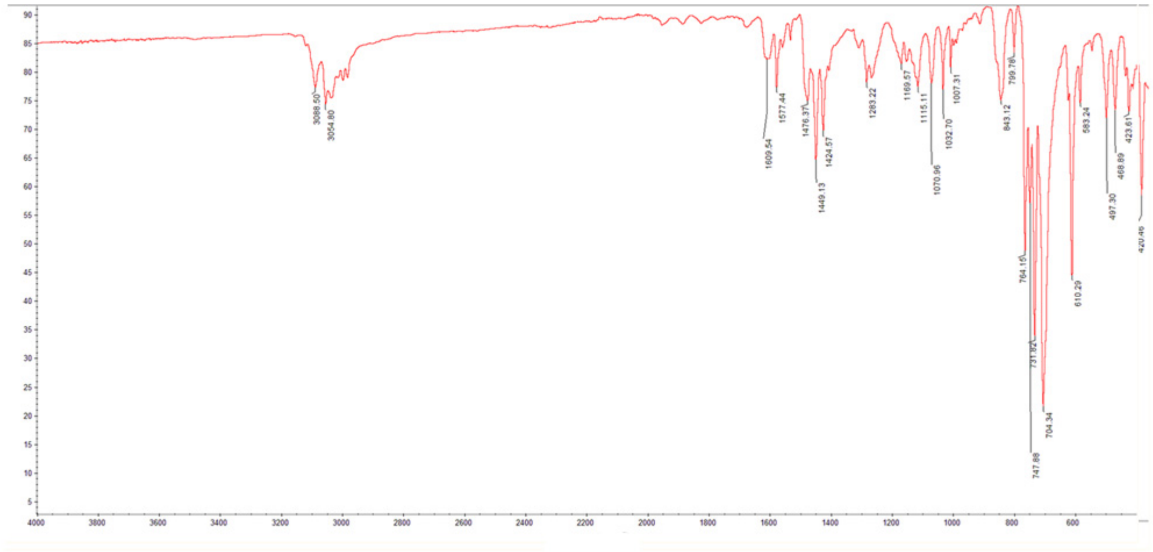

IR spectrum of **3j**

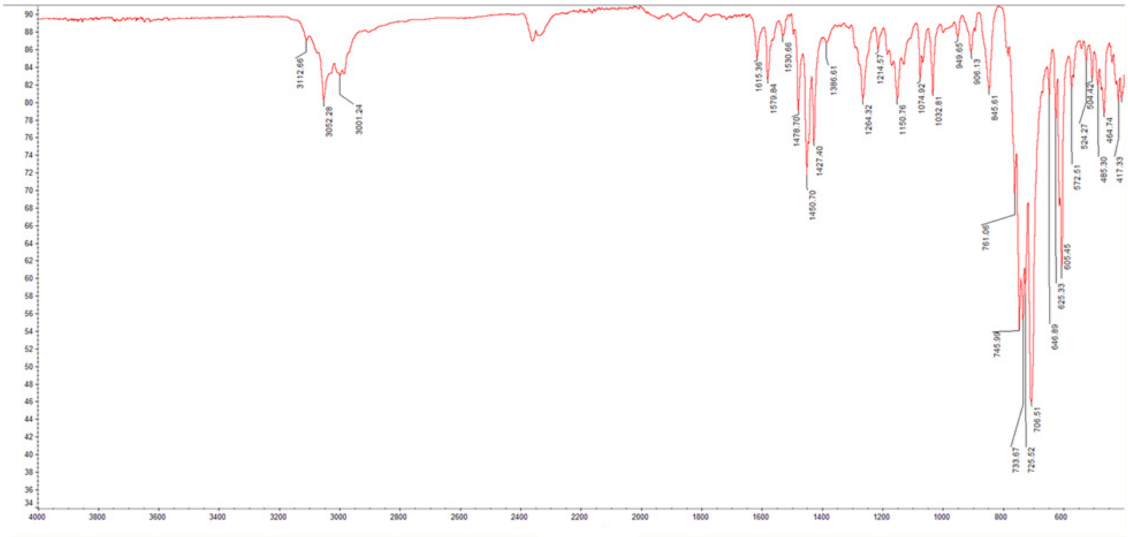

IR spectrum of **3k**

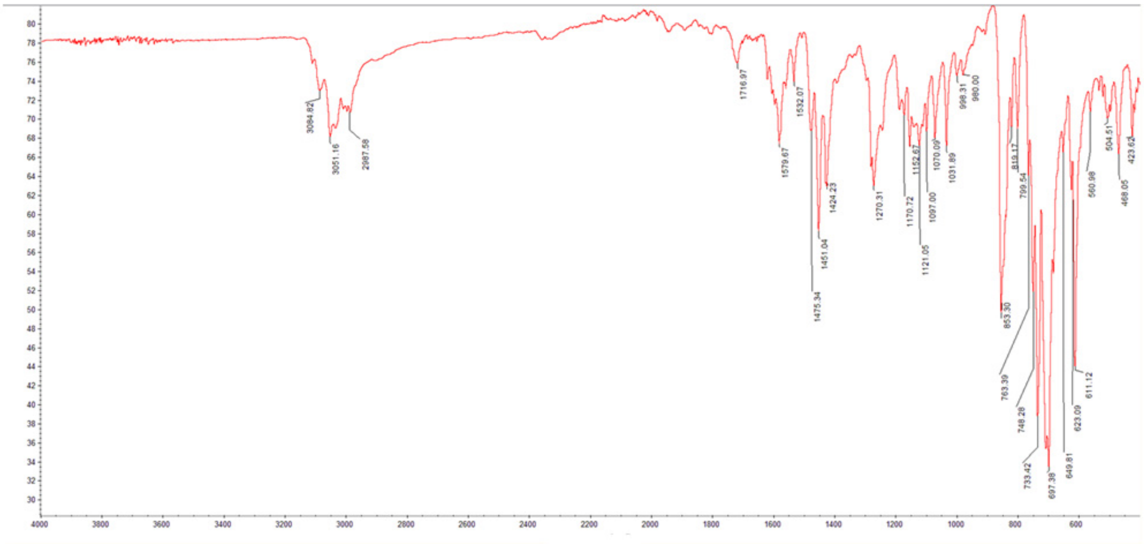

IR spectrum of **3l**

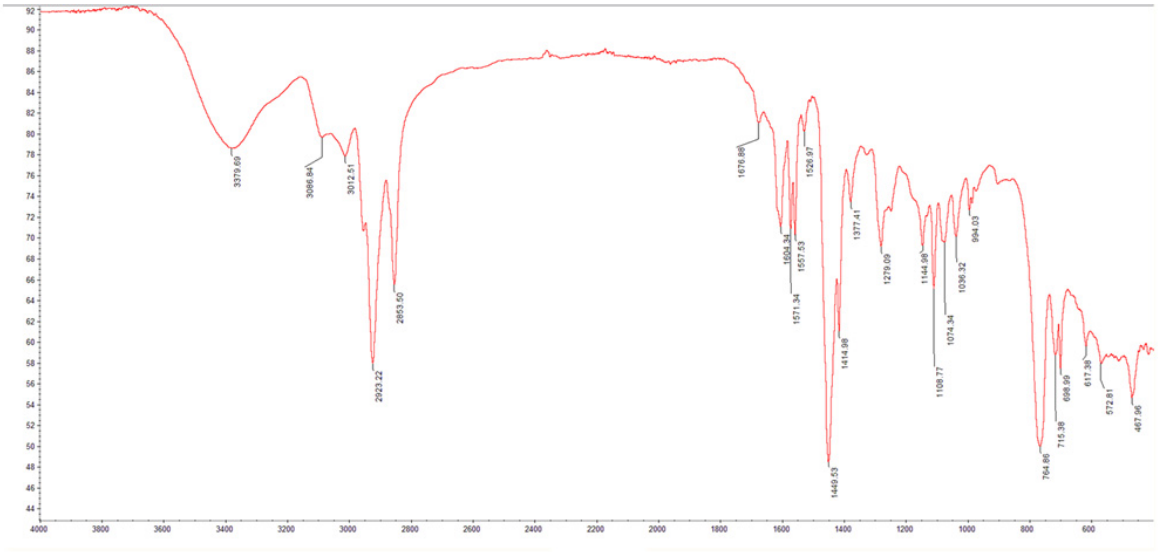

IR spectrum of **3m**

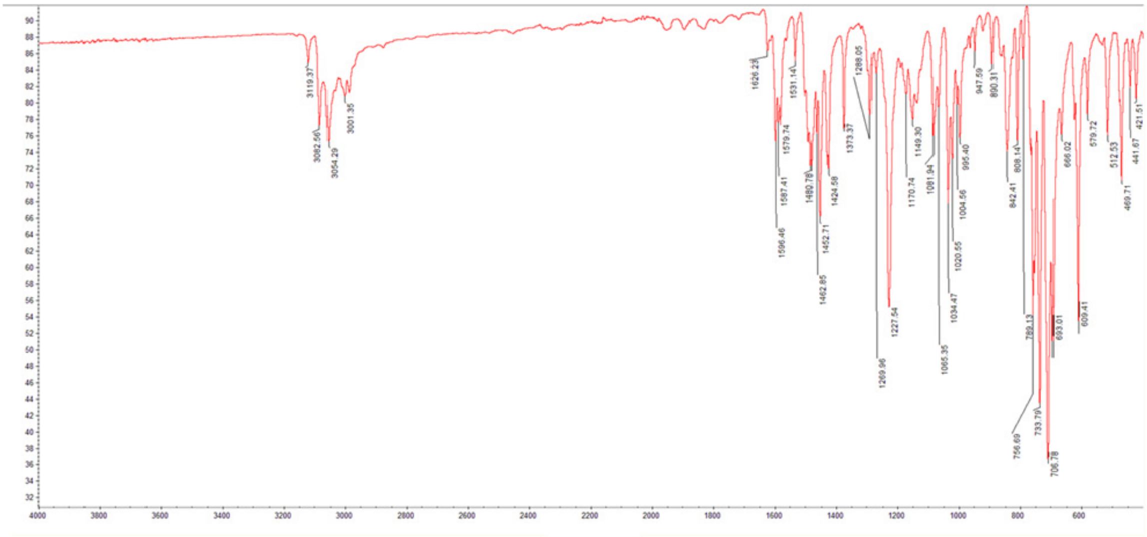

IR spectrum of **3n**

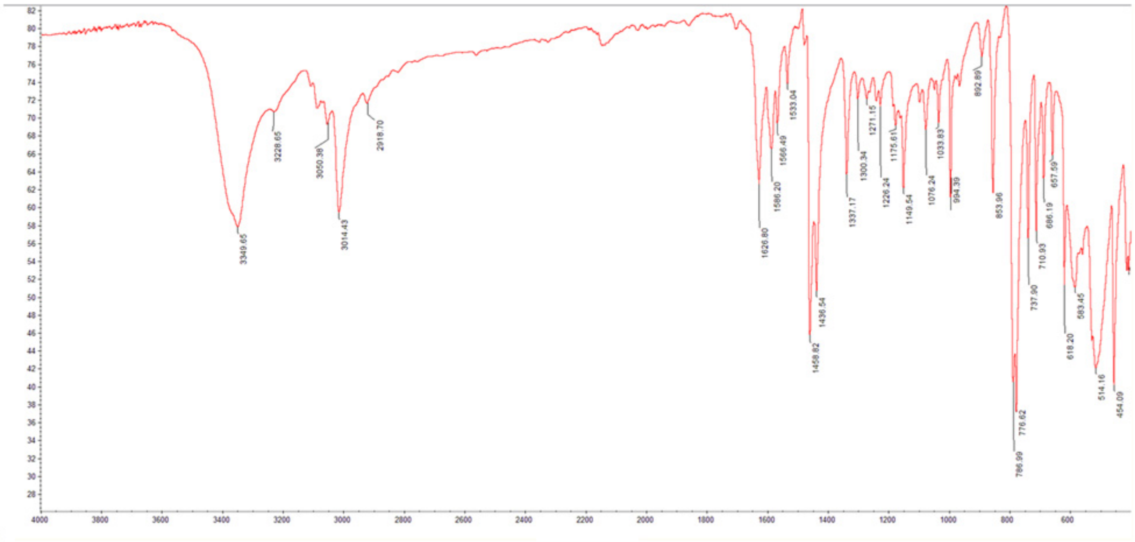

IR spectrum of **3o**

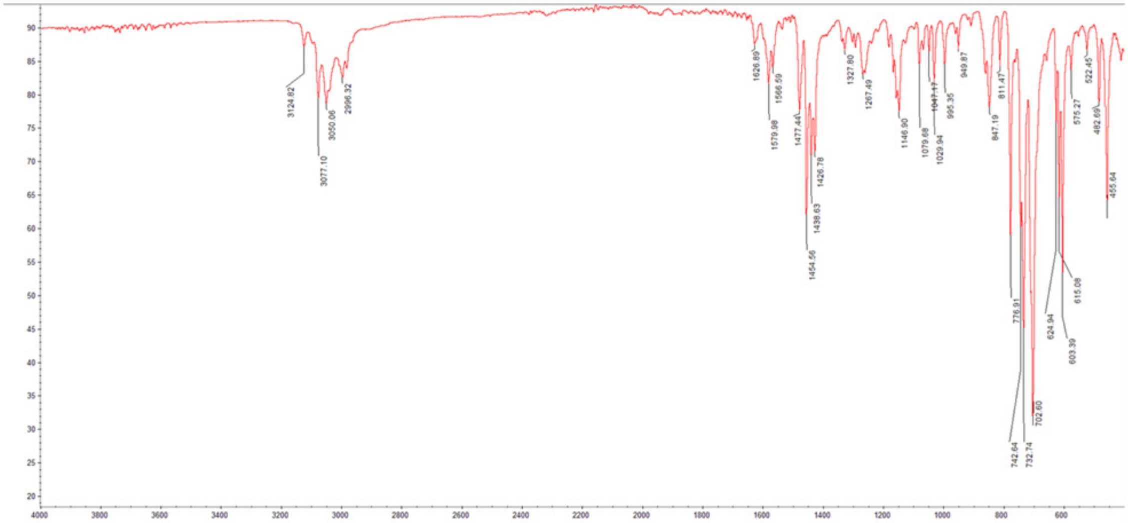

IR spectrum of **3p**

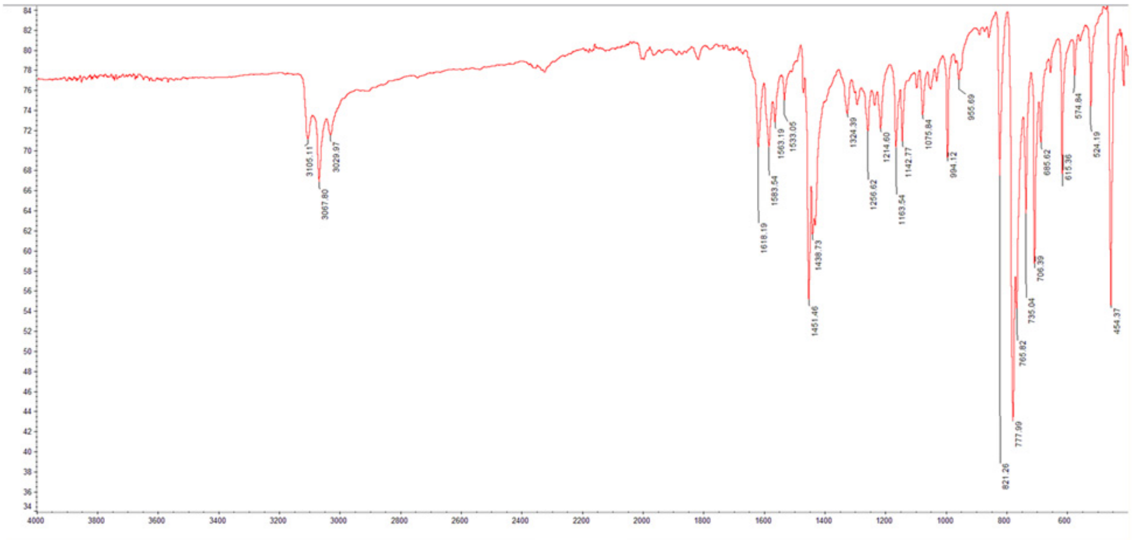

IR spectrum of **3q**

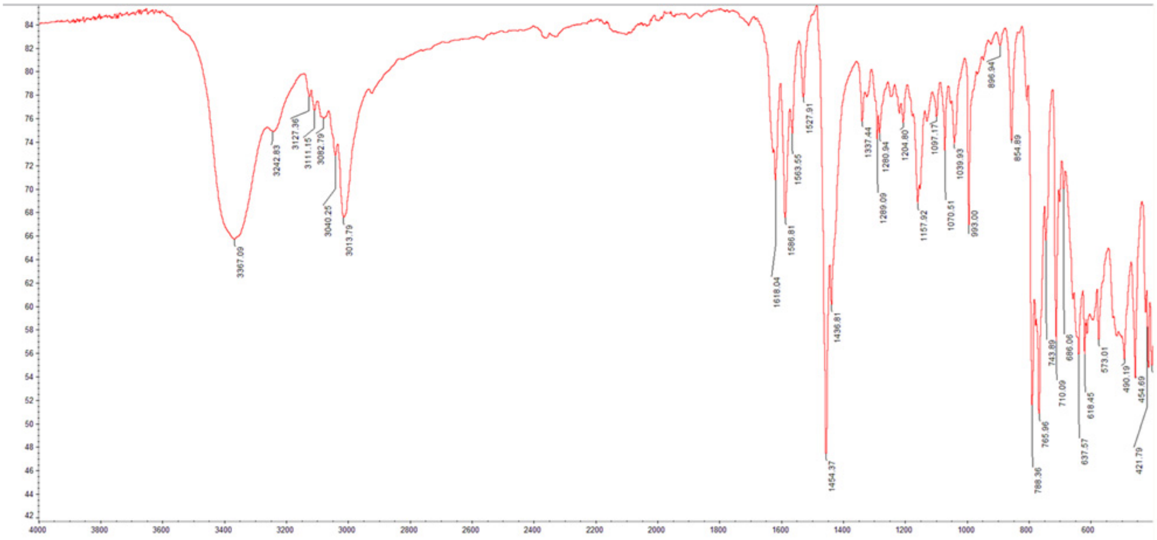

IR spectrum of **3r**

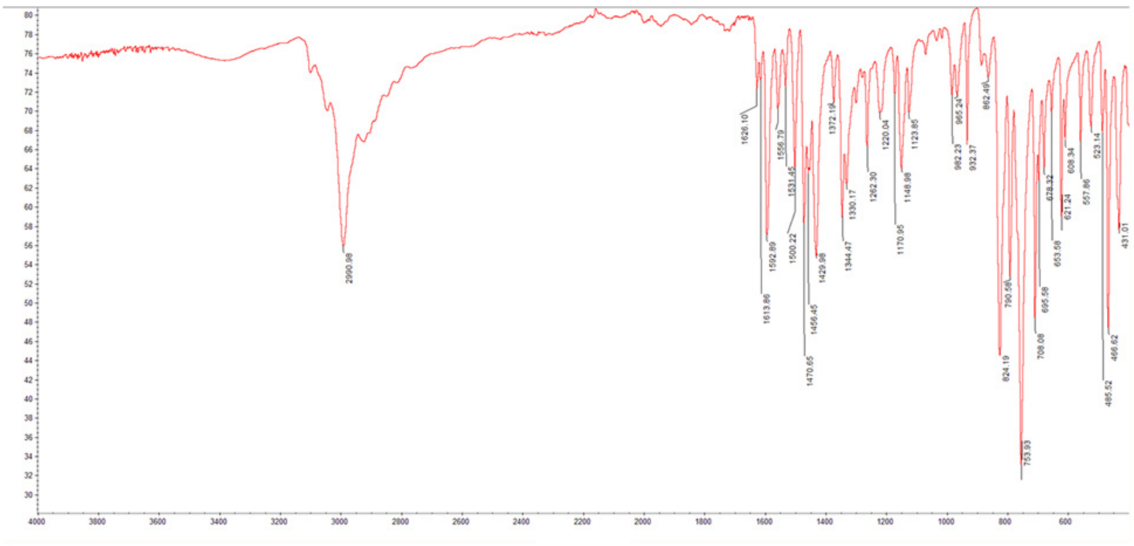

IR spectrum of **3s**

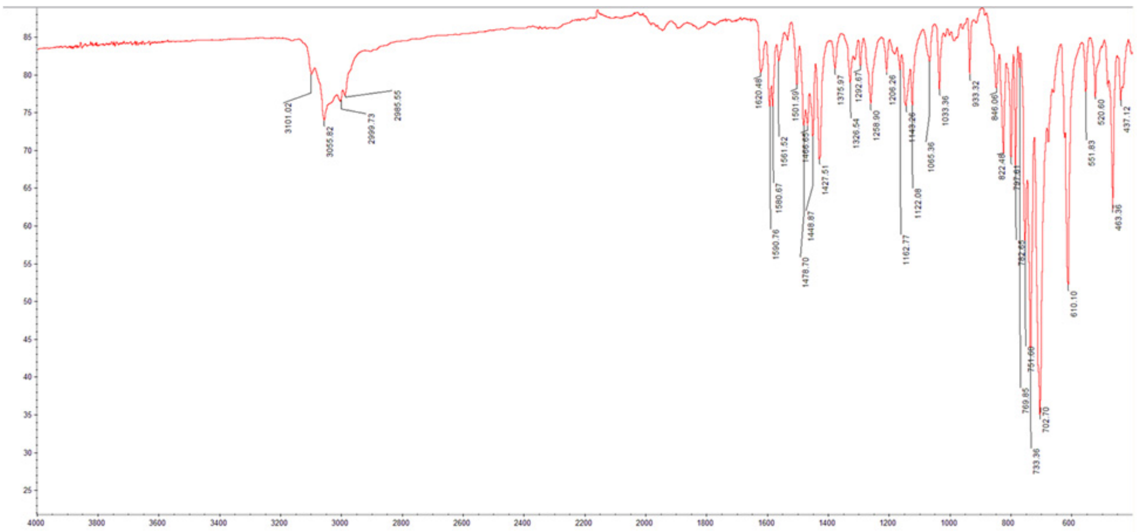

IR spectrum of **3t**

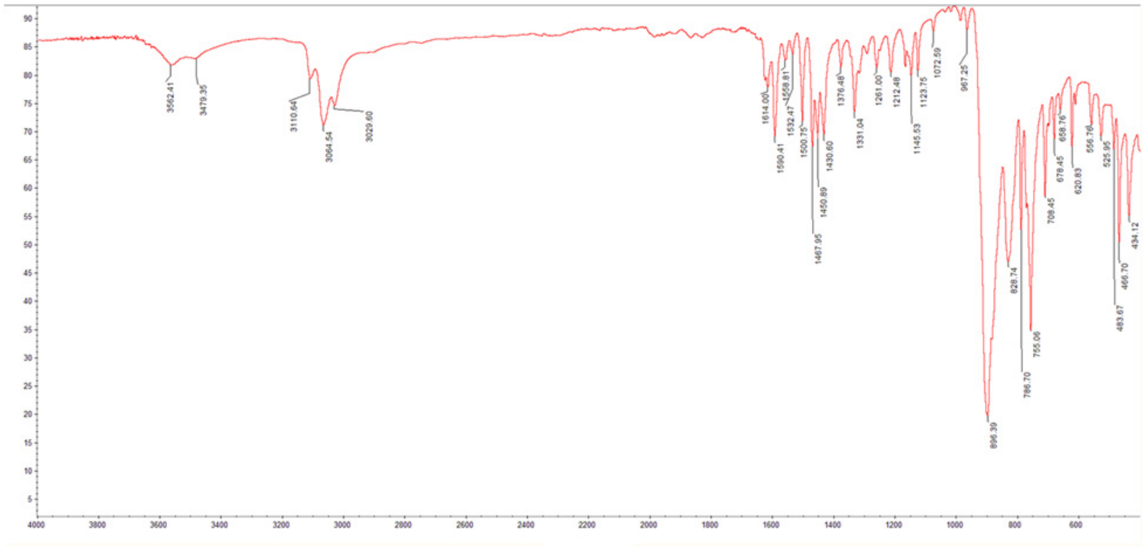

IR spectrum of **3u**

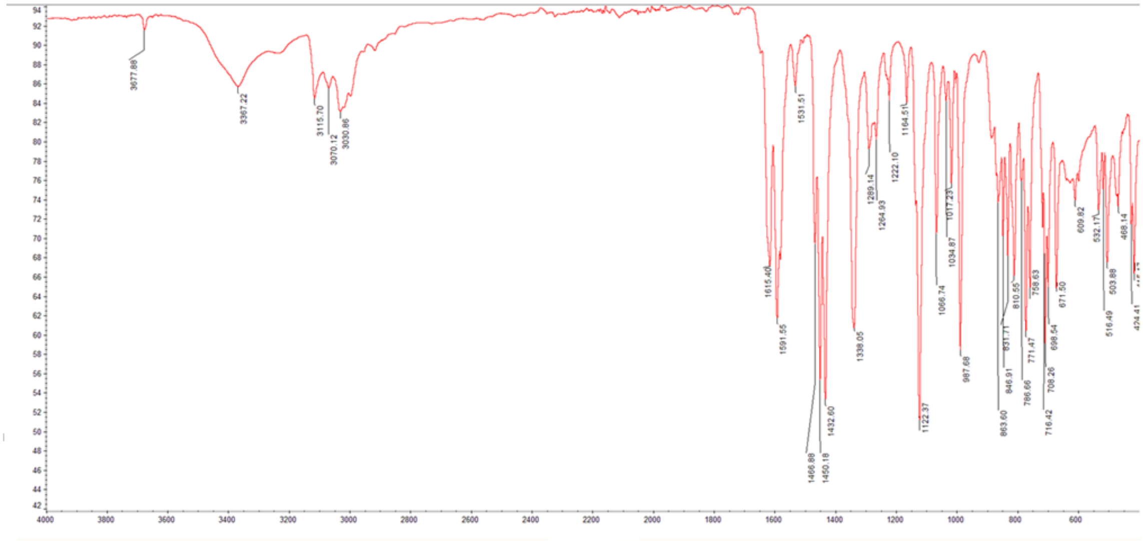

IR spectrum of **3v**

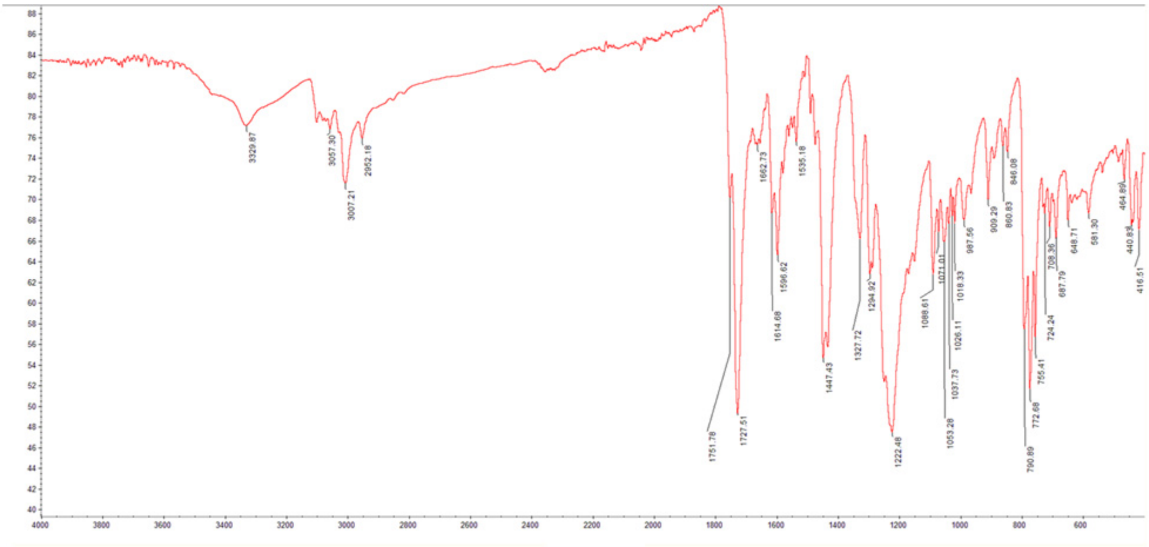

IR spectrum of **3w**

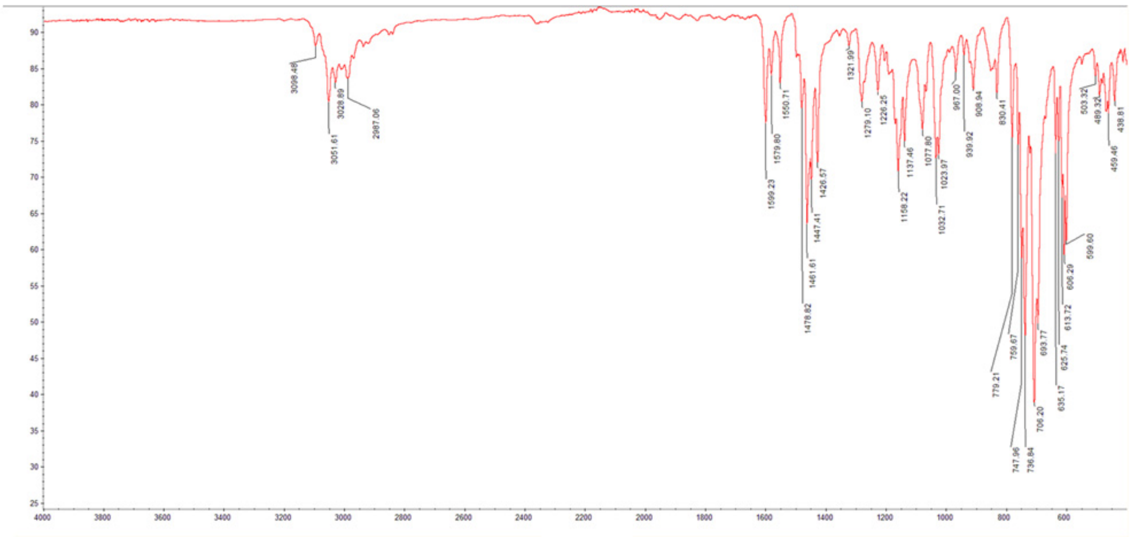

ESI HR-MS spectra of **3a**

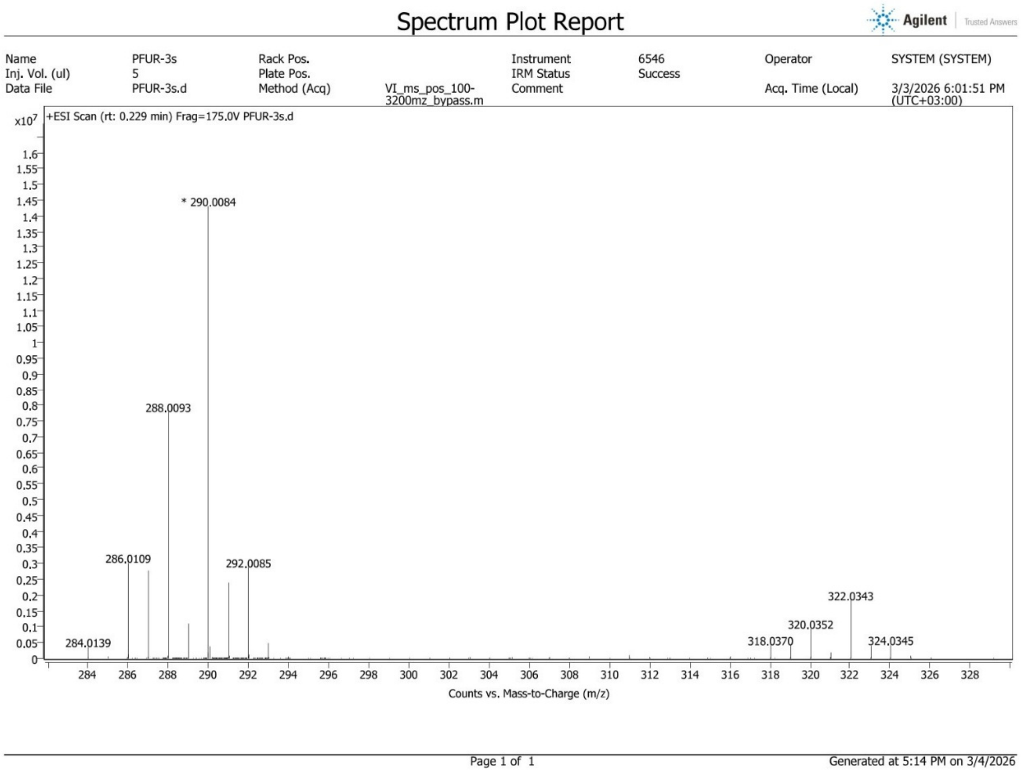

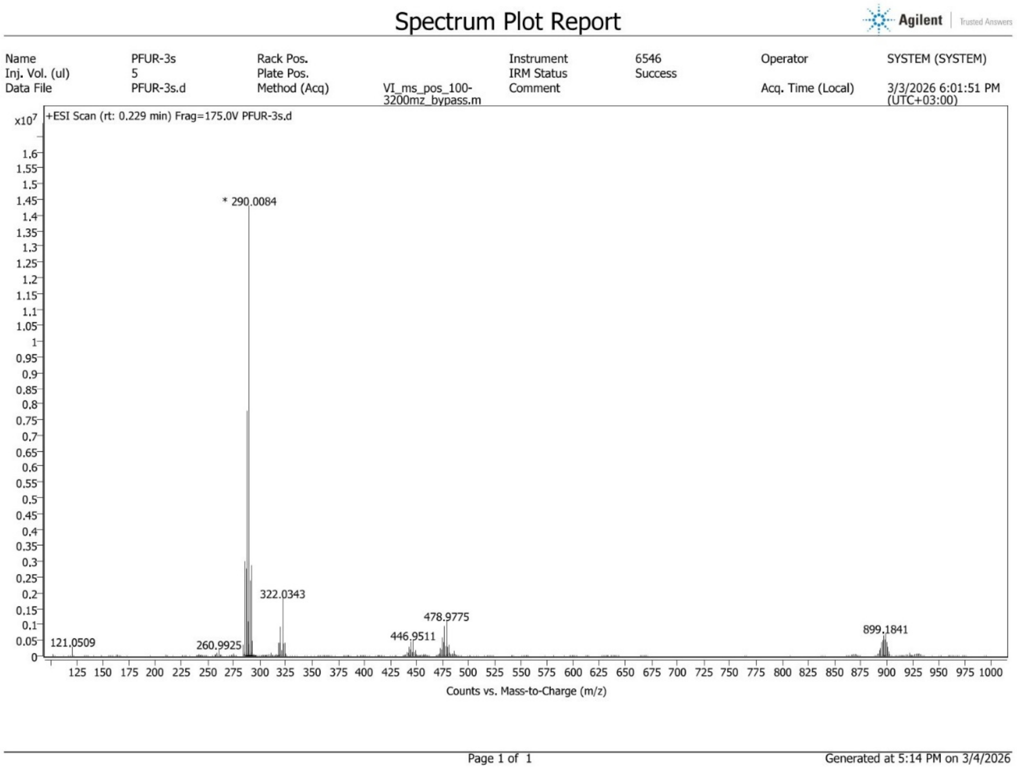

ESI HR-MS spectra of **3b**

Spectrum Plot Report

Name	PFUR-3b	Rack Pos.		Instrument	6546	Operator	SYSTEM (SYSTEM)
Inj. Vol. (ul)	5	Plate Pos.		IRM Status	Success		
Data File	PFUR-3b.d	Method (Acq)	VI_ms_pos_100-3200mz_bypass.m	Comment		Acq. Time (Local)	3/3/2026 5:18:41 PM (UTC+03:00)

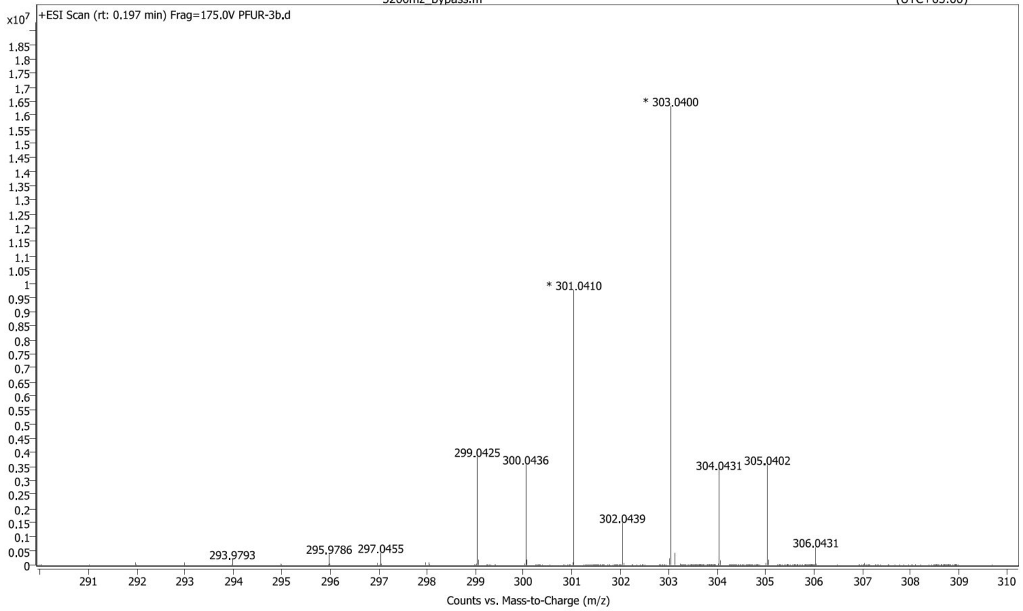

Spectrum Plot Report

Name	PFUR-3b	Rack Pos.		Instrument	6546	Operator	SYSTEM (SYSTEM)
Inj. Vol. (ul)	5	Plate Pos.		IRM Status	Success		
Data File	PFUR-3b.d	Method (Acq)	VI_ms_pos_100-3200mz_bypass.m	Comment		Acq. Time (Local)	3/3/2026 5:18:41 PM (UTC+03:00)

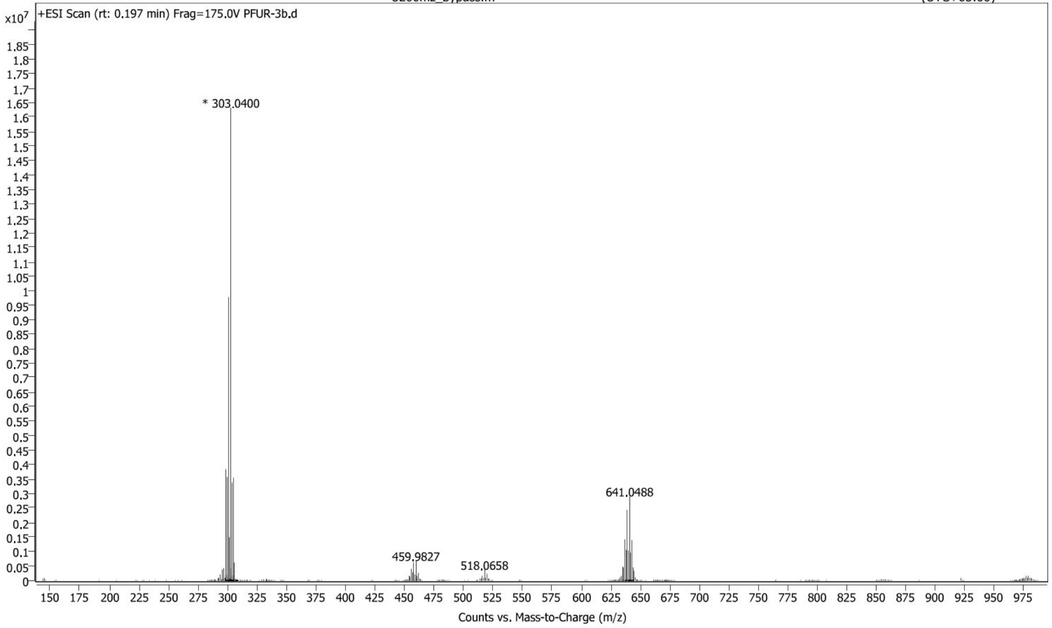

ESI HR-MS spectra of **3c**

Spectrum Plot Report

Name	PFUR-3c	Rack Pos.		Instrument	6546	Operator	SYSTEM (SYSTEM)
Inj. Vol. (ul)	5	Plate Pos.		IRM Status	Success		
Data File	PFUR-3c.d	Method (Acq)	VI_ms_pos_100-3200mz_bypass.m	Comment		Acq. Time (Local)	3/3/2026 5:21:21 PM (UTC+03:00)

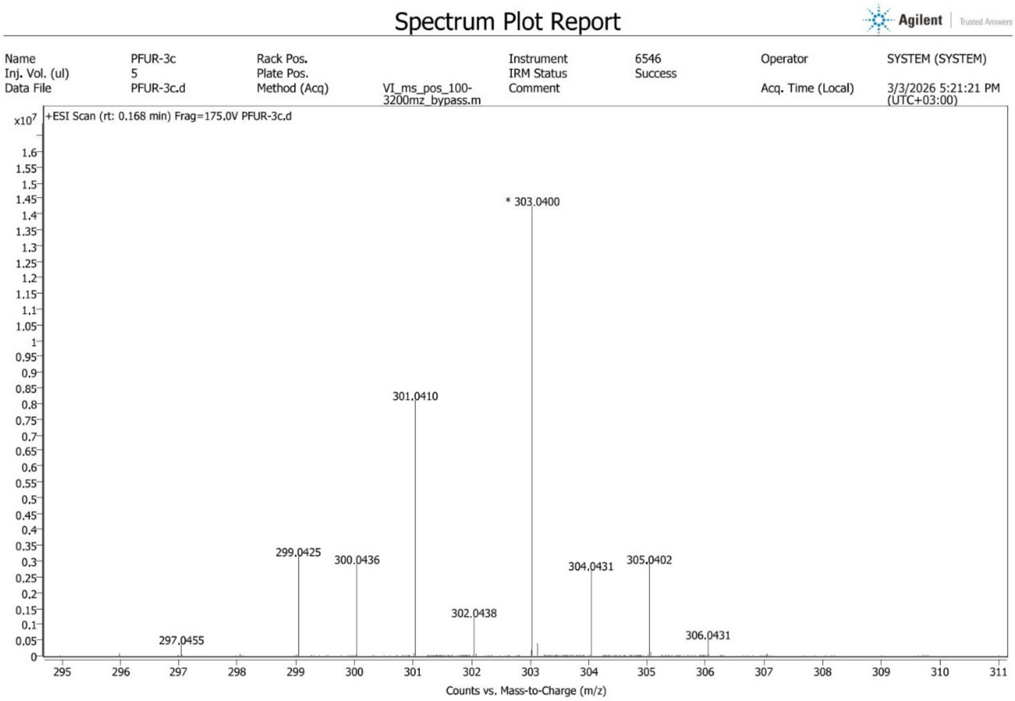

Spectrum Plot Report

Name	PFUR-3c	Rack Pos.		Instrument	6546	Operator	SYSTEM (SYSTEM)
Inj. Vol. (ul)	5	Plate Pos.		IRM Status	Success		
Data File	PFUR-3c.d	Method (Acq)	VI_ms_pos_100-3200mz_bypass.m	Comment		Acq. Time (Local)	3/3/2026 5:21:21 PM (UTC+03:00)

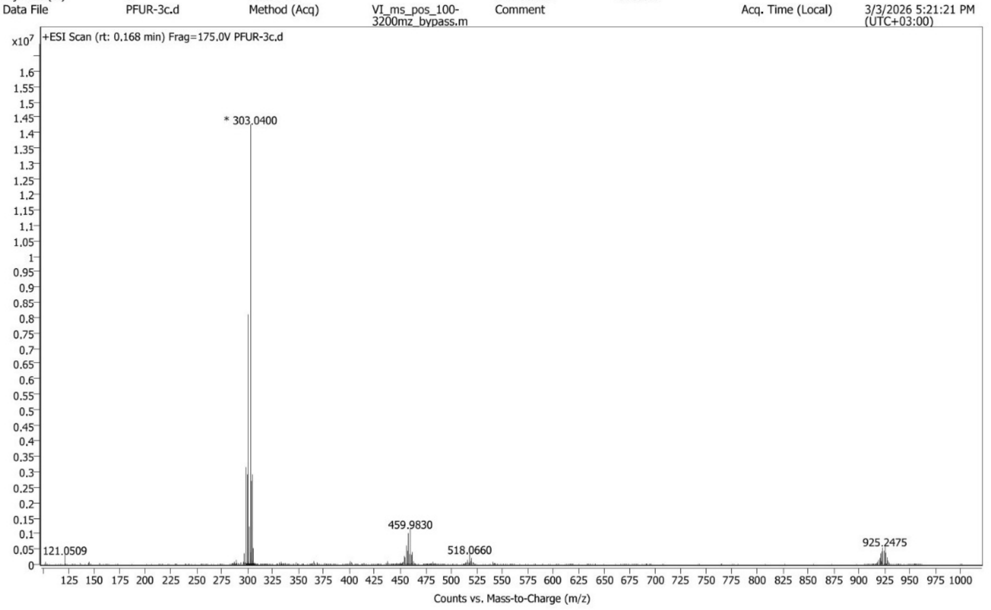

ESI HR-MS spectra of **3d**

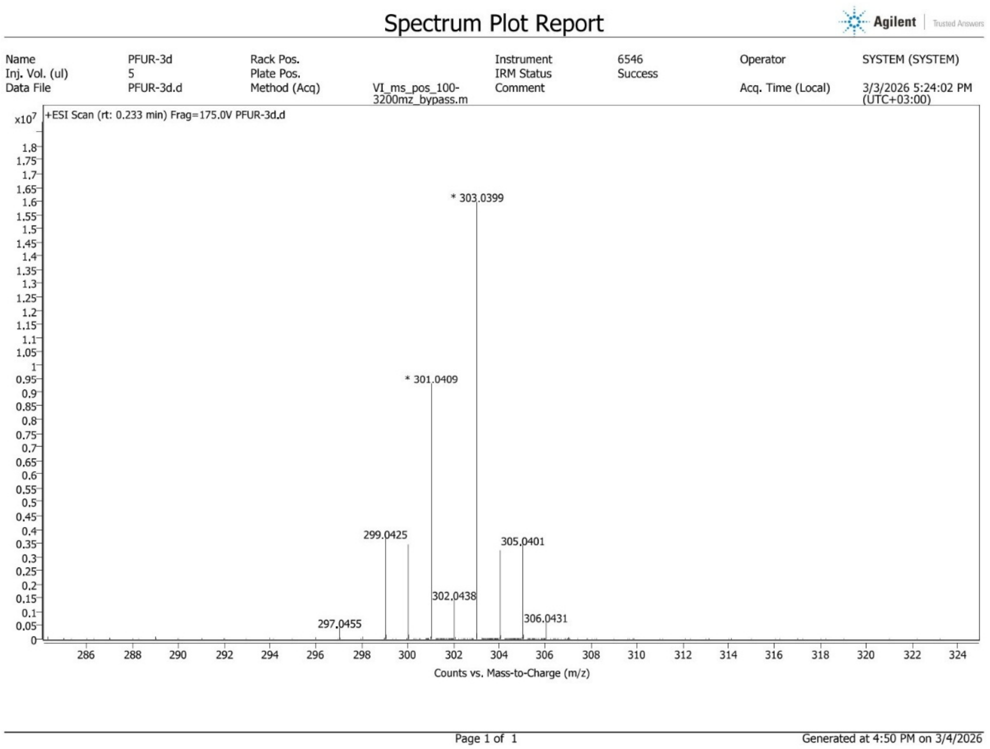

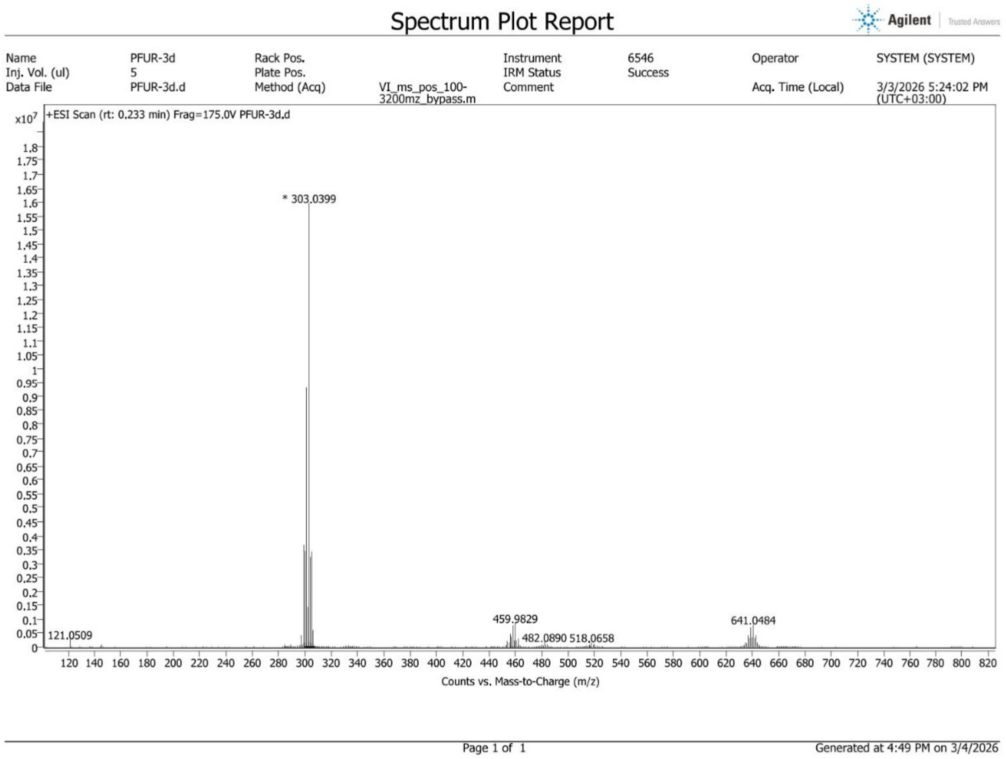

ESI HR-MS spectra of **3e**

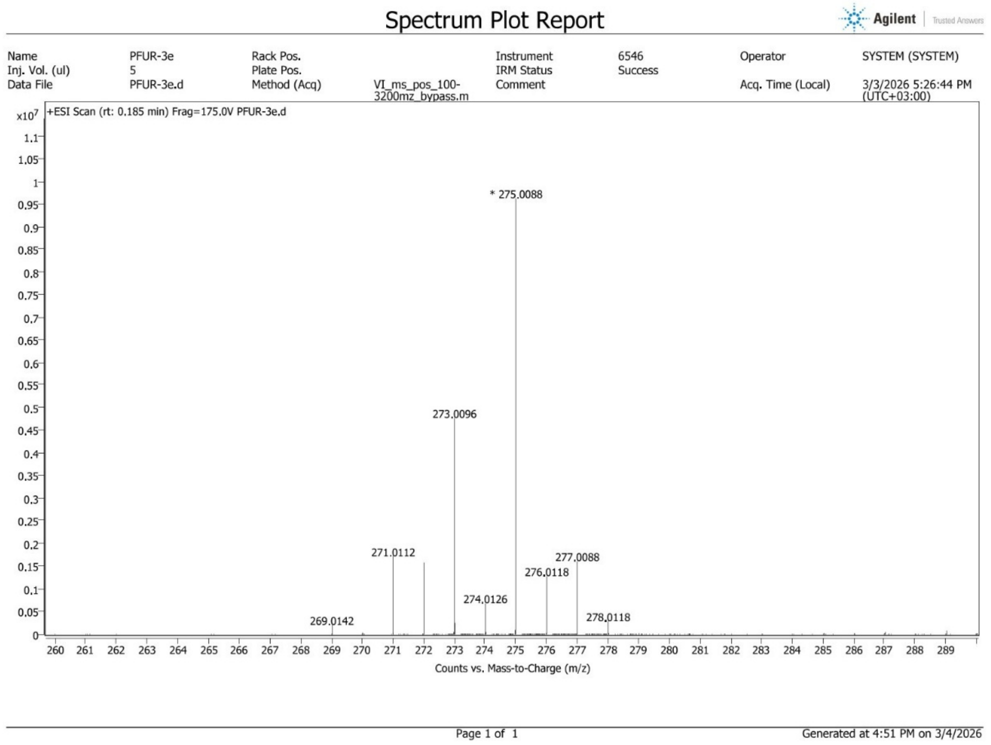

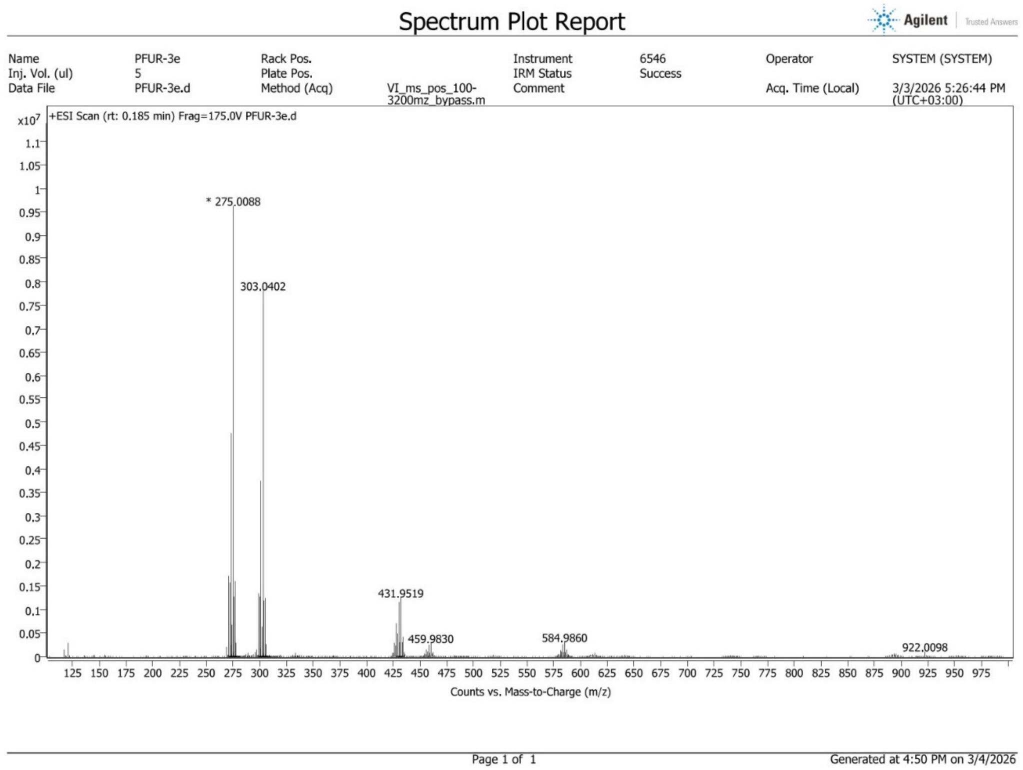

ESI HR-MS spectra of **3f**

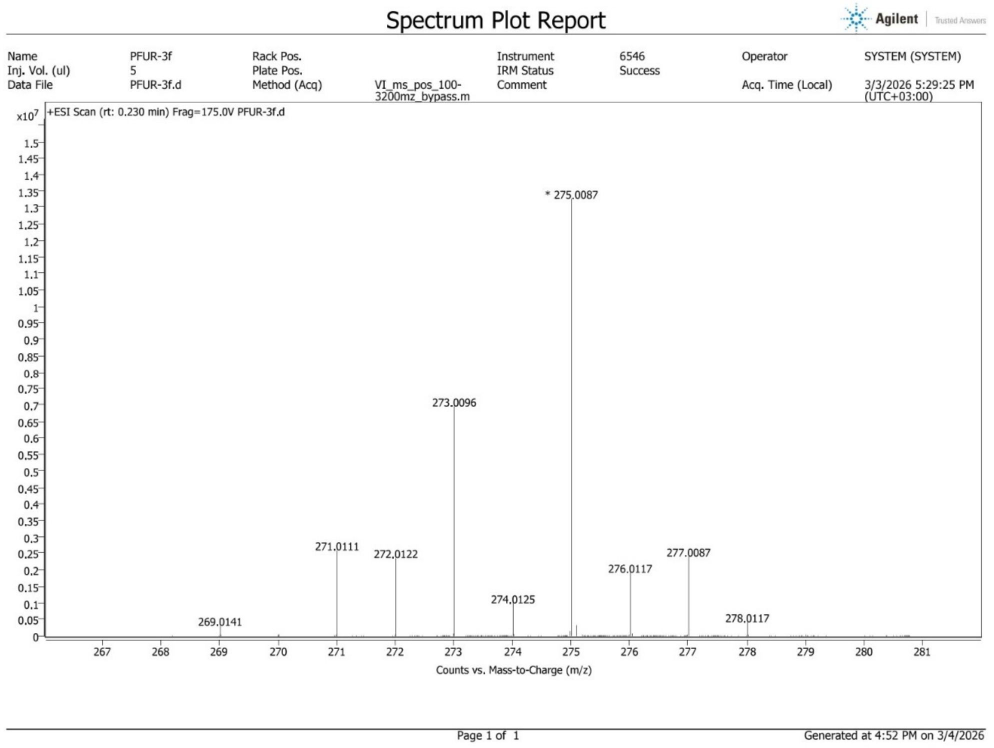

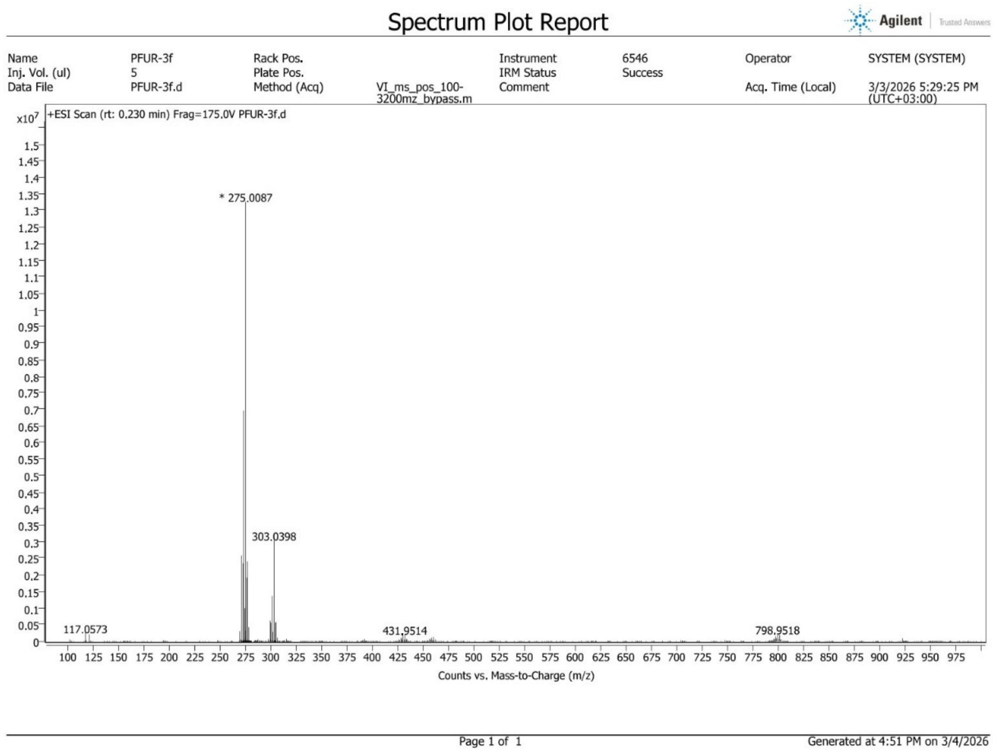

ESI HR-MS spectra of **3g**

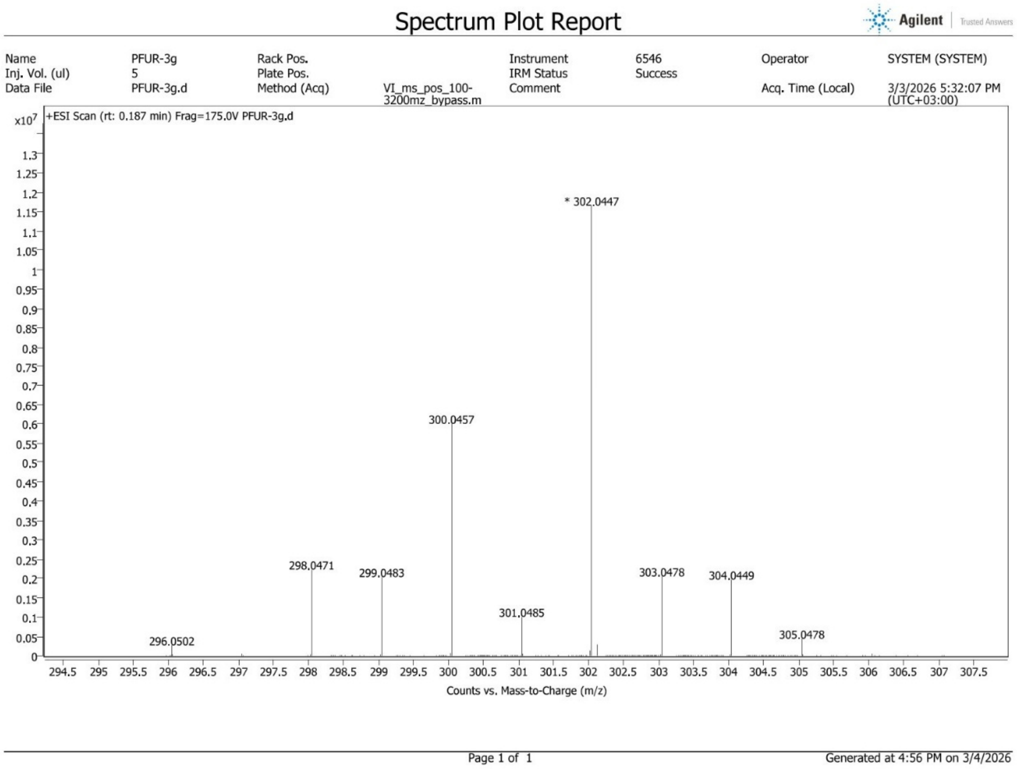

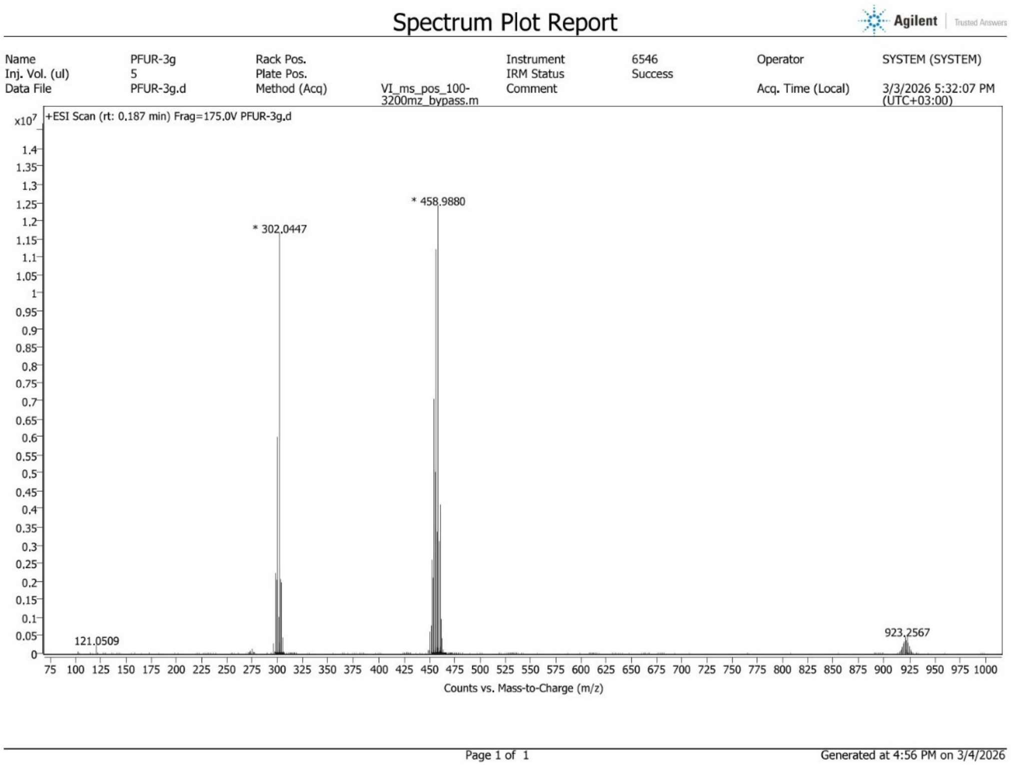

ESI HR-MS spectra of **3h**

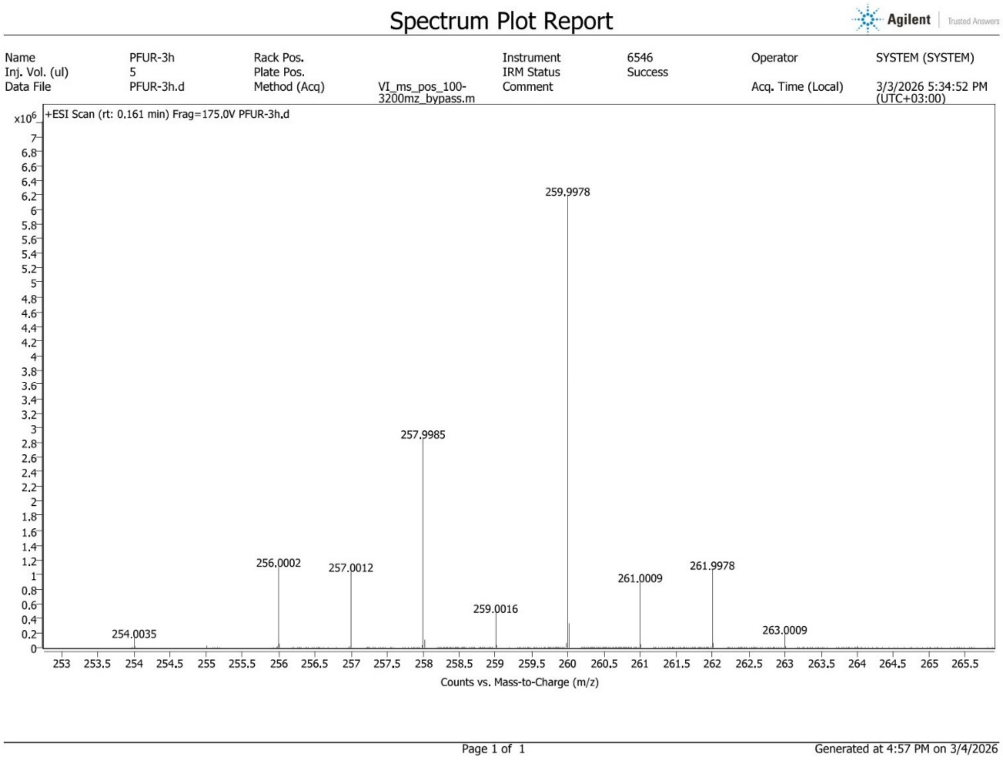

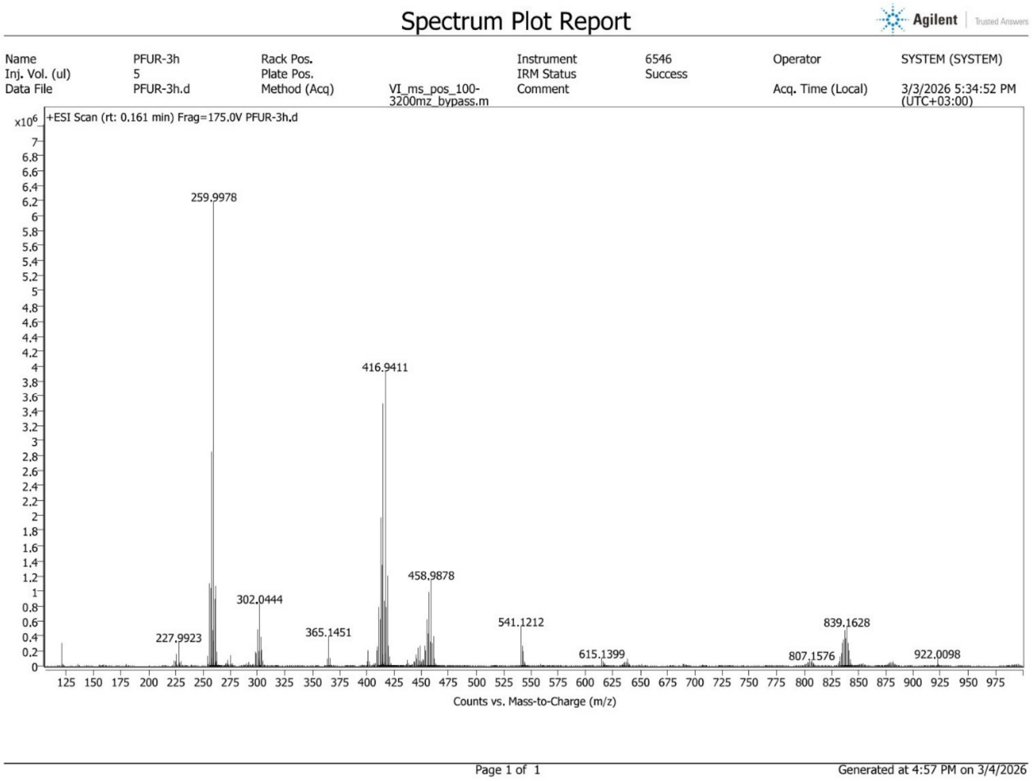

ESI HR-MS spectra of **3i**

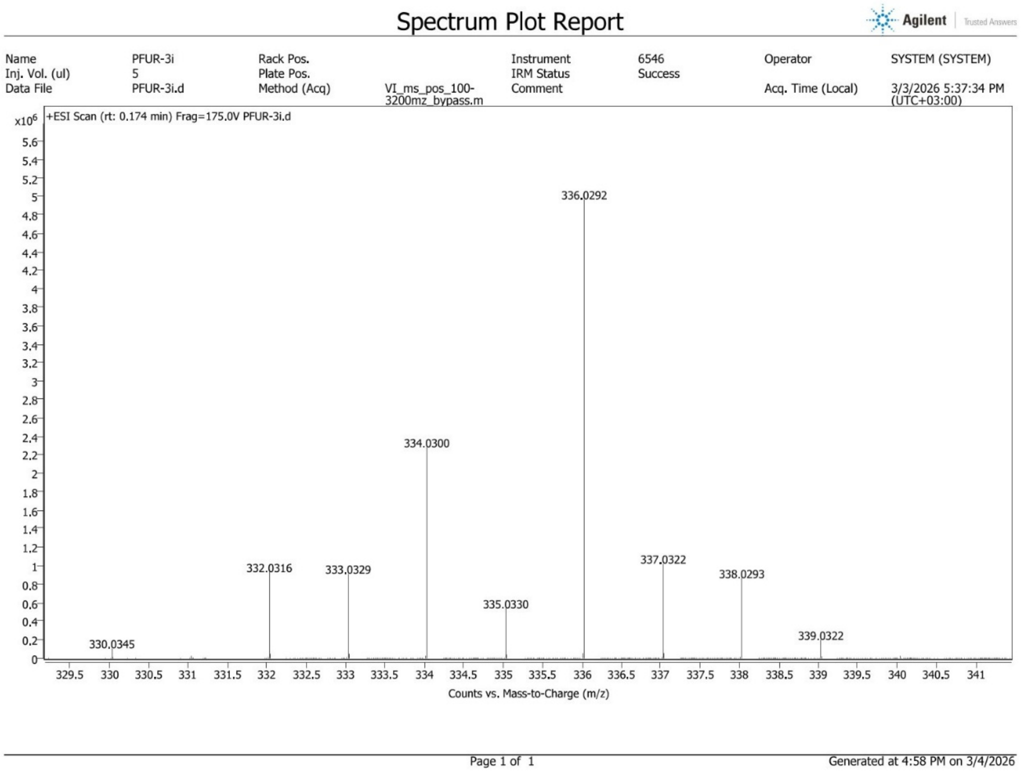

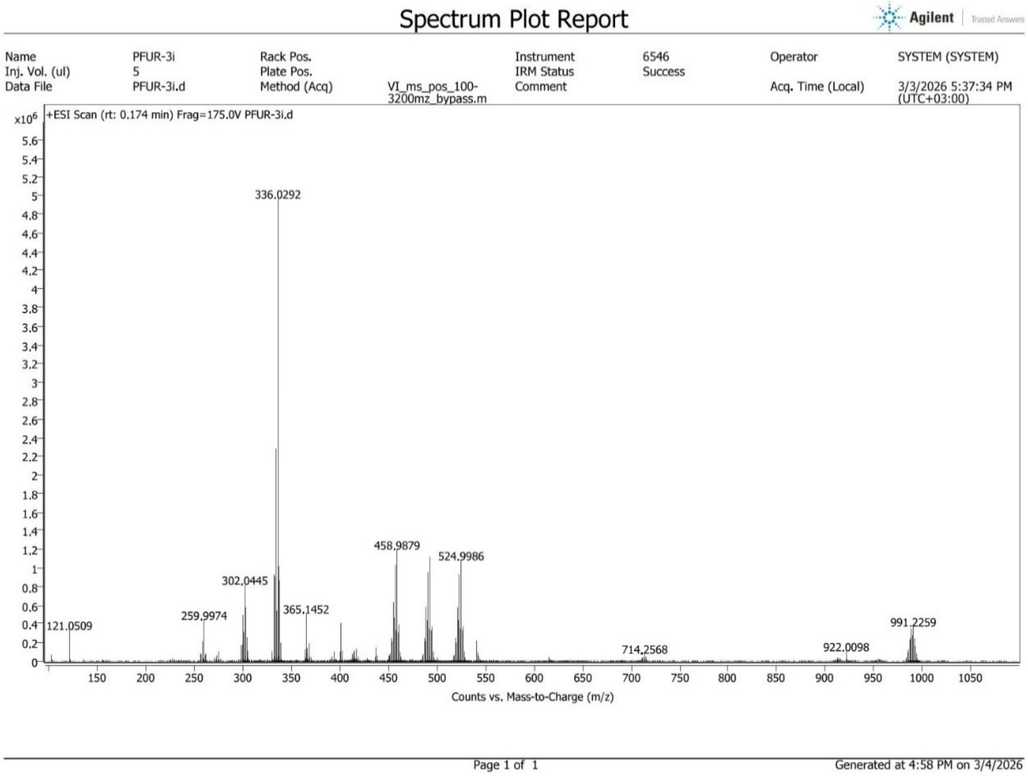

ESI HR-MS spectra of **3j**

Spectrum Plot Report

Name	PFUR-3j	Rack Pos.		Instrument	6546	Operator	SYSTEM (SYSTEM)
Inj. Vol. (ul)	5	Plate Pos.		IRM Status	Success		
Data File	PFUR-3j.d	Method (Acq)	VI_ms_pos_100-3200mz_bypass.m	Comment		Acq. Time (Local)	3/3/2026 5:40:17 PM (UTC+03:00)

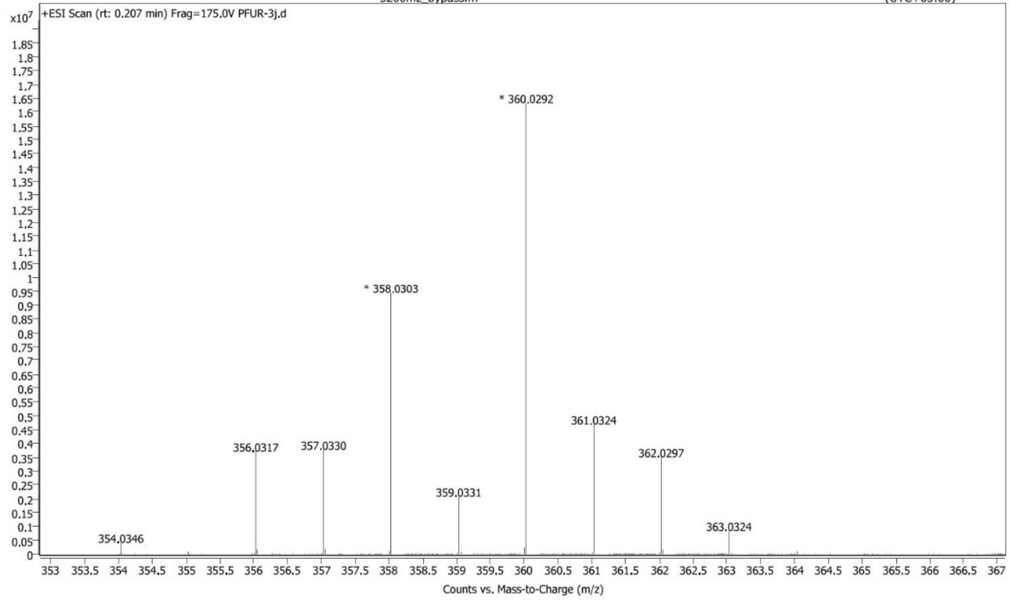

 Generated at 5:00 PM on 3/4/2026

Spectrum Plot Report

Name	PFUR-3j	Rack Pos.		Instrument	6546	Operator	SYSTEM (SYSTEM)
Inj. Vol. (ul)	5	Plate Pos.		IRM Status	Success		
Data File	PFUR-3j.d	Method (Acq)	VI_ms_pos_100-3200mz_bypass.m	Comment		Acq. Time (Local)	3/3/2026 5:40:17 PM (UTC+03:00)

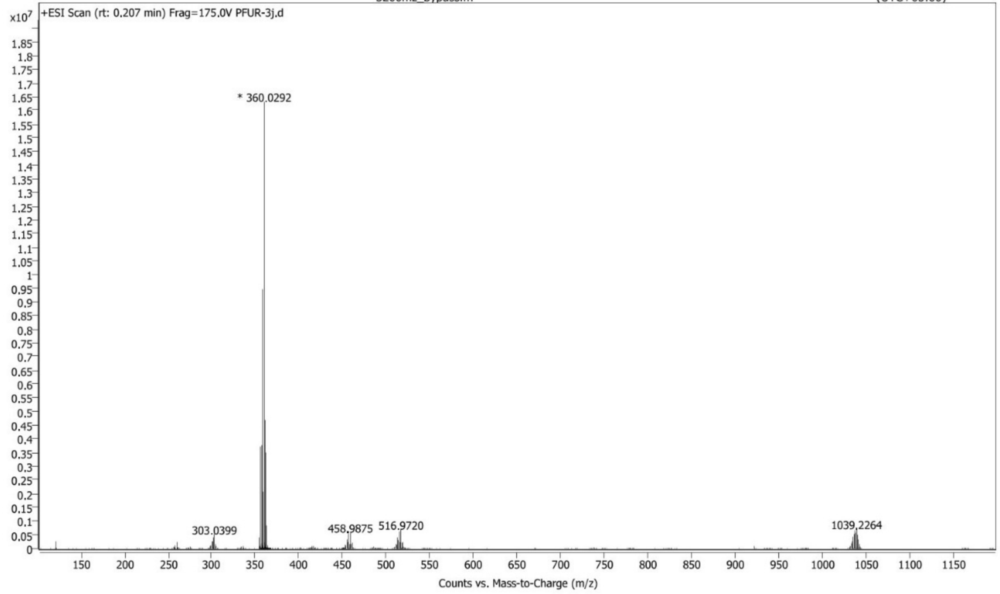

 Generated at 4:59 PM on 3/4/2026

ESI HR-MS spectra of **3k**

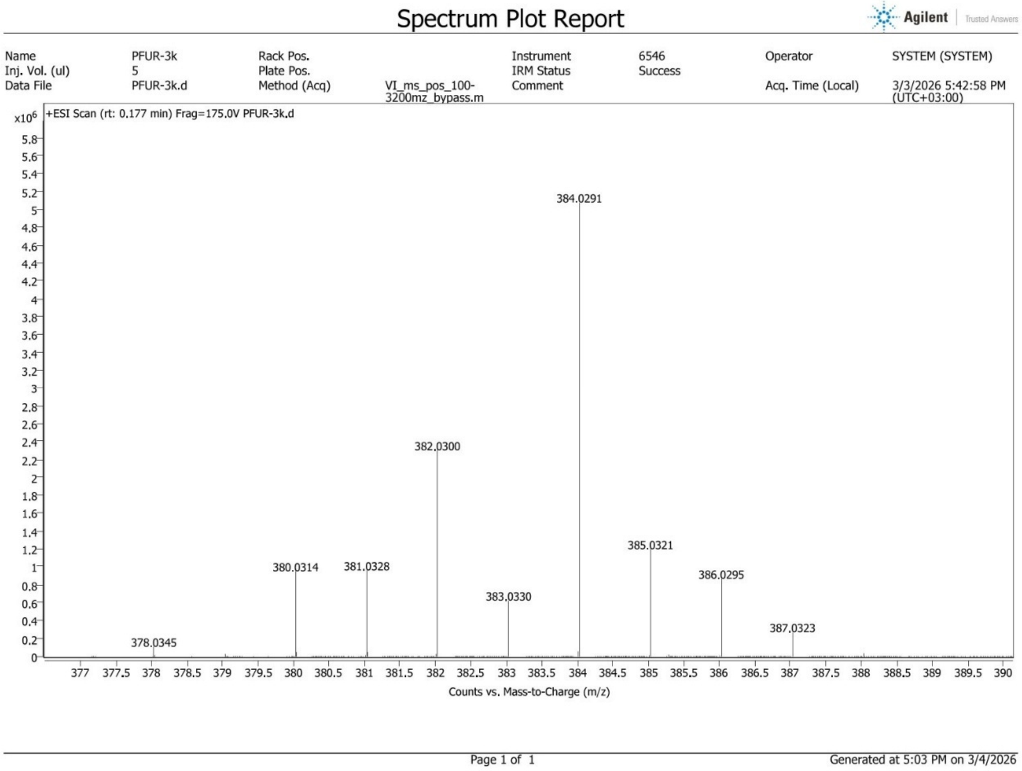

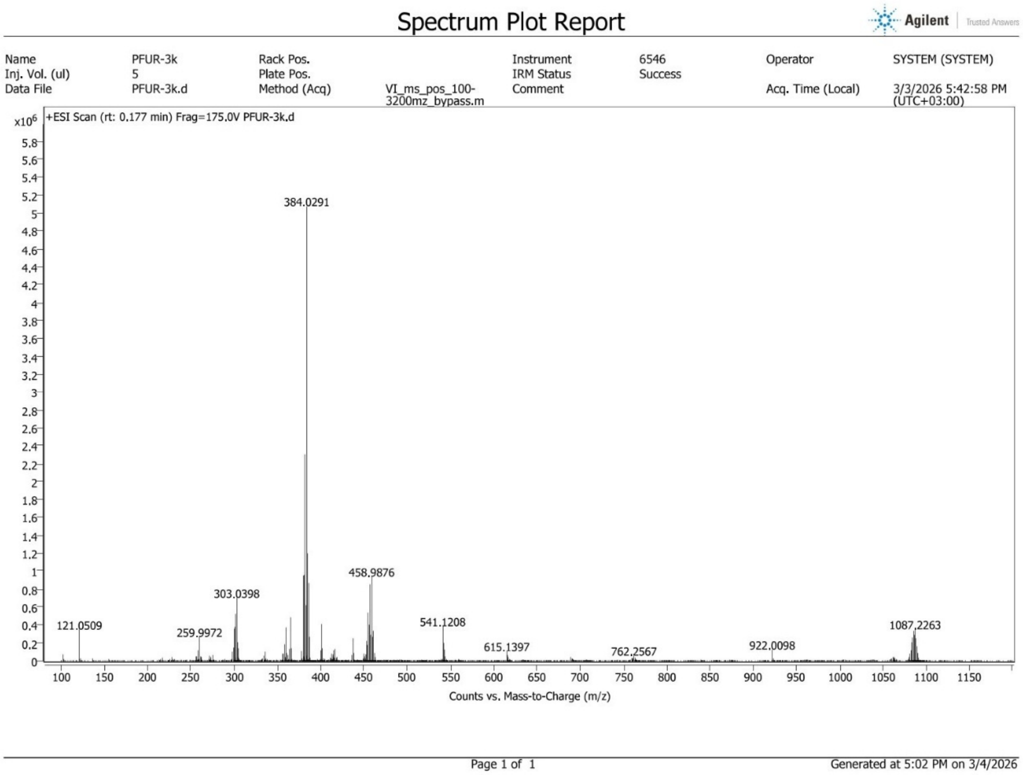

ESI HR-MS spectra of **3l**

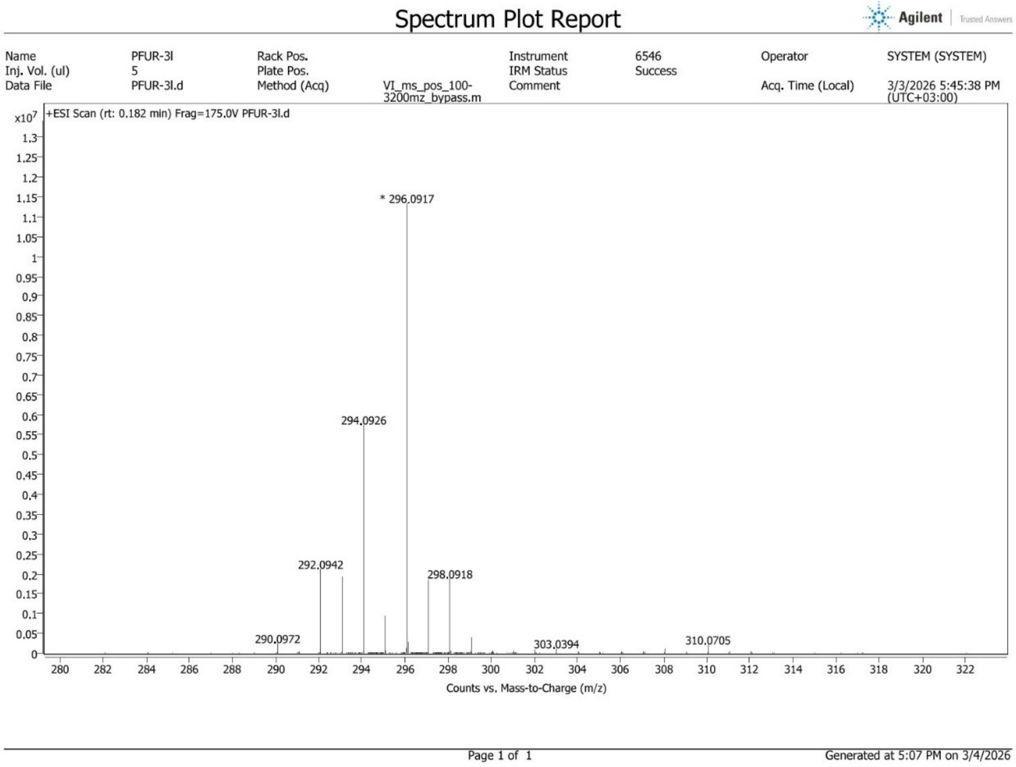

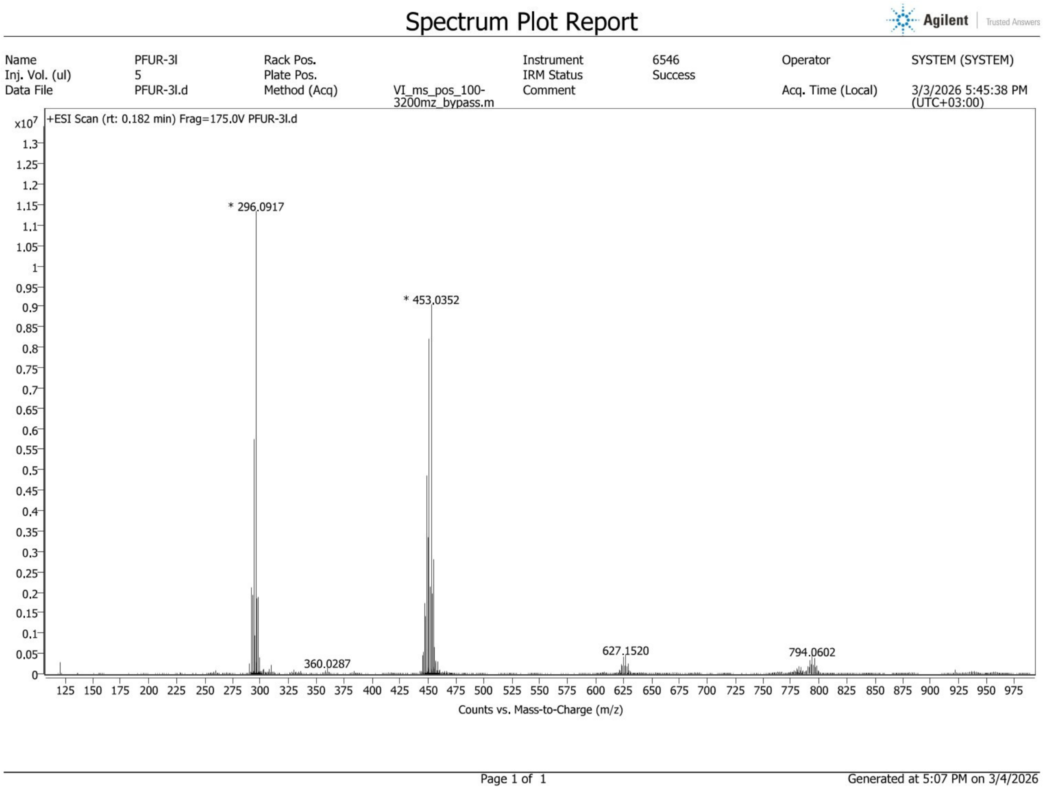

ESI HR-MS spectra of **3m**

Spectrum Plot Report

Agilent Trusted Answers

Name	PFUR-3m	Rack Pos.		Instrument	6546	Operator	SYSTEM (SYSTEM)
Inj. Vol. (ul)	5	Plate Pos.		IRM Status	Success		
Data File	PFUR-3m.d	Method (Acq)	VI_ms_pos_100-3200mz_bypass.m	Comment		Acq. Time (Local)	3/3/2026 5:48:20 PM (UTC+03:00)

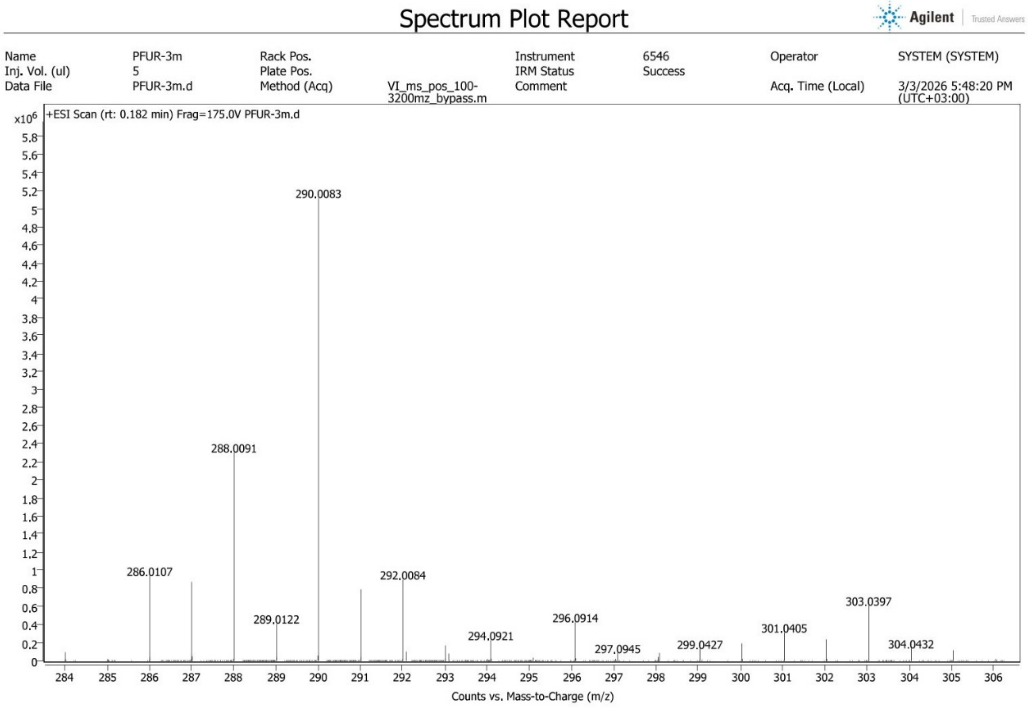

Spectrum Plot Report

Agilent Trusted Answers

Name	PFUR-3m	Rack Pos.		Instrument	6546	Operator	SYSTEM (SYSTEM)
Inj. Vol. (ul)	5	Plate Pos.		IRM Status	Success		
Data File	PFUR-3m.d	Method (Acq)	VI_ms_pos_100-3200mz_bypass.m	Comment		Acq. Time (Local)	3/3/2026 5:48:20 PM (UTC+03:00)

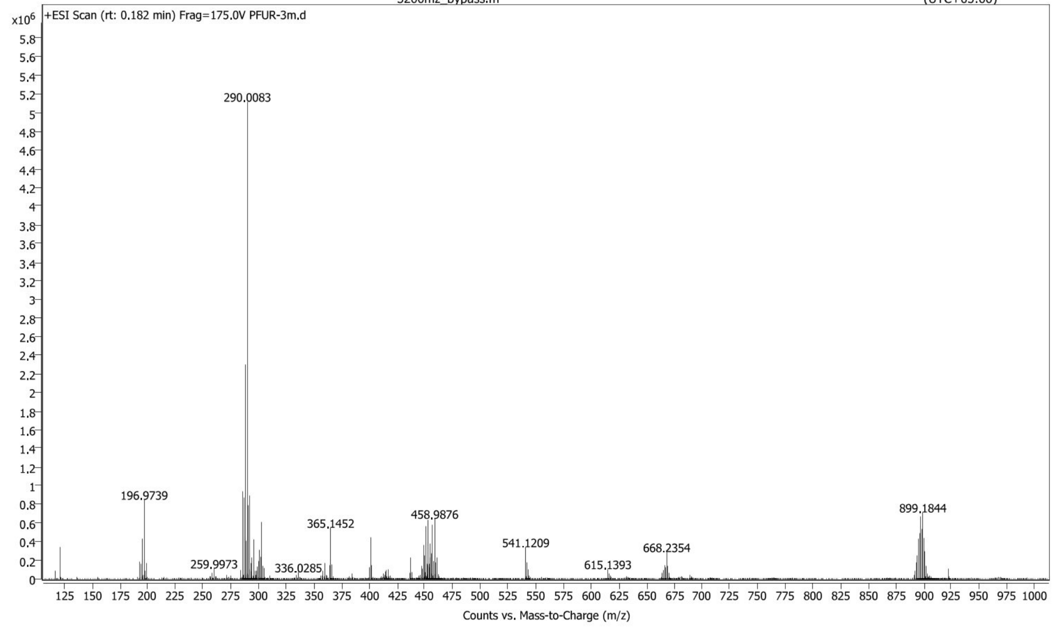

ESI HR-MS spectra of **3n**

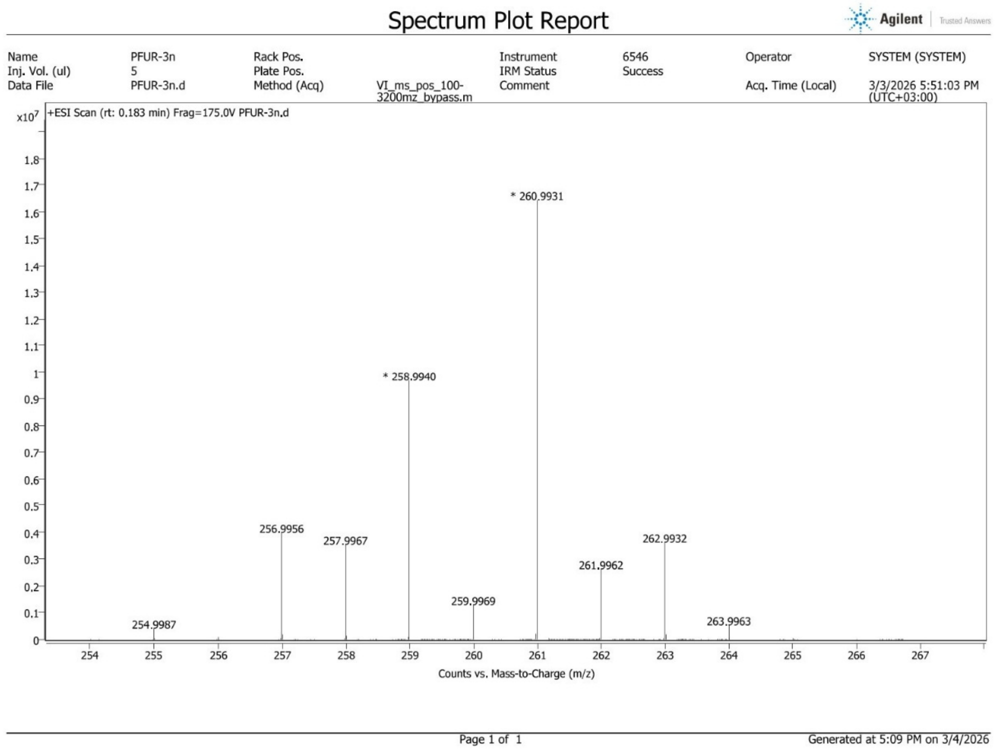

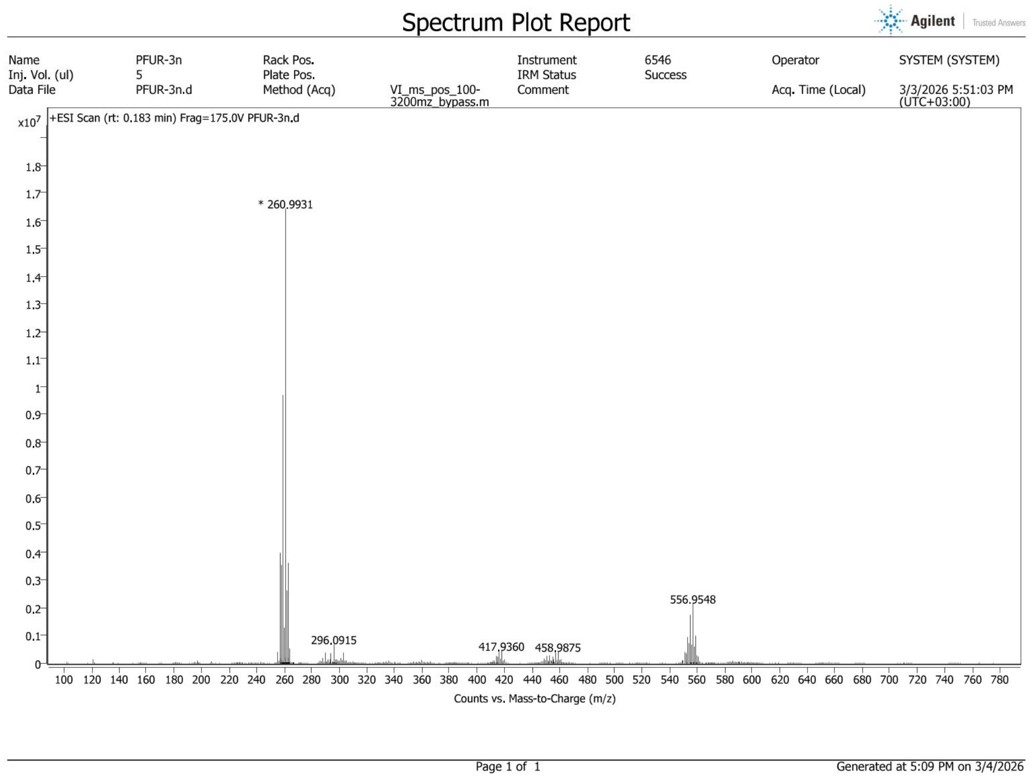

ESI HR-MS spectra of **3o**

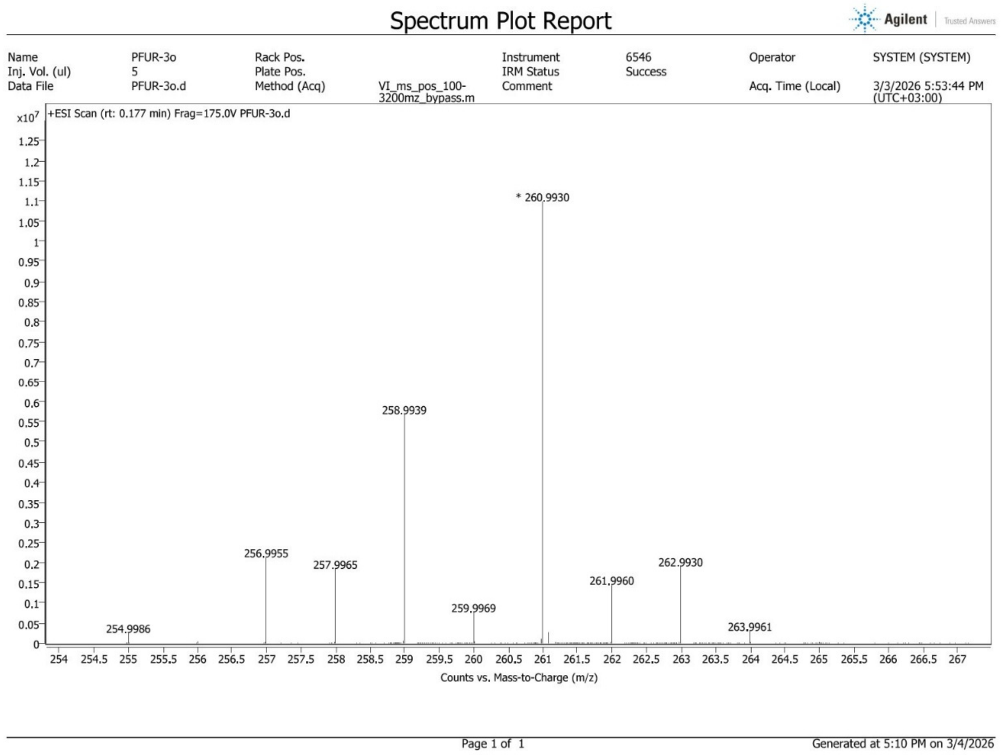

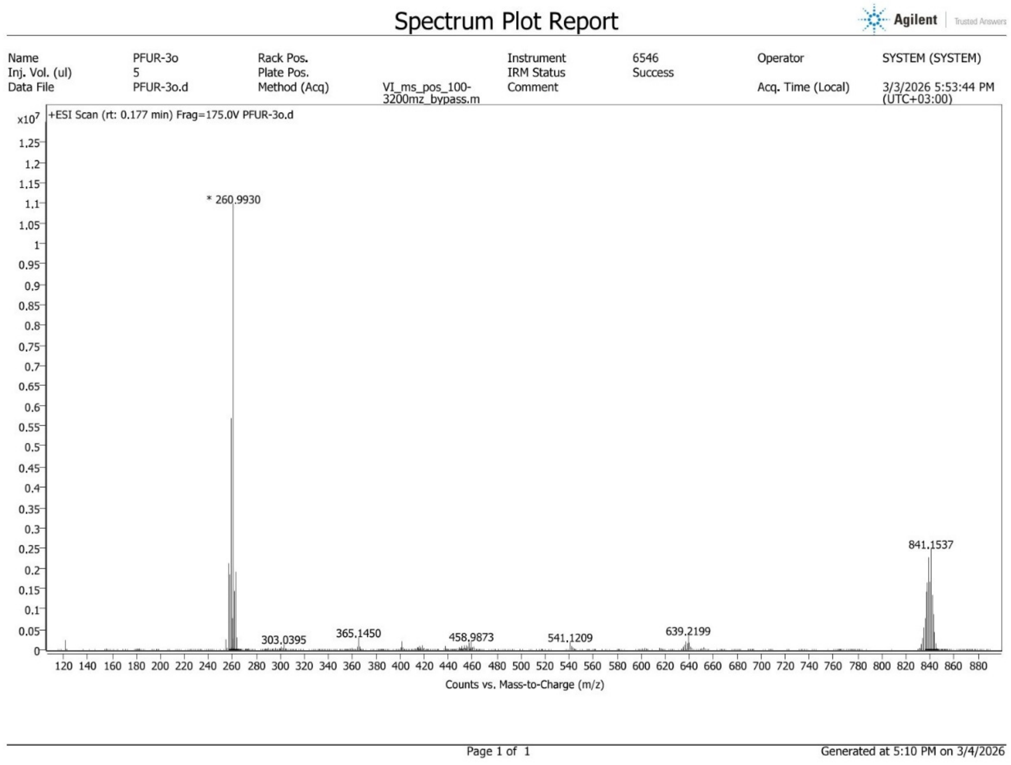

ESI HR-MS spectra of **3p**

Spectrum Plot Report

Name	PFUR-3p	Rack Pos.		Instrument	6546	Operator	SYSTEM (SYSTEM)
Inj. Vol. (ul)	5	Plate Pos.		IRM Status	Success		
Data File	PFUR-3p.d	Method (Acq)	VI_ms_pos_100-3200mz_bypass.m	Comment		Acq. Time (Local)	3/3/2026 5:56:26 PM (UTC+03:00)

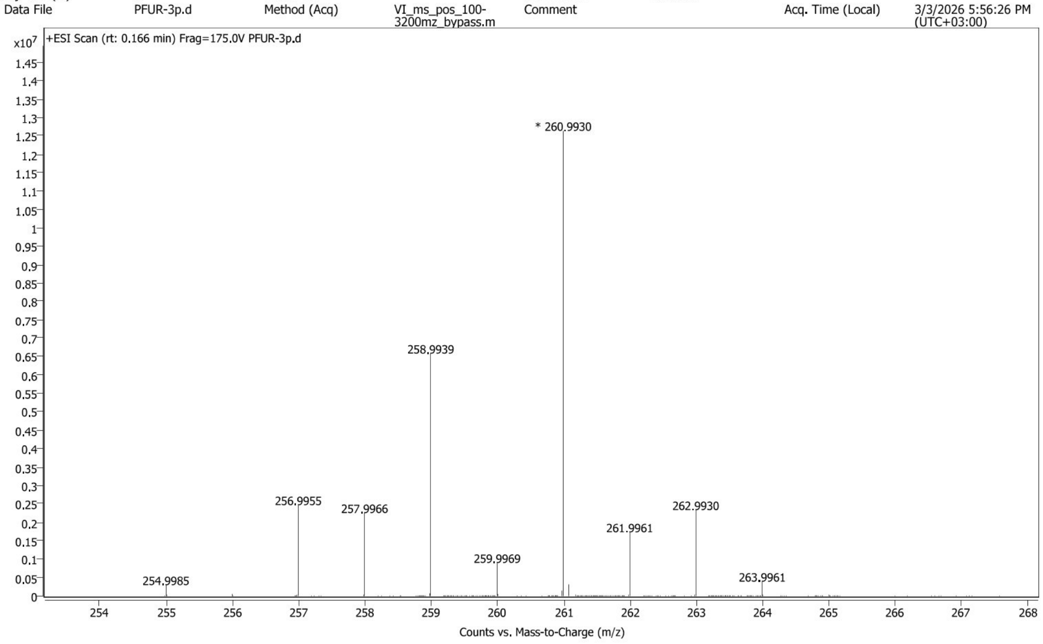

Generated at 5:11 PM on 3/4/2026

Spectrum Plot Report

Name	PFUR-3p	Rack Pos.		Instrument	6546	Operator	SYSTEM (SYSTEM)
Inj. Vol. (ul)	5	Plate Pos.		IRM Status	Success		
Data File	PFUR-3p.d	Method (Acq)	VI_ms_pos_100-3200mz_bypass.m	Comment		Acq. Time (Local)	3/3/2026 5:56:26 PM (UTC+03:00)

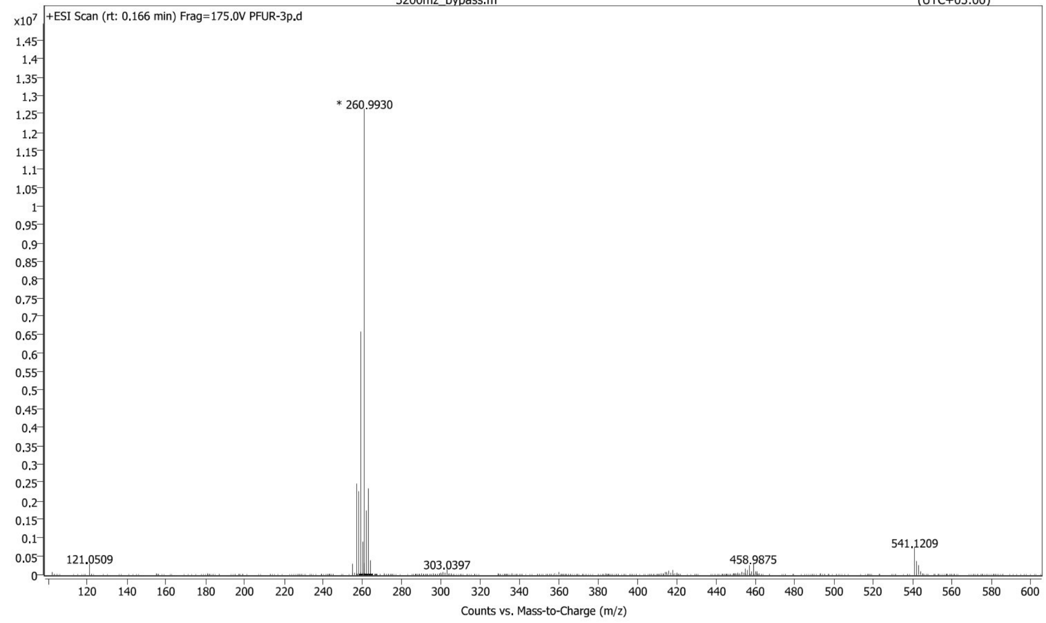

Generated at 5:11 PM on 3/4/2026

ESI HR-MS spectra of **3r**

Spectrum Plot Report

Name	PFUR-3r	Rack Pos.		Instrument	6546	Operator	SYSTEM (SYSTEM)
Inj. Vol. (ul)	5	Plate Pos.		IRM Status	Success		
Data File	PFUR-3r.d	Method (Acq)	VI_ms_pos_100-3200mz_bypass.m	Comment		Acq. Time (Local)	3/3/2026 5:59:06 PM (UTC+03:00)

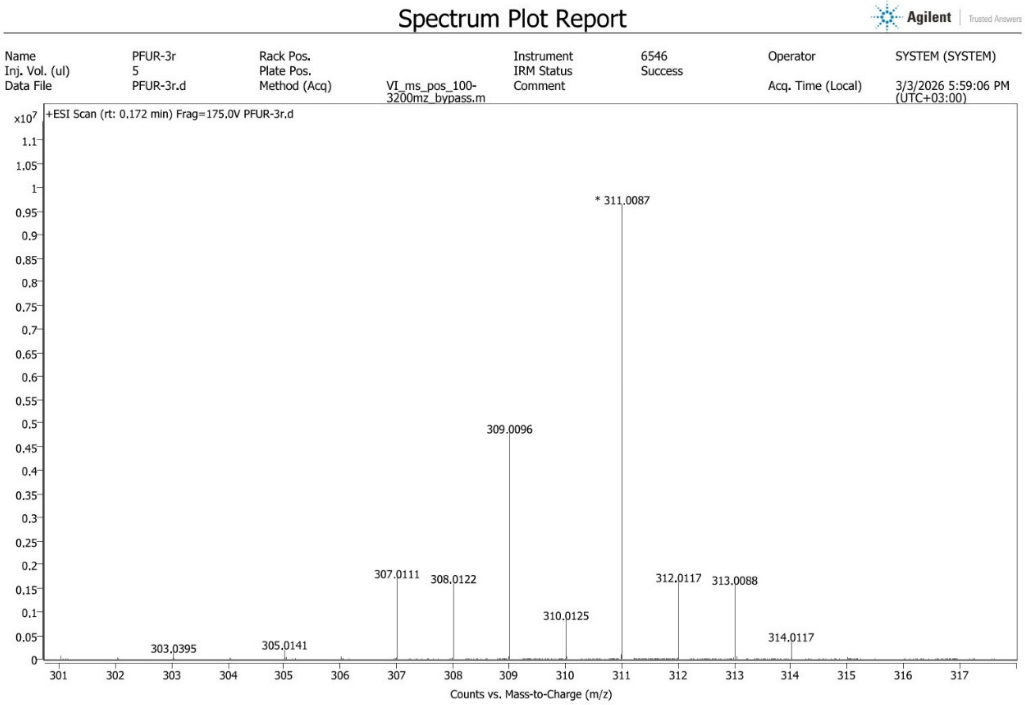

Spectrum Plot Report

Name	PFUR-3r	Rack Pos.		Instrument	6546	Operator	SYSTEM (SYSTEM)
Inj. Vol. (ul)	5	Plate Pos.		IRM Status	Success		
Data File	PFUR-3r.d	Method (Acq)	VI_ms_pos_100-3200mz_bypass.m	Comment		Acq. Time (Local)	3/3/2026 5:59:06 PM (UTC+03:00)

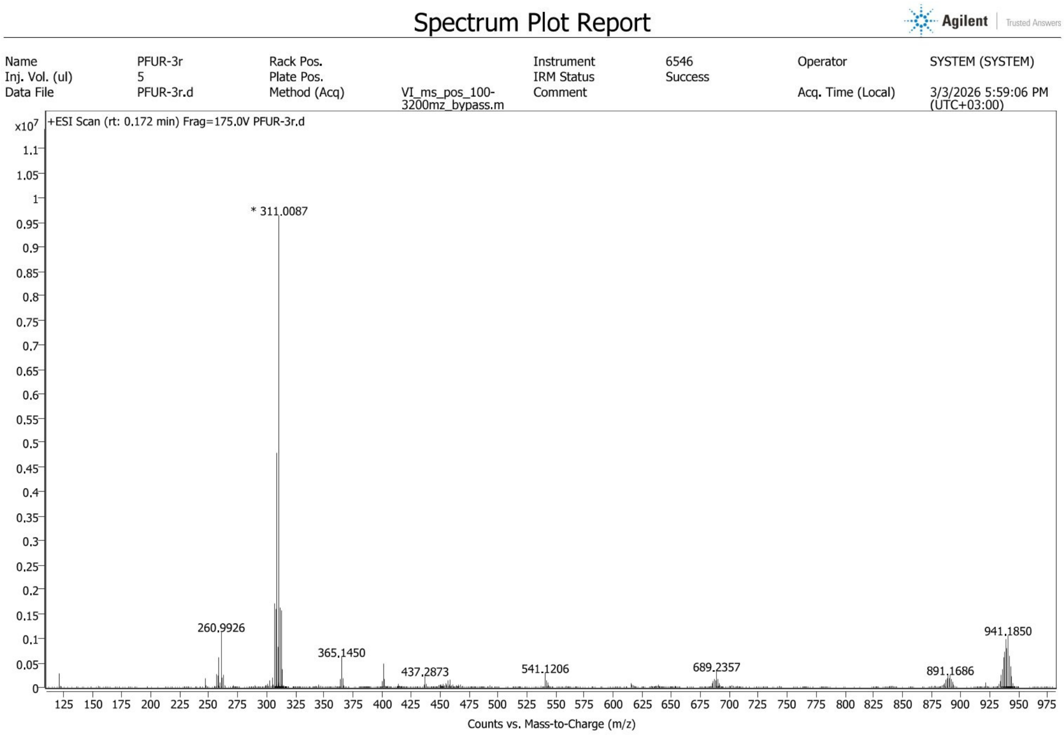

ESI HR-MS spectra of **3s**

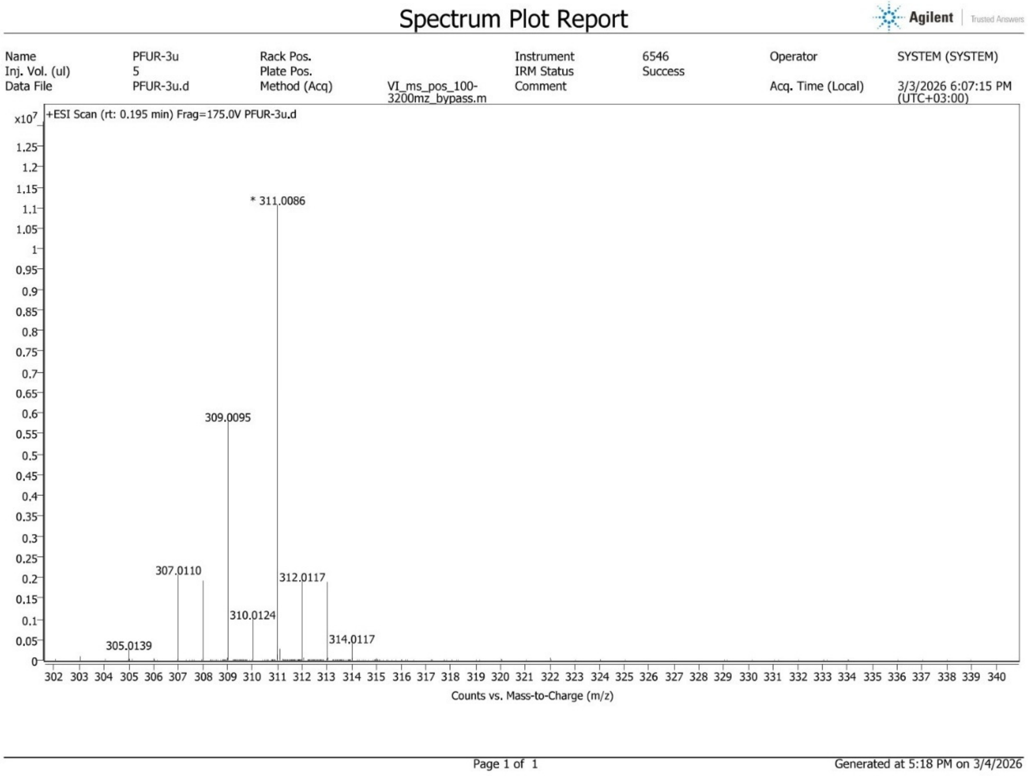

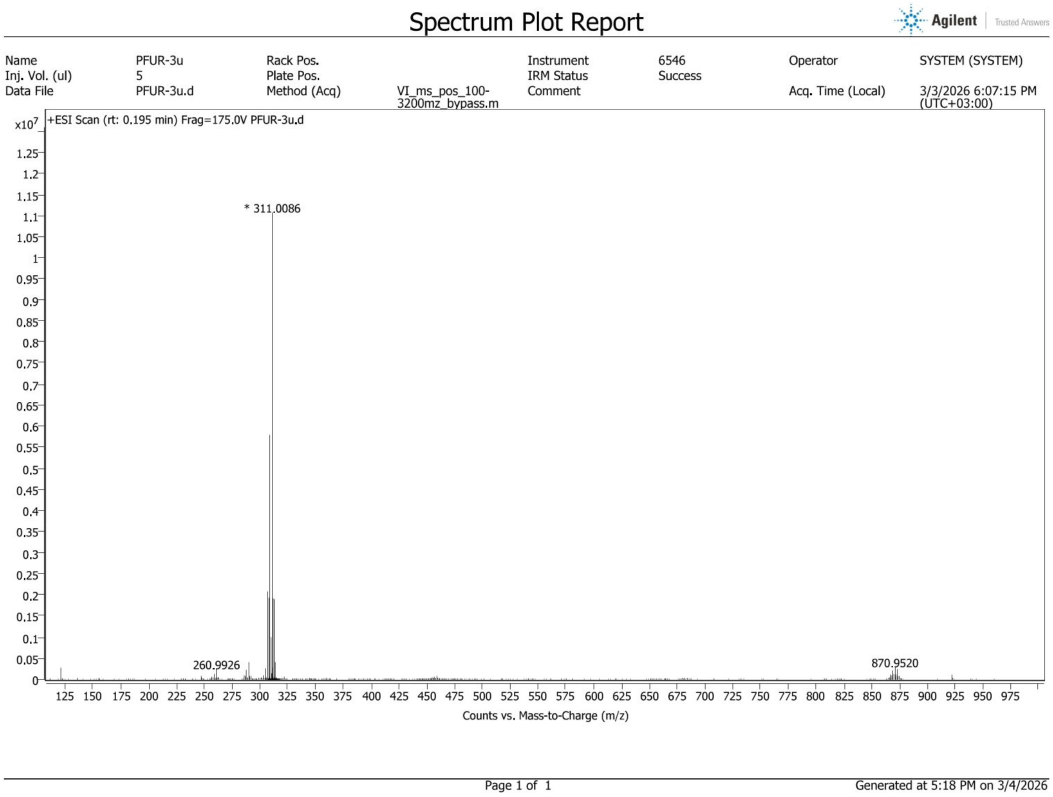

ESI HR-MS spectra of **3t**

Spectrum Plot Report

Agilent | Trusted Answers

Name	PFUR-3t	Rack Pos.		Instrument	6546	Operator	SYSTEM (SYSTEM)
Inj. Vol. (ul)	5	Plate Pos.		IRM Status	Success		
Data File	PFUR-3t.d	Method (Acq)	VI_ms_pos_100-3200mz_bypass.m	Comment		Acq. Time (Local)	3/3/2026 6:04:33 PM (UTC+03:00)

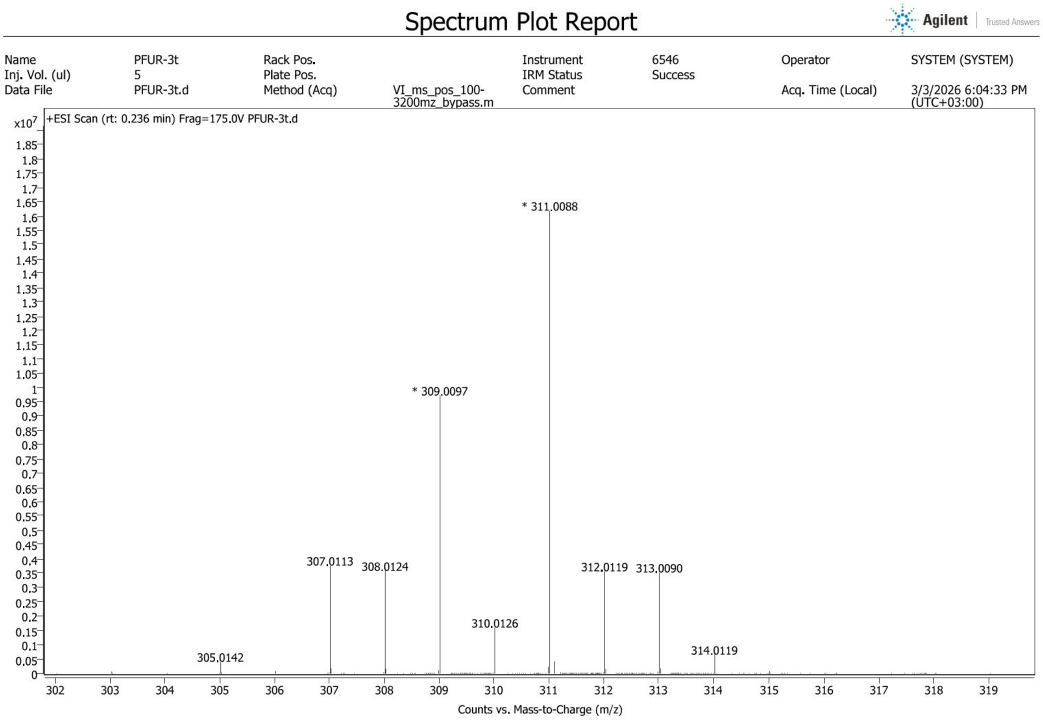

Generated at 5:18 PM on 3/4/2026

Spectrum Plot Report

Agilent | Trusted Answers

Name	PFUR-3t	Rack Pos.		Instrument	6546	Operator	SYSTEM (SYSTEM)
Inj. Vol. (ul)	5	Plate Pos.		IRM Status	Success		
Data File	PFUR-3t.d	Method (Acq)	VI_ms_pos_100-3200mz_bypass.m	Comment		Acq. Time (Local)	3/3/2026 6:04:33 PM (UTC+03:00)

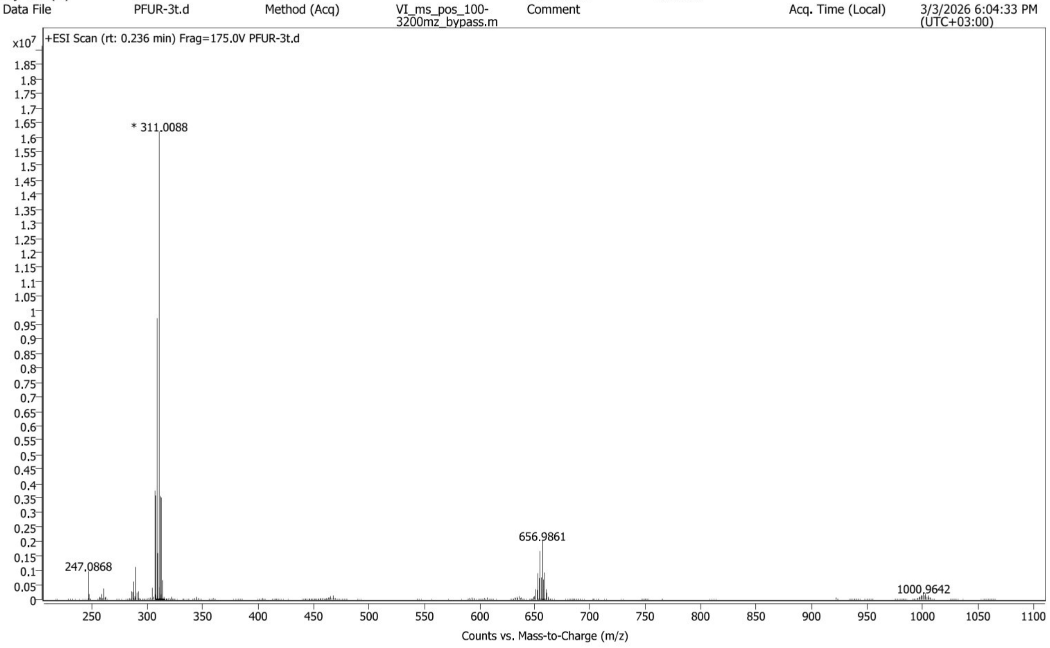

Generated at 5:17 PM on 3/4/2026

ESI HR-MS spectra of **3u**

Spectrum Plot Report

Name	PFUR-3a	Rack Pos.		Instrument	6546	Operator	SYSTEM (SYSTEM)
Inj. Vol. (ul)	5	Plate Pos.		IRM Status	Success		
Data File	PFUR-3a.d	Method (Acq)	VI_ms_pos_100-3200mz_bypass.m	Comment		Acq. Time (Local)	3/3/2026 5:15:56 PM (UTC+03:00)

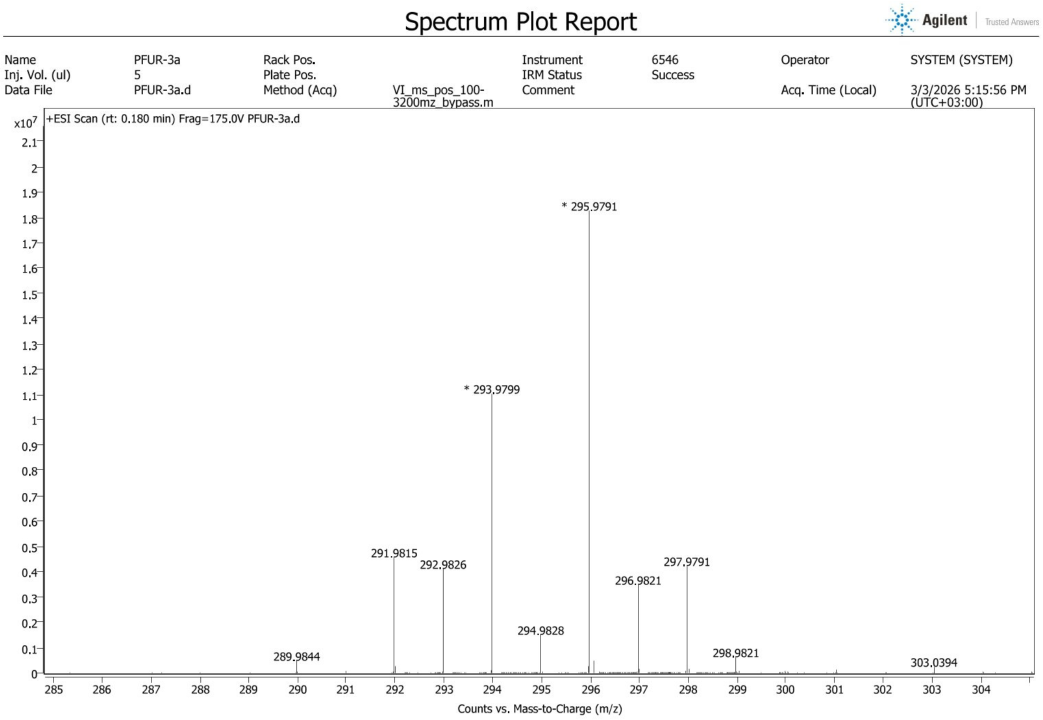

Page 1 of 1 Generated at 4:43 PM on 3/4/2026

Spectrum Plot Report

Name	PFUR-3a	Rack Pos.		Instrument	6546	Operator	SYSTEM (SYSTEM)
Inj. Vol. (ul)	5	Plate Pos.		IRM Status	Success		
Data File	PFUR-3a.d	Method (Acq)	VI_ms_pos_100-3200mz_bypass.m	Comment		Acq. Time (Local)	3/3/2026 5:15:56 PM (UTC+03:00)

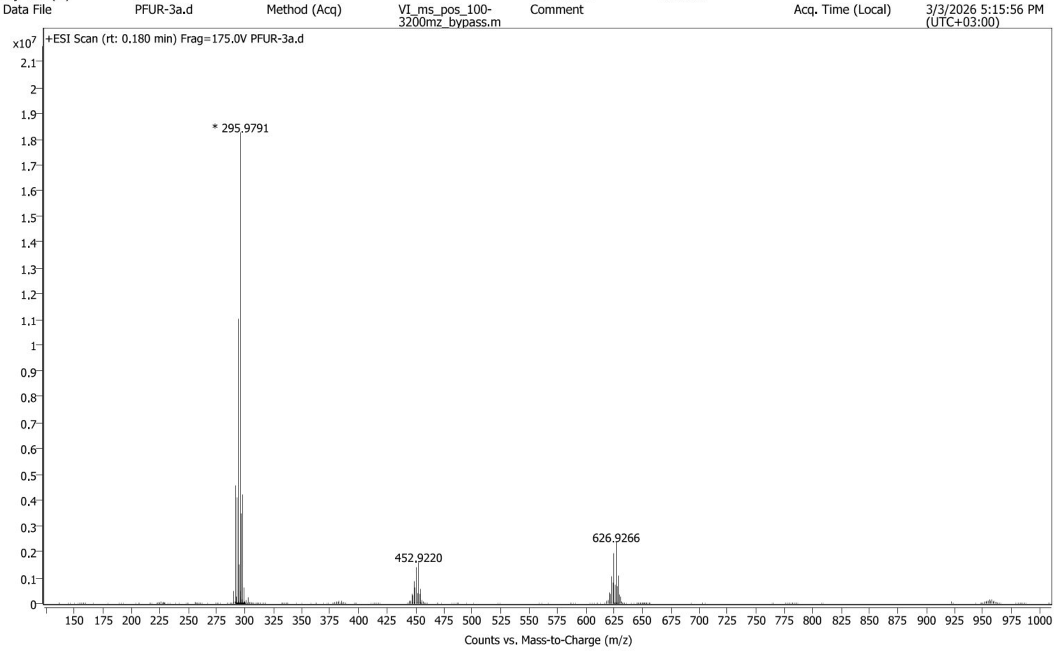

Page 1 of 1 Generated at 4:44 PM on 3/4/2026

ESI HR-MS spectra of **3v**

Spectrum Plot Report

Name	PFUR-3v	Rack Pos.		Instrument	6546	Operator	SYSTEM (SYSTEM)
Inj. Vol. (ul)	5	Plate Pos.		IRM Status	Success		
Data File	PFUR-3v.d	Method (Acq)	VI_ms_pos_100-3200mz_bypass.m	Comment		Acq. Time (Local)	3/3/2026 6:09:56 PM (UTC+03:00)

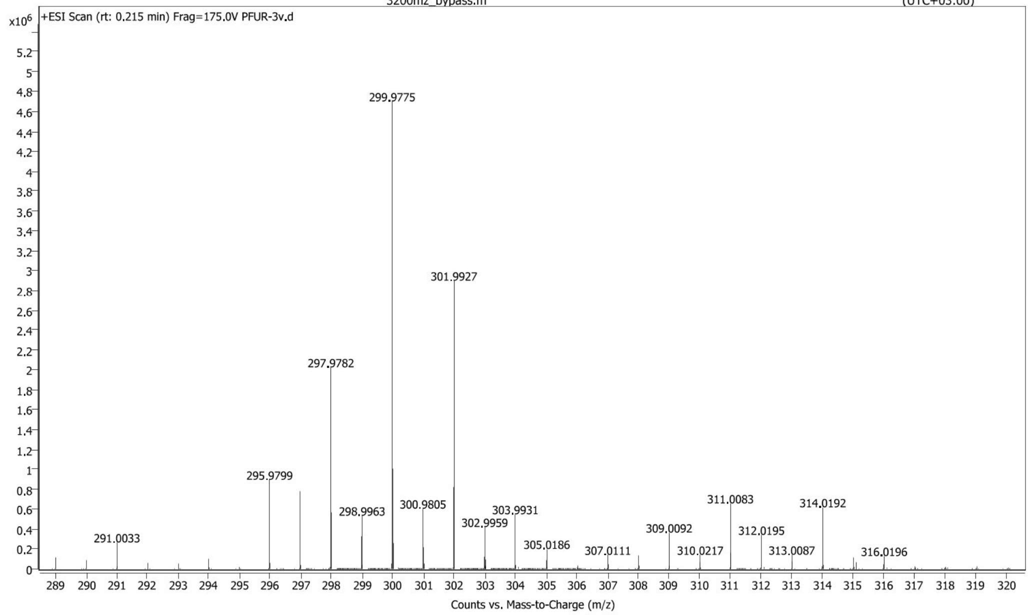

Spectrum Plot Report

Name	PFUR-3v	Rack Pos.		Instrument	6546	Operator	SYSTEM (SYSTEM)
Inj. Vol. (ul)	5	Plate Pos.		IRM Status	Success		
Data File	PFUR-3v.d	Method (Acq)	VI_ms_pos_100-3200mz_bypass.m	Comment		Acq. Time (Local)	3/3/2026 6:09:56 PM (UTC+03:00)

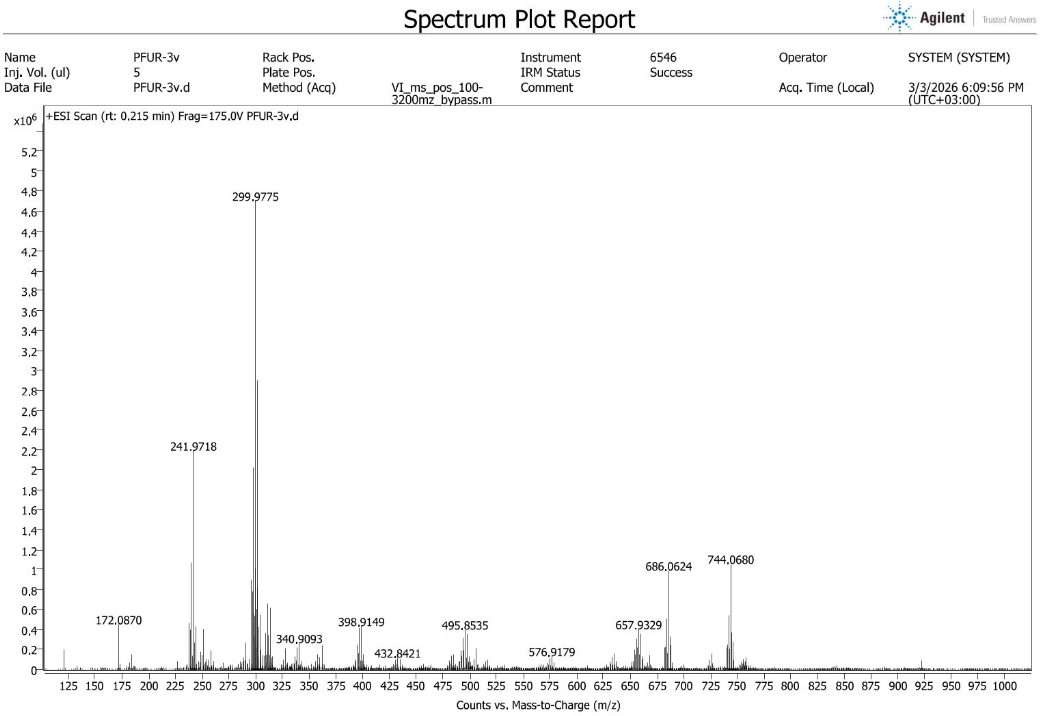

ESI HR-MS spectra of **3w**

Spectrum Plot Report

Name	PFUR-3w	Rack Pos.		Instrument	6546	Operator	SYSTEM (SYSTEM)
Inj. Vol. (ul)	5	Plate Pos.		IRM Status	Success		
Data File	PFUR-3w.d	Method (Acq)	VI_ms_pos_100-3200mz_bypass.m	Comment		Acq. Time (Local)	3/3/2026 6:12:36 PM (UTC+03:00)

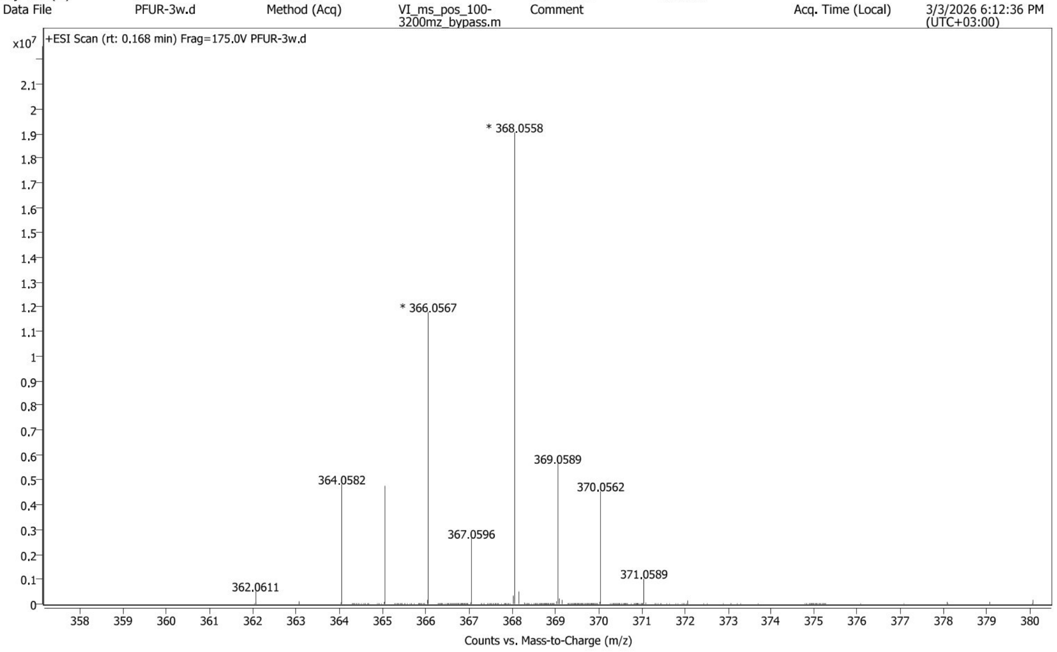

Spectrum Plot Report

Name	PFUR-3w	Rack Pos.		Instrument	6546	Operator	SYSTEM (SYSTEM)
Inj. Vol. (ul)	5	Plate Pos.		IRM Status	Success		
Data File	PFUR-3w.d	Method (Acq)	VI_ms_pos_100-3200mz_bypass.m	Comment		Acq. Time (Local)	3/3/2026 6:12:36 PM (UTC+03:00)

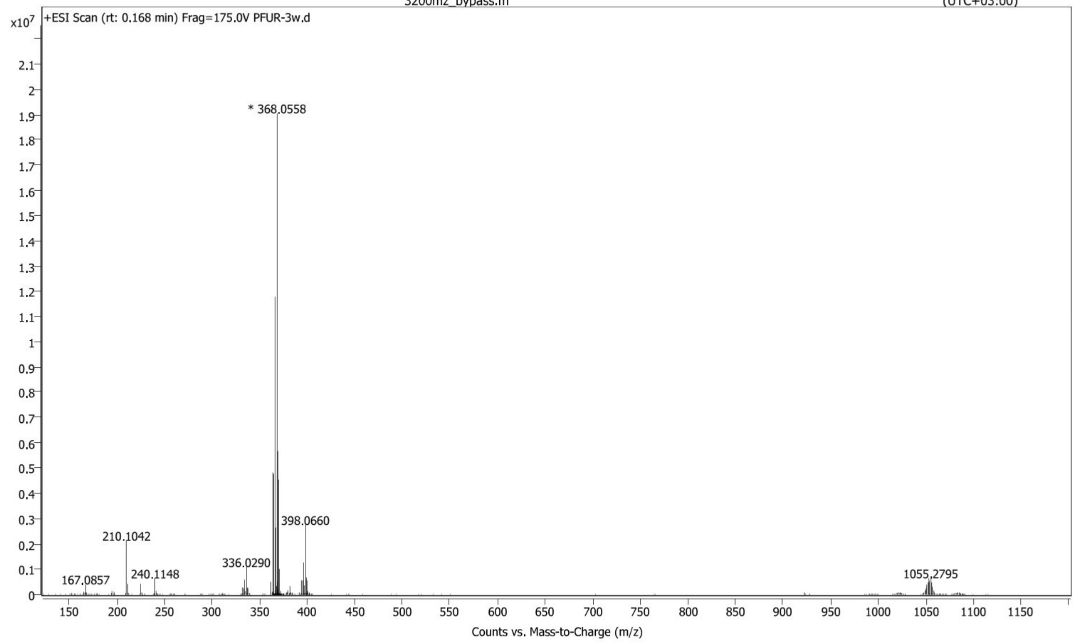

Supplement: Supplementary file 1 [file ijms-27-02908-s001.zip › ijms-4167030-supplementary.pdf]
